# Establishment of rapid saturation mutagenesis and screening methods for improving the neutralizing activity of monoclonal antibodies

**DOI:** 10.3389/fimmu.2025.1722831

**Published:** 2025-11-27

**Authors:** Xi Wu, Lijie Wang, Chenchen He, Yanru Shen, Youchun Wang, Weijin Huang

**Affiliations:** 1 Division of HIV/AIDS and Sex-Transmitted Virus Vaccines, Institute for Biological Product Control, National Institutes for Food and Drug Control (NIFDC), State Key Laboratory of Drug Regulatory Science, Beijing, China; 2 Clinical and Regulatory Affairs Center, Beijing Wantai Biological Pharmacy Enterprise Co., Ltd., Beijing, China; 3 School of Life Science and Biopharmaceutics, Shenyang Pharmaceutical University, Shenyang, China; 4 Chinese Academy of Medical Sciences & Peking Union Medical College, Beijing, China; 5 Institute of Medical Biology, Chinese Academy of Medical Science & Peking Union Medical College, Kunming, Yunnan, China

**Keywords:** saturation mutagenesis, monoclonal antibody, affinity optimization, pseudovirus neutralization assay, rabies virus

## Abstract

**Background:**

Monoclonal antibodies have been widely used in the fight against infectious diseases and are recommended for postexposure prophylaxis (PEP) of the rabies virus because of their advantages of easy supply, low cost, and high efficacy. To maximize treatment efficiency and minimize side effects, the binding affinity of isolated mAbs requires further enhancement.

**Methods:**

In this study, we developed a high-throughput saturation mutagenesis platform to improve the neutralizing activity of a preclinical antirabies mAb. We generated a system displaying antibodies on mammalian cell surfaces to block the invasion of the pseudotyped rabies virus. Saturation mutagenesis was performed on the complementarity-determining regions (CDRs) of the parental mAb, followed by screening for mutant mAbs with greater neutralizing activity.

**Results:**

We identified several mutations, including S5A, K18F, and N29C, that increased the neutralizing activity of the mAbs against six fixed strains of rabies virus, with average improvements of 4.92, 3.45, and 4.99 times, respectively. Furthermore, mAbs with the combined mutations S4I-P35S-N76D and S14H-T32N demonstrated significantly improved neutralizing activity.

**Conclusion:**

We established a high-throughput saturation mutagenesis platform for antibody affinity modification, enhancing the anti-infection ability of the antirabies NC08 antibody through single-point and multiple-point mutants. This platform is also applicable for optimizing other mAbs in preclinical stages in terms of neutralizing activity and achieving broad-spectrum activity.

## Introduction

Rabies is a zoonotic disease caused by rabies virus (RABV), which is capable of invading the central nervous system and causing fatal encephalitis (1). Despite great efforts in controlling transmission, rabies still results in tens of thousands of deaths each year (2). Postexposure prophylaxis (PEP) is the major strategy for preventing rabies, with procedures including wound washing, rabies immune globulin (RIG) administration and vaccination (3). The RIG provides important protection against infection before antibody production is stimulated by the vaccine. Equine rabies immune globulin (ERIG) and human rabies immune globulin (HRIG) are currently widely used in PEP (4), but more recently, monoclonal antibodies (mAbs) have been suggested as potentially superior alternatives, with easier supply, lower cost, and greater efficacy (5). An ideal monoclonal antibody treatment requires the binding of at least two different antibodies to nonoverlapping epitopes to broadly neutralize the virus. Moreover, the efficiency of the treatment should not be inferior to that of existing RIGs. Although several RABV mAbs have been approved for PEP (6), the binding affinity of mAbs still needs to be improved to achieve the goals of the rabies elimination project by 2030.

In general, mAbs can be generated via hybridoma technology or the sequencing of peripheral blood mononuclear cells (PBMCs) from convalescent patients. However, routine screening of antibodies requires further maturation steps to achieve high binding affinity, which can fulfill therapeutic requirements. Multiple strategies have been applied for *in vitro* antibody affinity maturation, including point mutation, saturation mutagenesis, and chain shuffling (7–9). Saturation mutagenesis is a semirational approach that introduces nonbiased mutations to generate variants with all the possible amino acid residues in one region. Commonly used saturation mutagenesis techniques utilize cassette mutagenesis, PCR amplification, gene splicing by overlap extension, and mutant plasmid amplification, followed by library screening and the selection of variants (7). Deep sequencing is subsequently performed to determine the variant read counts in the pre- and postselection populations. Consequently, the enrichment of mutants in the postselection population is calculated. High enrichment indicates that corresponding mutations are essential for a desired function (10). This technique has been widely used, significantly facilitating the development of mAbs for clinical use. During the COVID-19 pandemic, Jianzhong Xi et al. conducted research on saturation mutagenesis of the spike-interacting region and mAb CDR to optimize an ACE2 decoy receptor and a broad-spectrum antibody targeting SARS-CoV-2 shortly after the identification of new variants (9, 11). Sa Dong et al. performed site-saturation mutagenesis on a mAb for the detection of Cry1A (12), an insecticidal protein produced by the bacterium *Bacillus thuringiensis* (Bt), which is commonly used as a pesticide in genetically modified crops. The screening process was based mainly on the interaction affinity between the recombinant protein and mutant mAbs. However, some viral envelope proteins need to form polymers to achieve their physiological function, making it challenging to obtain a high yield and complicating purification, hampering the optimization of mAbs targeting these viruses.

Here, we established a new saturation mutagenesis and screening system to obtain mAbs with high neutralizing activity. We first generated a system that displays antibodies on the surface of mammalian cells. Then, we challenged the cells with a pseudotyped virus, which is a safe method that can mimic the infection process of live viruses. After verifying the ability of the antibody to display on the cell surface, we performed saturation mutagenesis on the complementarity-determining regions (CDRs) of the mAb and screened mutated mAbs with greater neutralization ability. On the basis of this approach, we optimized a preclinical antirabies mAb with potentially improved binding affinity for several rabies virus variants. We propose that this system is also applicable for optimizing other mAbs.

## Materials and methods

### Cells

HEK293T cells (American Type Culture Collection (ATCC), CRL-3216) were cultured in high-glucose Dulbecco’s modified Eagle’s medium (DMEM; HyClone, UT, USA) supplemented with 10% fetal bovine serum (FBS; Gibco, MT, USA) and 1% penicillin–streptomycin (Gibco) in a humidified atmosphere comprising 5% CO_2_ at 37°C.

### Generation of rabies virus pseudovirus

The replication incompetent RV pseudovirus was generated as follows: the G gene of the rabies virus was codon optimized for human cells and expressed from the pcDNA3.1 plasmid, which was used to transfect 293T cells via the Lipofectamine 3000 reagent (Invitrogen, L3000015, Carlsbad, CA, USA) following the manufacturer’s instructions. Moreover, the transfected cells were infected with G gene-deleted (G*ΔG) VSV, which harbored either a GFP or a luciferase reporter gene, at a multiplicity of infection (MOI) of 4. At 6 h post infection, the supernatants were removed, and the cells were washed with 2% FBS/PBS three times, after which fresh DMEM supplemented with 10% FBS was added. At 24 h postinfection, the supernatants containing the VSV-RV were harvested, filtered through a 0.45 μm pore-size membrane, and stored at −70°C until use. We used G gene of CVS-N2C to construct replicate incompetent pseudotyped virus to confirm function of NC08 scFv-CAR and infect cells expressing NC08 scFv-CAR libraries. G protein of six fixed strains, including PM(Protein Accession Number:CAI43218.1), PV(Protein Accession Number: P08667.1), CTN-1V(Protein Accession Number:ACR39382.1), aGV(Protein Accession Number:ADM32132.1), Flury-LEP(Protein Accession Number: ADD84785.1), CVS-N2C(Protein Accession Number:ADJ29911.1)and eight street strains: protein accession number including AIL01046.1, ADM63799.1, AGN94375, AKN8964, ACR50734, AGE31951, AGN94476, AFM52598.1 were used for neutralization assays.

Replicate competent RV pseudoviruses were rescued from cDNA clones as previously described (13). Briefly, a plasmid encoding the T7 promoter for the VSV antigenome was modified by replacing the original G gene with that of the rabies virus CVS strain, and a GFP reporter gene was inserted into the genome as a separate transcriptional unit before the L gene expression cassette was constructed. Plasmid-based rescue of the replication-competent VSV-based pseudovirus was carried out as described previously (13). Briefly, BHK21 cells were infected with the vaccinia virus vTF7-3, which expresses T7 polymerase, for 2 h. Then, the supernatant was discarded, and the cells were transfected with a plasmid encoding the VSV antigenome, along with helper plasmids encoding the T7 promoter for the VSV N, P, G, and L genes, via the Lipofectamine 3000 reagent. The transfected cells were cultured for an additional 48 h, after which the supernatants were collected, filtered through a 0.22 μm pore-size membrane, and passaged in 293T cells. The virus was further passaged and amplified three to four times every 2–3 days. The viral RNA was then extracted, and reverse transcription PCR was performed. The full-length spike gene was amplified and sequenced. The supernatant containing the virus was aliquoted and stored at −80°C.

### Construction of the scFv-CAR expression plasmid

A signal peptide was added to the N-terminus of the mAb, followed by a 3×Flag tag to ensure proper transport, and the transmembrane domain adopted from the structure of CAR-T cells was fused to the C-terminus. In addition, mCherry was fused with the T2A sequence at C-terminal. This cassette was cloned into pcDNA3.1(+) vector under the control of CMV promoter.

### Construction of the CDR deep mutation scanning library

Two oligo libraries targeting the VL (33aa) and VH (40aa) domains of the NC08 scFv were designed. The libraries consisted of a total of 1387 oligos. The mutation sequence was flanked by *Bsp*QI restriction enzyme sites and the common sequence for PCR amplification. The oligo library was amplified and digested with *Bsp*QI to release the mutagenic primers. The NC08 ScFv-CAR saturation mutation pool was generated via circular PCR using mutagenic primers followed by digestion of the template plasmid with *Dpn*I. The mutated plasmids were then amplified in *E*. *coli* DH5α and extracted. To verify the coverage of the mutation pool, the CDR fragments of VL and VH were amplified and subjected to high-throughput sequencing.

### Screening of high-affinity mutants

The cells were transfected with the NC08 scFv-CAR saturated mutation pool and infected with 5×10^7^ focus-forming units (FFUs) of CVS-GFP replicative pseudovirus. After 24 hours of culture, the mCherry^+^ GFP^-^ cells were sorted, and the NC08 scFv-CAR mutants effectively prevented virus entry due to increased neutralization ability. The RNA of these cells was extracted, reverse-transcribed and PCR amplified to obtain VH-CDR and VL-CDR mutant sequences through next-generation sequencing. Unsorted cells were used as the control group. Eight parallel experiments were performed.

### Pseudovirus neutralization assay

A pseudovirus neutralization assay was performed to evaluate the neutralizing ability of the antibodies. The detailed process has been described previously (14). Briefly, serially diluted antibodies were first incubated with the indicated pseudotyped virus for 1 h, and the mixture was then incubated with HEK293T cells for 24 h at 37°C. Subsequently, lysis buffer containing luciferase substrate (PerkinElmer) was added, followed immediately by luminescence intensity measurement in a microplate reader.

### Statistical analysis

All the data were analyzed, and graphs were generated via GraphPad Prism 8.0 software (GraphPad Software, Inc., San Diego, CA, USA). Student’s *t* test was used to assess the statistical significance of differences in the RLU and FFU of cells transfected with NC08-VHVLdel-CAR or NC08-CAR and infected with CVS rabies pseudoviruses expressing either firefly luciferase or the GFP reporter at each virion dilution. Differences with P values < 0.05 were considered statistically significant and are indicated by asterisks.

## Results

### Cell-surface display of the anti-rabies mAb and functional validation

**Figure 1:**
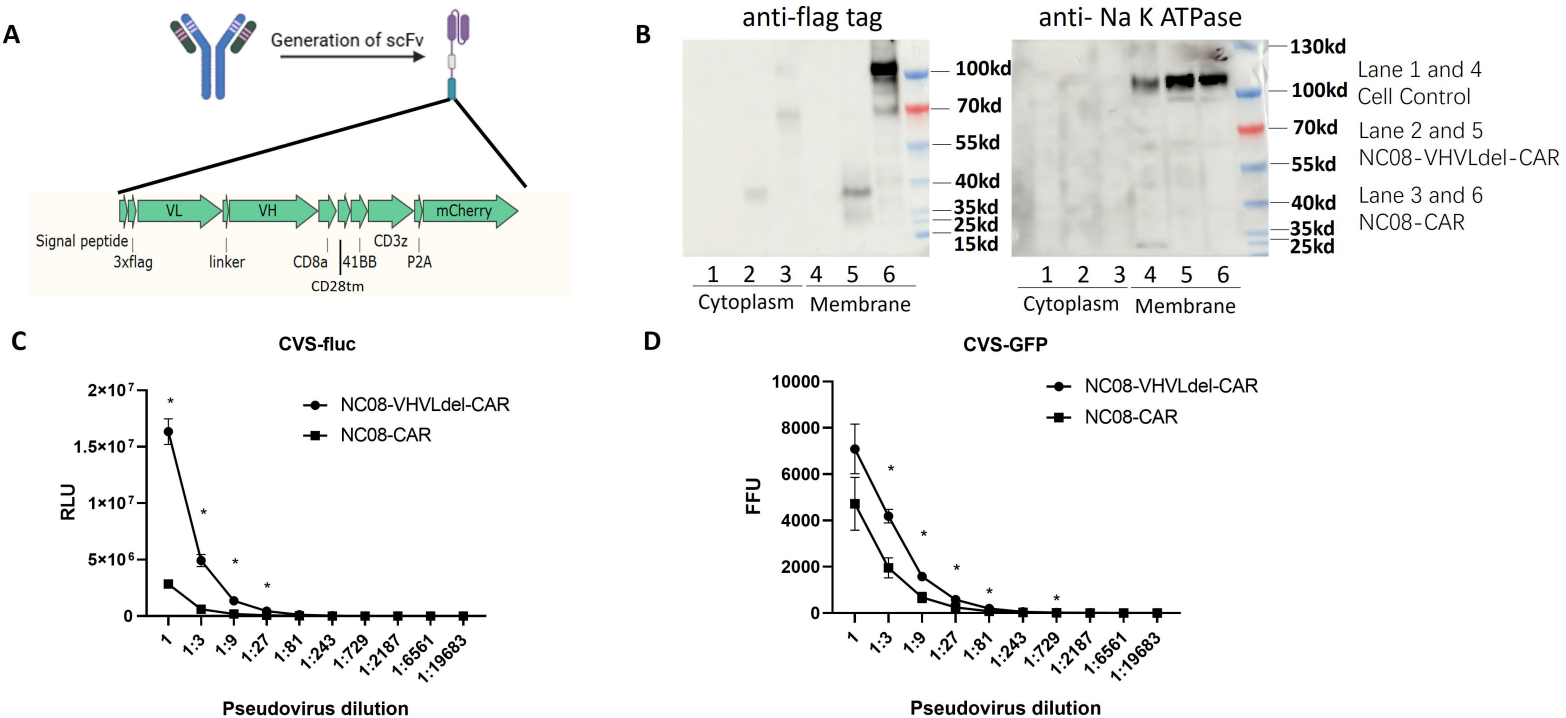
Generation of the NC08 scFv-CAR and its functional analysis. **(A)**. Schematic diagram of the antibody scFv surface display system. The variable regions of the light and heavy chains were fused via a linker (3×GGGS). A signal peptide was added to the N-terminus of the mAb, followed by a 3×Flag tag to ensure proper transport. The transmembrane domain adopted from the structure of CAR-T cells was fused to the C-terminus, and mCherry was fused with a T2A sequence at the N-terminus of the transmembrane domain. **(B)**. Plasmids expressing NC08-CAR (lanes 3 and 6) or NC08-VHVLdel-CAR (lanes 2 and 4) were used to transfect 293T cells. Cytoplasmic and membrane protein fractions were collected, and the localization of the NC08-CAR was confirmed by western blotting with an anti-FLAG monoclonal antibody. The separation of the cytoplasm and the membrane was confirmed by the membrane marker Na, K ATPase **(C, D)**. The function of the NC08 scFv was verified by blocking infection with the CVS strain rabies pseudovirus encoding a firefly luciferase reporter **(C)** or a GFP reporter **(D)**. The lower relative luminescence units (RLUs) or focus-forming units (FFUs) indicated that fewer cells were infected by the CVS strain of rabies pseudovirus, which was blocked by the NC08 scFv on the cell surface. Statistical analysis of the differences in the RLU **(C)** and FFU **(D)** of NC08-VHVLdel-CAR and NC08-CAR at each virion dilution. Significant differences (p < 0.05) are indicated with asterisks.

To develop a system to display and assess the binding ability of anti-rabies mAbs, we constructed an expression cassette for the single-chain fragment variable (scFv) of the anti-rabies NC08 mAb (North China Pharmaceutical Co., Ltd.), which is a component of an antibody regimen in phase III clinical trial. A signal peptide was added to the N-terminus of the mAb, followed by a 3×Flag tag to ensure proper transport, and the transmembrane domain adopted from the structure of CAR-T cells was fused to the C-terminus. In addition, mCherry was fused with the T2A sequence, enabling the screening of antibody-expressing cells without influencing the location and function of the NC08 scFv (**Figure 1A**). To confirm that the NC08 scFv was correctly expressed and located on the cell membrane, a plasmid expressing the NC08 scFv was used to transfect HEK293T cells. The cytoplasmic and membrane protein fractions were separated and subjected to western blot analysis, which confirmed that the NC08 scFv was located mainly on the cell membrane (**Figure 1B**). Furthermore, to evaluate whether the NC08 scFv is capable of binding the rabies glycoprotein and blocking virus infection, the cells were challenged with a pseudotyped rabies virus encoding either firefly luciferase or GFP as a reporter (**Figure 1C**). Compared with control cells expressing NC08 scFv variants with deletions of VH and VL (variable light and heavy domains), cells expressing the whole NC08 scFv presented a significantly greater capacity to defend against pseudotyped virus infection. Taken together, these results verified that NC08 was modified into scFv and displayed on the cell surface, where it was functional in neutralizing pseudotyped rabies virus infection.

### Generation of the NC08 ScFv-CAR saturated mutation pool

**Figure 2:**
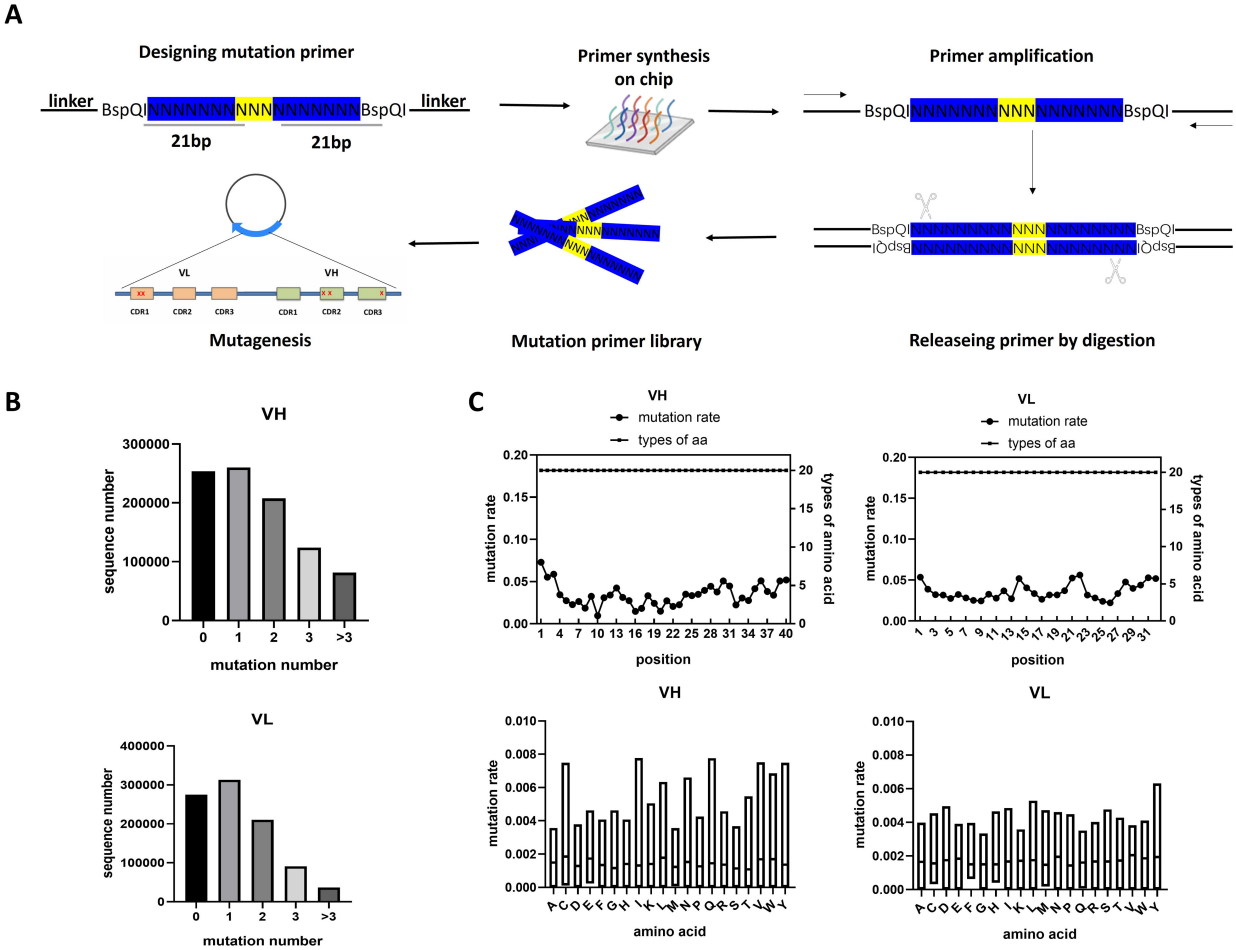
Generation of the NC08 ScFv-CAR saturated mutation pool. **(A)** Generation of primers for high-throughput saturation mutagenesis. High-throughput chip-synthesized oligos were used to produce saturating mutagenic primers for NC08 scFv. **(B)** Distribution of VH and VL variants harboring different numbers of mutations in the plasmid pools. **(C)** Mutation rates and types of different amino acids at every position in the CDRs of VH and VL. **(D)** The abundance of each amino acid in the CDRs of VH and VL revealed that no mutation bias was introduced during the construction of the saturation library.

Since antigen binding is governed mainly by three complementarity-determining regions (CDRs) within variable light (VL) and heavy (VH) domains, we designed and synthesized two oligo libraries targeting the VL (33aa) and VH (40aa) domains of the NC08 scFv protein. The libraries consisted of a total of 1387 oligos, including targeted regions for mutagenesis. The mutagenic region was flanked by *Bsp*QI restriction enzyme digestion sites and a common sequence for PCR amplification. The oligo library was amplified and digested by *Bsp*QI to release the mutagenic primers (**Figure 2A**). The NC08 ScFv-CAR saturated mutation pool was generated via circular PCR via mutagenic primers followed by digestion of the template plasmid with *Dpn*I. The mutated plasmids were then amplified in *E*. *coli* and extracted. To verify the coverage of the mutation pool, the CDR fragments of VL and VH were amplified and subjected to high-throughput sequencing. Approximately 25% of the plasmids contained the original sequence, 30% carried one mutation, and the remaining plasmids had two or more mutations (**Figure 2B**). The average number of CDR mutation sites in the VH library was 2.1 mutations/120 bp, whereas the average number of mutation sites in the VL library was 1.79 mutations/96 bp. In addition, we evaluated the coverage and bias of each mutation. All types of mutations were observed at the amino acid level, and no difference in the appearance time of all the amino acids was detected (**Figures 2C, D**). Overall, we generated a nonbiased NC08 scFv-CAR saturated mutation pool including more than 1×10^5^ combined mutations.

### Screening and verification of functional mutations

**Figure 3:**
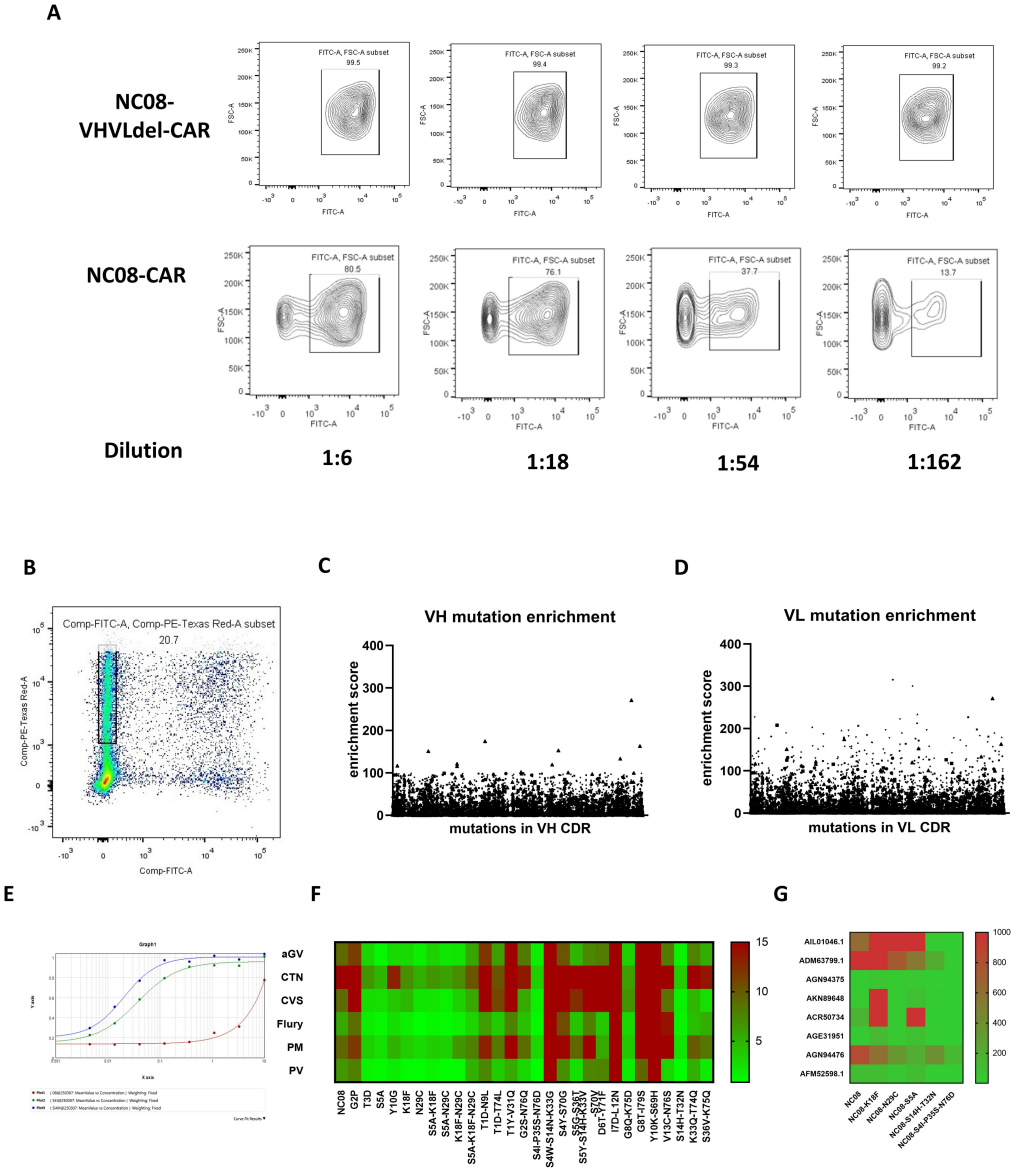
Screening and verification of functional mutations in the VL region. **(A)** Expression of the NC08-CAR blocked the entry of the pseudotyped rabies virus in a dose-dependent manner. **(B)** mCherry-positive GFP-negative cells were isolated for sequencing of mutations that block the entry of pseudotyped rabies virus. **(C)** Enrichment scores of all mutations in isolated cells from cells transfected with VH mutation library. **(D)** Enrichment scores of all mutations in isolated cells from cells transfected with VL mutation library. **(E)** Binding affinity between NC08 or NC08 mutant IgG antibody and rabies pseudotyped virus CVS strain using ELISA. Red line indicates binding curve of NC08 IgG antibody. Green line indicates binding curve of NC08 IgG antibody harboring S4I-P35S-N76D in CDR of VL. Blue line indicates binding curve of NC08 IgG antibody harboring S14H-T32N in CDR of VL. The EC50 of NC08, NC08-VL-S4I-P35S-N76D and NC08-VL- S14H-T32N is >1000ng/ml, 4ng/ml and 2ng/ml, respectively. **(F)** Heatmap of the IC50 values of the NC08 and NC08 mutants against pseudoviruses based on six fixed rabies strains. **(G)** IC50 values of the NC08 and NC08 mutants against pseudotyped viruses based on eight rabies street strains.

High-affinity scFv mutants can be screened on the basis of their ability to block viral cell entry. Accordingly, cells transfected with the NC08 scFv-CAR saturated mutation pool were infected with rabies pseudoviruses for further verification. The cells resistant to infection might harbor scFv mutants with high neutralization ability. Viral infectivity was first tested in cells without the NC08 scFv, but the nonreplicating pseudoviruses had a limited titer and low infection efficiency (data not shown). Therefore, we prepared replication-competent vesicular stomatitis virus (VSV) pseudoviruses packaged in the rabies CVS strain envelope protein. The cells were either transfected with NC08 scFv-CAR variants carrying deletions of VH and VL or with NC08 scFv-CAR plasmids for 24 hours, after which they were infected with pseudoviruses at different dilutions. The infection rate of cells expressing NC08 scFv-CAR variants with VH and VL deletions, which are nonfunctional antibodies, approached 100%, whereas that of cells expressing the NC08 scFv-CAR decreased with increasing virion dilution (**Figure 3A**). Thus, we transfected cells with the NC08 scFv-CAR saturated mutation pool and infected them with 5×10^7^ FFU of CVS-GFP replicative pseudovirus. After 24 hours of culture, the mCherry^+^ GFP^-^ cells were sorted, and the NC08 scFv-CAR mutants effectively prevented viral entry due to increased neutralization ability (**Figure 3B**). The RNA of these cells was extracted, reverse-transcribed and PCR amplified to obtain VH-CDR and VL-CDR mutant sequences for next-generation sequencing. Unsorted cells were used as the control group. Since more than one plasmid may have been transfected into the same cell, the screened cells that were resistant to infection may have expressed additional variants in addition to high-affinity antibodies. To reduce this uncertainty by increasing the statistical signal-to-noise ratio, we repeated the process of transfection, screening and sequencing eight times, resulting in eight biological replicates.

We subsequently evaluated the enrichment of mutations at single amino acid resolution. The screened cells may not only express high-affinity antibodies but also contain other antibody expression plasmids. To minimized false positive rate, we make eight sets of parallel experiments and selected mutants enriched in more than five replicates. The enrichment index was defined as the ratio of the frequency of a certain amino acid in mCherry^+^ GFP^-^ cells to its frequency in the control group (unsorted cells), and an enrichment index greater than two was used as a screening criterion. We found that six individual mutation sites in CDR of VL were enriched in the sorted cells. However, the maximum enrichment index of mutations in CDR of VH was only 1.3, suggesting none of these mutations were significantly enriched. We then synthesized six full-length IgG mAbs that carried the corresponding single-point mutants in CDR of VL, and the neutralization ability of these mAbs against six representative strains of the rabies virus was evaluated. Compared with that of NC08, the neutralizing ability of mAbs with S5A, K18F, or N29C against the six representative strains increased by an average of 4.92, 3.45, and 4.99 times, respectively. On the basis of the neutralization results, mAbs carrying combinations of the S5A, K18F, and N29C mutations were synthesized. However, the combination of these point mutations had no significant effect on the affinity, possibly because the combination of point mutations generated conformational changes in antibodies that led to a decrease in neutralization. To further detect whether combined mutation improved neutralization ability, we calculated the ratio of the frequency of every sequence in the mCherry^+^ GFP^-^ cell population to the frequency in the unsorted cells (**Figure 3C**). From the library of plasmids harboring mutations in CDR of VL, a total of 18 sequences were sorted on the basis of the enrichment ratio, 16 of which carried two mutations, while the other two carried three and four mutations, respectively. However, the enrichment index of mutations in CDR of VH were relatively low and were poorly shared among the replicates. So only enriched mutants in CDR of VL were further analyzed. By constructing and expressing full-length IgG antibodies harboring mutations in these sequences, we found that antibodies carrying the two combined mutations S4I-P35S-N76D and S14H-T32N in VL CDR had a significantly improved binding affinity against rabies virus CVS strain by ELISA. Besides (**Figure 3E**), The EC50 of NC08, NC08-VL-S4I-P35S-N76D and NC08-VL- S14H-T32N is >1000ng/ml, 4ng/ml and 2ng/ml, respectively. Besides, neutralization effect against pseudoviruses of the six representative rabies strains (**Figure 3F** and **Supplementary Table S1**). To further test whether the optimized mAbs have broad-spectrum neutralizing activity against the rabies street strains. We selected 8 rabies street strains from distinct evolutionary branches. NC08 and mutant mAbs with improved neutralizing activity against a fixed rabies strain were used in this assay. The IC50 (ng/ml) of each mAb against each virus is shown in the heatmap (**Figure 3G**; **Supplementary Table S2**). Notably, NC08 exhibited poor neutralizing activity against the AIL01046.1, ADM63799.1 and AGN94476 strains, whereas NC08 carrying the S4I-P35S-N76D or S14H-T32N mutation exhibited significantly improved neutralizing activity against these strains. The IC50 was improved nearly 100-fold.

Overall, we established a high-throughput saturation mutagenesis platform that can be applied to improve antibody affinity. On the basis of this platform, we modified the antirabies NC08 antibody and further investigated the impacts of several single-point and multiple-point mutants on the antibody affinity and neutralization ability.

## Discussion

Commonly used mutagenesis strategies include error-prone PCR, degenerate codon primers, and saturation mutation. Error-prone PCR is a simple method, but it can potentially introduce many redundant mutations, including synonymous mutations, stop codons and reading frame disruptions. This can result in a low-quality mutant library, with effective mutants accounting for only 0.1–0.2% (15). The use of primers with degenerate codons is more efficient than error-prone PCR, but this method still requires further improvement (16). Saturation mutagenesis allows the introduction of 20 amino acid residues per codon to simultaneously evaluate all candidate mutations (17). Therefore, we designed and synthesized two oligonucleotide pools targeting 73 aa in the CHR of VL and VH of NC08 scFv, resulting in saturating mutagenesis for each amino acid sequence, with a coverage of 100% of all expected variants. In addition, mutations can be introduced at multiple sites by adjusting the concentration of the mutagenic primers, further increasing the diversity of the library. Our method achieved saturation mutagenesis through rational design to provide comprehensive variant coverage with unprecedented efficiency.

Several studies on the optimization of antibodies and decoy receptors have used saturation mutagenesis. Compared with those of established methods, the advantages and drawbacks of our workflow can be summarized as follows: 1) Previous studies commonly used degenerate codons to introduce systematic diversity in proteins. The use of the NNK triplet, rather than NNN, enables the encoding of 32 individual codons that cover all 20 amino acids. We used a massively parallel synthesis method on a chip to further reduce the cost of oligonucleotides. 2) Conventional screening is based mainly on the affinity between the purified envelope protein of the virus and mAbs with different mutations. However, some virus envelope proteins need to form polymers to be functional, which may not be possible for some proteins. Furthermore, higher affinity between envelope protein and mAbs does not necessarily means higher neutralization ability. Binding of virus to a suboptimal antibody may enhances its entry into host cells, which had been observed in HIV, RSV and Dengue (18). Here, we substituted the recombinant protein with a pseudovirus coated with the envelope protein of the target virus and evaluated the neutralizing activity of the mAbs by directly assessing their ability to block pseudovirus infection. Despite these advantages, our method also has intrinsic limitations. First, the number of primers used to generate the mutant library was limited and required 2-step PCR. This process may introduce mutation bias in the library. A balance between generating enough primers and not inducing mutation bias needs to be achieved by optimizing the number of PCR cycles.

The ability to protect against viral infection is one of the main evaluation criteria for high-affinity scFv mutants. We thus transfected cells with the NC08 scFv-CAR saturated mutation pool, after which we infected them with rabies pseudoviruses. The cells resistant to infection were candidates harboring scFv mutants with high neutralization ability. Notably, high viral infection efficiency in cells transfected with a vector expressing a nonfunctional antibody is a prerequisite of this screening method. Conversely, a low infection rate would make it impossible to determine whether heterologous single-chain antibodies play a role in preventing already improbable infections.

We screened high-affinity mutant antibodies on the basis of the ability of neutralizing antibodies to prevent pseudoviruses from entering cells. Pseudoviruses are widely used in the study of highly pathogenic viruses because they are able to express and display glycoproteins of the authentic virus on the pseudovirus envelope (19, 20). Although numerous pseudovirus display systems are based on lentiviral vectors, cells are infected at low MOIs ​​to ensure that only one mutant is expressed in a single cell (21). To improve the screening process and reduce the screening duration, we constructed a mutant library using a eukaryotic expression plasmid and directly transfected the cells with the bulk library DNA. Although the screened cells may not only express high-affinity antibodies but also contain other antibody expression plasmids, we used multiple sets of parallel experiments to select mutant strains with a high enrichment index in most experiments to avoid bias potentially introduced during transfection, screening, and sequencing. Notably, we identified several mutations, including S5A, K18F, and N29C combined mutations S4I-P35S-N76D and S14H-T32N, which could improve antibody neutralization ability. Among them, N29C introduce an additional cysteine residue in the CDR. It had been reported that unpaired cysteine tends to be cysteinylation and leading to negative effect on antibody stability and activity (22–24). So this mutation should be avoided in manufacture process.

In summary, we established a high-throughput saturation mutagenesis platform that can be applied for affinity modification of antibodies against most viruses. On this platform, we modified the antirabies NC08 antibody and obtained several single-point and multipoint mutants with enhanced antiinfection ability.

## Data Availability

The original contributions presented in the study are included in the article and supplementary material, further inquiries can be directed to the corresponding authors.

## Supplementary material

Supplementary Figure 1 Original western blot images related to **Figure 1B**.

Supplementary Table 1 IC50(ng/ml) of NC08 and NC08 mutants against six rabies fixed strain pseudotyped virus.

Supplementary Table 2 IC50(ng/ml) of NC08 and NC08 mutants against eight rabies street strain pseudotyped virus.
