# Supplementary material for: Establishment of rapid saturation mutagenesis and screening methods for improving the neutralizing activity of monoclonal antibodies
Source: Front Immunol. 2025 Nov 27;16:1722831. doi: 10.3389/fimmu.2025.1722831 (PMC12695841; doi:10.3389/fimmu.2025.1722831)
Supplement: Supplementary Figure 1 — Original western blot images related to Figure 1B. [file Table1.docx]

|  | AIL01046.1 | ADM63799.1 | AGN94375 | AKN89648 | ACR50734 | AGE31951 | AGN94476 | AFM52598.1 |
| --- | --- | --- | --- | --- | --- | --- | --- | --- |
| NC08 | 631.6493 | >1000 | 19.60916 | 26.36 | 159.89 | 20.13 | 831.7052 | 100 |
| NC08-K18F | >1000 | >1000 | 52.13125** | >1000 | >1000 | 122.1793 | 598.6224 | 100 |
| NC08-N29C | >1000 | 731.86 | 15.48714 | 24.0269** | 17.08** | 32.41584 | 348.8868 | 35.08257 |
| NC08-S5A | >1000 | 537.1532 | 31.21454 | 89.86924 | >1000 | 80.26384 | 195.062* | 100 |
| NC08-S14H-T32N | 18.00582** | 215.5317* | 1.969017** | 10.67549 | 3.04*** | 4.34707** | 389.2312 | 6.331623** |
| NC08-S4I-P35S-N76D | 7.293734*** | 7.520208*** | 1.578967** | 2.001765** | 1.45*** | 2.612503** | 133.1544* | 3.197638** |

Table1. IC50(ng/ml) of NC08 and NC08 mutants against six rabies fixed strain pseudotyped virus

|  | aGV | CTN | CVS | Flury | PM | PV |
| --- | --- | --- | --- | --- | --- | --- |
| NC08 | 9.15 | 23.27 | 9.02 | 5.61 | 9.27 | 6.65 |
| G2P | 11.57 | 102.43 | 17.95 | 8.79 | 11.71 | 7.88 |
| T3D | 3.15 | 6.69* | 2.73 | 1.76* | 3.48 | 1.47** |
| S5A | 1.23 | 4.96** | 2.59 | 1.21* | 2.54 | 1.18** |
| Y10G | 2.86 | 13.14 | 3.95 | 2.55* | 5.03 | 1.81* |
| K18F | 2.92 | 7.06* | 4.77 | 1.7* | 3.61 | 1.02** |
| N29C | 1.7 | 5.19* | 2.25 | 0.85* | 2.87 | 1.06** |
| S5A-K18F | 3.32 | 6.63* | 2.28 | 1.34* | 3.54 | 1.49** |
| S5A-N29C | 2.16 | 5.97* | 1.64 | 1.09* | 3.35 | 0.96** |
| K18F-N29C | 2.24 | 7.15* | 3.36 | 1.67* | 6.57 | 1.09** |
| S5A-K18F-N29C | 5.74 | 10.3* | 5.67 | 3.19* | 7.85 | 5.12 |
| T1D-N9L | 12.04 | 27.43 | 16.85 | 6.26 | 13.12 | 7.42 |
| T1D-T74L | 6.28 | 10.3* | 11.1 | 3.64 | 8.29 | 3.68 |
| T1Y-V31Q | 16.54 | 38.67 | 20.73 | 7.8 | 23.79 | 5.53 |
| G2S-N76Q | 8.18 | 13.69* | 5.56 | 4.32 | 8.94 | 3.04 |
| S4I-P35S-N76D | 1.75 | 3.42** | 1.62 | 1.22* | 3.03 | 0.96** |
| S4W-S14N-K33G | 54.8 | 227.94 | 41.43 | 39.13 | 35.7 | 26.41 |
| S4Y-S70G | 10.32 | 35.18 | 15.45 | 10.82 | 14.81 | 7 |
| S5G-S36T | 5 | 26.46 | 12.48 | 4.1 | 9.18 | 2.54 |
| S5Y-S14H-K33V-S70V | 7.49 | 46.05 | 27.28 | 7.91 | 16.03 | 7.63 |
| D6T-Y71F | 8.01 | 20.9 | 14.89 | 3.87 | 5.63 | 7.73 |
| I7D-L12N | >1000# | >1000 | >1000 | 952.38 | 594.25 | >1000 |
| G8Q-K75D | 3.39 | 6.19** | 6.79 | 2.96 | 3.85 | 1.8* |
| G8T-I79S | 16.66 | 68.45 | 48.65 | 22.03 | 18.65 | 9.35 |
| Y10K-S69H | 254.44 | >1000 | >1000 | 555.56 | 222.36 | 233.5 |
| V13C-N76S | 8.13 | 15.74 | 8.72 | 14.05 | 26.83 | 3.08 |
| S14H-T32N | 1.56 | 3.6* | 1.52 | 1.47* | 2.33* | 0.87** |
| K33Q-T74Q | 7.76 | 22.89 | 4.8 | 4.36 | 9.97 | 5.24 |
| S36V-K75Q | 5.32 | 13.82 | 2.61 | 5.31 | 8.35 | 1.87* |

# >1000 indicated no neutralizing activity; * indicated p<0.05 ** indicated p<0.01

Table2. IC50(ng/ml) of NC08 and NC08 mutants against eight rabies street strain pseudotyped virus

# >1000 indicated no neutralizing activity * indicated p<0.05 ** indicated p<0.01 *** indicated p<0.001
