# Supplementary material for: Establishment of rapid saturation mutagenesis and screening methods for improving the neutralizing activity of monoclonal antibodies
Source: Front Immunol. 2025 Nov 27;16:1722831. doi: 10.3389/fimmu.2025.1722831 (PMC12695841; doi:10.3389/fimmu.2025.1722831)
Supplement: Supplementary Table 1 — IC50(ng/ml) of NC08 and NC08 mutants against six rabies fixed strain pseudotyped virus. [file Table2.docx]

Supplemental Figure1


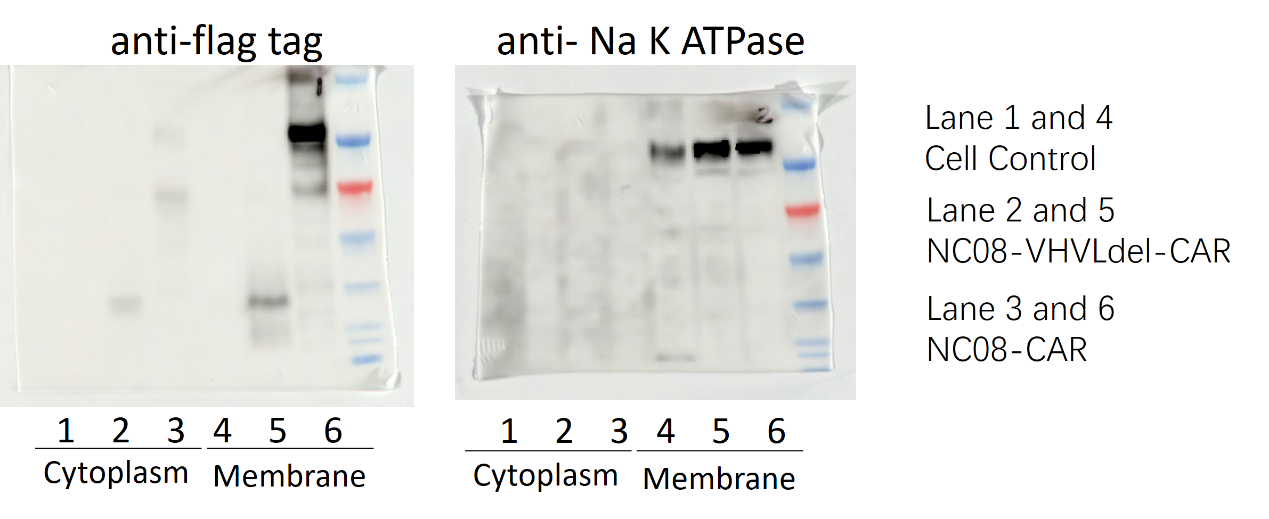


**Supplemental Figure1: Original western blot images related to Figure1B**

Plasmids expressing NC08-CAR (lanes 3 and 6) or NC08-VHVLdel-CAR (lanes 2 and 4) were used to transfect 293T cells. Cells transfected with Cytoplasmic and membrane protein fractions were collected, and the localization of the NC08-CAR was confirmed by western blotting with an anti-FLAG monoclonal antibody (Left). The separation of the cytoplasm and the membrane was confirmed by the membrane marker Na, K ATPase.(Right)
