# Supplementary material for: Establishment of rapid saturation mutagenesis and screening methods for improving the neutralizing activity of monoclonal antibodies
Source: Front Immunol. 2025 Nov 27;16:1722831. doi: 10.3389/fimmu.2025.1722831 (PMC12695841; doi:10.3389/fimmu.2025.1722831)
Supplement: Supplementary Table 2 — IC50(ng/ml) of NC08 and NC08 mutants against eight rabies street strain pseudotyped virus. [file Table3.docx]

This is a detailed protocol for establishing high-throughput saturation-mutagenesis pool of scFv antibody and screening for mutations that improve neutralization ability of the antibody.

Page 1 to Page 4: Experimental methods.

Page 5 to Page 7: Sequence and annotation of NC08-CAR

Page 8 to Page 49: Oligo library used in this work.

**Experiment methods**

1. **Construction of scFv-CAR expressing vector**

The variable region of the light chain (VL) and variable region of the heavy chain (VH) was obtained for a Chinese patent (CN 101812131 B). To express and display the NC08 antibody on the cell surface, The VH and VL domains of NC08 were joined into a single-chain fragment variant (ScFv) via a flexible linker. A signal peptide was added to the N-terminus of the mAb, followed by a 3×Flag tag and the transmembrane domain adopted from the structure of CAR-T cells was fused to the C-terminal. Then, mCherry was fused with the T2A sequence to the C-terminal. Sequence and annotation of NC08-CAR was shown on Page 5 to Page 7. To maximize translational efficiency, a Kozak sequence was inserted directly upstream of the start codon. This expression cassette was synthesized by General Biosystems (Anhui, China) and subsequently cloned into pcDNA3.1(+) at enzyme sites XhoI and BamHI.

1. **Primer-Pool Synthesis**
   Complementarity-determining regions (CDR) of VH and VL was obtained from a Chinese patent (CN 101812131 B). The VH-CDR and VL-CDR contain 40 and 32 amino-acid positions, respectively. Oligos were designed to mutate each position to the other 19 naturally occurring amino acids. Taking the NC08 DNA sequence as the template, we designed mutagenic oligos in which the target codon is changed to the human-preferred codon for each of the 19 alternative amino acids while keeping 21 nt unchanged on both the 5′ and 3′ sides of the codon. Every oligo therefore carries the mutant codon flanked by 21 nt of wild-type sequence. The 5’ and 3’ sides of the oligo were flanked by “5’-GCTCTTCA-3’ ” and “5’-TGAAGAGC-3’ ” which are BspQI recognition site, respectively. Followed outwardly by a universal adapter for PCR amplification as “5’-CCAATACGGATGACGGG-3’ ” on 5’ side and “5’- CCTCTGTTATTGCCGCC- 3’ ” on 3’ side. Design of oligo was shown as below:


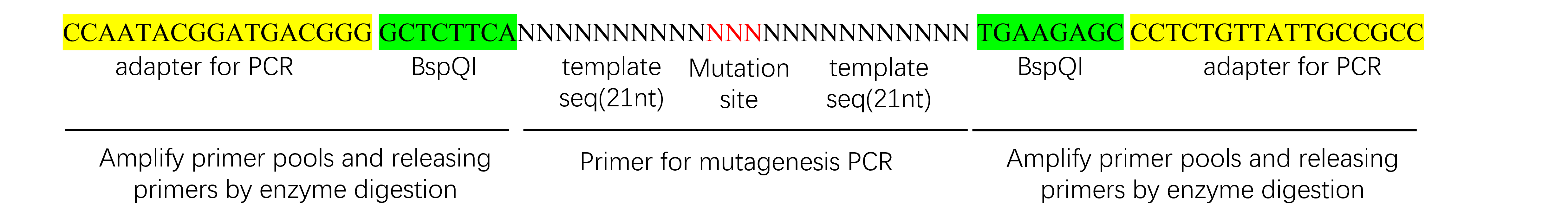


In total, 760 VH-CDR and 608 VL-CDR mutagenic oligos were synthesized.

1. **Construction of the mutant plasmid library**
   To amplify the oligo pool, PCR was performed using forward primer “5’-CCAATACGGATGACGGG-3’ ” and reverse primer “5’- GGCGGCAATAACAGAGG-3’ ”. Reaction mix (50 µL): 10ng oligo pool, 2 µL each of 10 µM adapter primers, 25 µL 2×PrimeSTAR Mix (TaKaRa, Japan), and ddH_2_O to 50 µL. Cycling on a Mastercycler (Eppendorf, Hamburg, Germany): 95 °C 2 min; 10 cycles of 95 °C 20 s, 53 °C 20 s, 72 °C 30 s; final extension 72 °C 2 min. The ~95-bp products were excised from a 2 % agarose gel and purified with QIAquick Gel Extraction Kit (QIAGEN, Hilden, Germany; cat. 28704) and dissolved in 30µL ddH_2_O. The product was digested by BspQI(New England Biolabs, USA): 2x rCutSmart 5µL, purified PCR product 30µL, BspQI 1µL, add ddH_2_O to 50µL, 37 °C 2-4hr. After BspQI digestion, a second gel purification (QIAGEN cat. 20021) was performed, ~45-bp double-stranded mutagenic fragments were obtained. The 45-bp primer pool was then used for generate saturation-mutagenesis on scFv-CAR. scFv-CAR expressing vector was used as template. Reaction (20 µL): 1 µL primer pool, 1 µL plasmid, 10 µL 2×PrimeSTAR Mix, 8 µL ddH_2_O. Cycling: 95 °C 5 min; 30 cycles of 95 °C 30 s, 55 °C 30 s, 72 °C 8 min; final extension 72 °C 10 min. The PCR product was digested with DpnI to eliminate methylated parental DNA: 16 µL PCR product + 2 µL 10× rCutSmart Buffer + 2 µL DpnI (New England Biolabs), 37 °C 6 h. The digested mixture was transformed into chemically competent DH5α cells, plated on LB-ampicillin, and incubated overnight at 37 °C. Ten random clones were picked, mini-prepped, and sequenced with CMV-Forward and BGH-R primers. Alignment to the reference showed ~75 % of the target positions carried the intended mutation. Mutation density could be modulated by adjusting the PCR conditions. After optimization, large-scale library construction was carried out: mutagenized products were transformed onto 40–60 LB plates, all colonies were pooled, and plasmid DNA was extracted with the Qiagen Maxi Kit. The resulting VH-CDR and VL-CDR mutant plasmid libraries were sent to Beijing Genomics Institute (Novogene) for next-generation sequencing to assess mutation distribution, substitution pattern, and library uniformity.
2. **Transfection and screening for high affinity scFv-CAR**

293T cells were cultured, dissociated and resuspended at 4×10⁵ cells/mL. 5ml of the suspension were seeded into Corning T25 flasks and cultured for 24 h. When confluence reached ~70–80 %, transfection was performed with Lipofectamine 3000 (Thermo Fisher Scientific) according to the manufacturer’s instructions. Each flask was transfected with 30 µg of the VH-CDR or VL-CDR mutant-library plasmids.24hrs post-transfection, mCherry reporter expression was examined under an inverted fluorescence microscope and quantified by flow cytometry (FACS). Untransfected cells and cells transfected with mCherry expression plasmid were used to set the gates.

For pseudovirus challenge, virus with GFP reporter was added and incubated for 4–6 hr, after which the supernatant was removed and replaced with fresh complete medium for a further 18–20 hr. Cells were harvested at 24 h post-infection (hpi), prepared as single-cell suspensions and passed through 70 µm cell-strainers. Sorting was performed on a FACS Aria with the following gating strategy:

1. Debris exclusion by FSC/SSC.

Dual-colour mCherry vs. GFP plot: FMO (fluorescence-minus-one) and uninfected blanks were used to set the mCherry and GFP positive gates so that the “false-positive” rate in each negative population was ≤1 %.

1. Target population: The unsorted starting population (P0) was retained as the baseline control. Post-sort purity was re-checked; only populations with ≥95 % mCherry⁺/GFP⁻ cells were processed further. mCherry⁺/GFP⁻ cells (antibody-expressing, uninfected) was sorted, and 5×10⁵ cells were collected.
2. **Amplification of VL and VH CDR**

Total RNA was extracted from sorted cells using the RNeasy Mini Kit (Qiagen, cat. 74106) following the manufacturer’s protocol. Two-step reverse transcription was carried out with SuperScript™ III Reverse Transcriptase (Invitrogen, Thermo Fisher Scientific):

1. Mix 1 (10 µL): random primer (20 µM, 1 µL), dNTP mix (10 mM, 1 µL), total RNA 8 µL; 65 °C 5 min, chilled on ice 1 min.
2. Mix 2 (10 µL): 10× RT buffer 2 µL, 25 mM MgCl₂ 4 µL, 0.1 M DTT 2 µL, RNaseOUT 1 µL, SuperScript III 1 µL.
3. Mix 1 was combined with Mix 2 (total 20 µL) and incubated at 50 °C 60 min, 55 °C 60 min, 85 °C 5 min, then held at 4 °C. 1µL of RNase H was added and incubated at 37 °C for 20 min to remove RNA–DNA hybrids.

CDR amplification: the resulting cDNA was used as template for PCR amplification of the integrated CDR fragments. For amplification of VL CDR, forward Each 50 µL reaction contained 1 µL each of forward and reverse primers, 1 µL cDNA, 25 µL 2×PrimeSTAR mix and 22 µL ddH₂O. Cycling conditions: 95 °C 5 min; 35 cycles of 95 °C 30 s, 50 °C 30 s, 72 °C 3 min; final extension 72 °C 10 min; hold at 4 °C. Products were resolved on 1.5 % agarose gels until target bands were clearly separated from background. Gel slices were excised and purified with the QIAquick Gel Extraction Kit (Qiagen) and eluted in 50 µL ddH₂O (yield ≥100 ng). Samples were sent to Beijing Genomics Institute (Novogene) for Illumina paired-end high-throughput sequencing and 5Gb data was obtained per sample. Raw Sequencing Data was processed by Cutadapt to 1) Remove primer and adapter sequences; 2) Trim bases with Phred quality < 20 from both ends; 3) Discard reads in which >10 % of bases are N. Then, clean reads were subsequently merged with Pandaseq. The target sequence was translated into amino acids sequence.

1. **Selection of enriched mutations.**

The amino acid sequence with predicted length were subjected to further analysis. Enrichment of mutations were calculated using two methods:

Method 1:

1. Each of the sequences was aligned with reference sequence. AA changes at each position were recorded. The frequency of a mutation was calculated as the ratio of the count of such mutation to total number of the sequences.
2. The enrichment index was defined as the ratio of the frequency of a certain amino acid in mCherry^+^ GFP^-^ cells to its frequency in the control group (unsorted cells), and an enrichment index greater than two was used as a screening criterion.

Method 2:

1. The count of each sequence was recoded. The frequency of a sequence was calculated as the ratio of the count of such sequence to total number of the sequences.
2. The enrichment index was defined as ratio of the frequency of every sequence in the mCherry^+^ GFP^-^ cell population to the frequency in the unsorted cells. Top 100 enriched sequences from eight duplicates were selected. To minimized false positive rate, we selected mutants enriched in more than five replicates for further confirmation.
3. **Confirmation of antibody potency by pseudotyped virus neutralization assay**

To verify the function of selected mutations, we used full-length IgG monoclonal antibodies harbouring indicated mutations prepared by Sinobiological company. The neutralization assay and analysis were performed as follows:


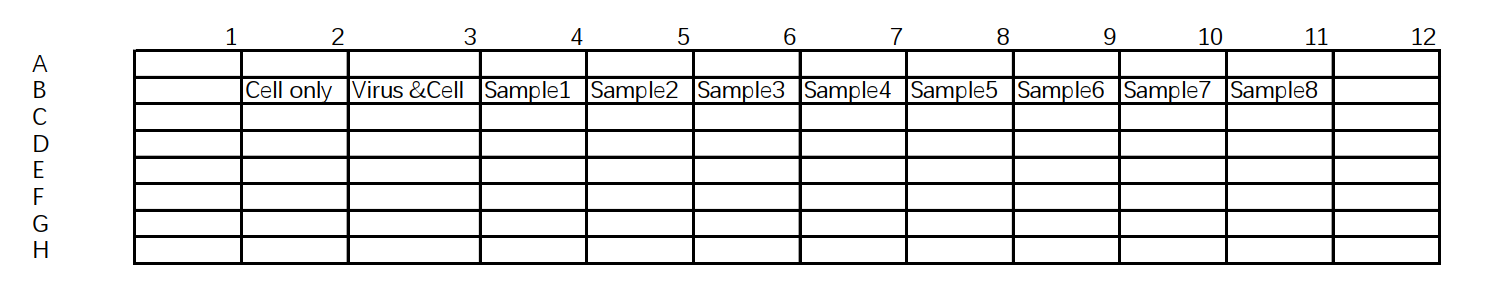


(1) **Sample preparation**: adjust the monoclonal antibody (mAb) concentration to 1,000 ng/mL.
(2) **Peripheral-well filling**: add 100 µL of autoclaved water to each of the 36 outer wells of the 96-well plate to minimize edge effects.
(3) **Starting dilution series**: Add 125 µL of the 1,000 ng/mL mAb into wells B4–B11 and perform five 5-fold serial dilutions.
(4) **Serial dilution**: Gently pipette the contents of wells B4–B11 up and down 6–8 times, then transfer 25 µL to the corresponding wells C4–C11. Repeat this 5-fold dilution scheme row-wise until row G; after mixing row G discard 25 µL.
(5) **Virus addition**: Tetrieve pseudovirus aliquots from –80 °C, dilute to the desired titre in high-glucose DMEM complete medium, and add 50 µL per well to columns 3–11.
(6) **Neutralization**: Incubate the plate for 1 h at 37 °C, 5 % CO₂.
(7) **Cell preparation**: During step 6, dissociate 293T cells and adjust to 4 × 10⁵ cells/mL.
(8) **Cell seeding**: After the 1-h incubation, add 100 µL of the cell suspension per well (4 × 10⁴ cells/well).
(9) **Culture**: Incubate the plate for 24 h at 37 °C, 5 % CO₂ (see Figure 2.2).
(10) **Luciferase assay**: At 24 h post-infection remove 150 µL of supernatant, leaving 100 µL per well. Add 100 µL of substrate, incubate for 2 min at room temperature in the dark, mix thoroughly, and transfer 150 µL of the mixture to a white assay plate.
(11) **RLU reading**: quantify relative light units (RLU) on a PerkinElmer EnSight multimode imager.
(12) **ID₅₀ calculation**: express inhibition relative to the mean RLU of virus-only controls and determine the 50 % inhibitory dose (ID₅₀) by the Reed–Muench formula:
ID₅₀ = 10^{lg(X) + lg(1/K) × (0.5 – B)/(A – B)}
where
A = inhibition rate of the well above 50 % fluorescence
B = inhibition rate of the well below 50 % fluorescence
X = dilution factor corresponding to the well below 50 % inhibition
K = dilution factor (5-fold).

IC_50_ = antibody concentration/ID_50_

To make ID50 comparable among different antibodies, all the antibodies were evaluated in the same lot. And experiments were repeated.

**Sequence and annotation of NC08-CAR**

LOCUS chimeric_CD28P-3 2997 bp DNA linear UNA 28-JAN-2023

DEFINITION natural linear DNA

ACCESSION .

VERSION .

KEYWORDS .

SOURCE natural DNA sequence

ORGANISM unspecified

REFERENCE 1 (bases 1 to 2997)

AUTHORS .

TITLE Direct Submission

JOURNAL Exported Jan 30, 2023 from SnapGene Viewer 6.2.0

https://www.snapgene.com

FEATURES Location/Qualifiers

source 1..2997

/mol_type="genomic DNA"

/organism="unspecified"

CDS 7..69

/codon_start=1

/label=signal peptide

/translation="MLRLLLALNLFPSIQVTGGSS"

CDS 70..135

/codon_start=1

/product="three tandem FLAG(R) epitope tags, followed by an

enterokinase cleavage site"

/label=3xFLAG

/translation="DYKDHDGDYKDHDIDYKDDDDK"

CDS 142..783

/label=VL

misc_feature 199..240

/label=CDR1

misc_feature 286..306

/label=CDR2

misc_feature 403..435

/label=CDR3

CDS 784..828

/codon_start=1

/label=linker

/translation="GGGGSGGGGSGGGGS"

CDS 829..1497

/codon_start=1

/label=VH

/translation="LESGPGLVKPSETLSLTCTVSGGSISSVNSYWGWIRQPPGKGLEW

IGNFYYSGNTHYNPSLKSRVTISVGTSKNQFSLKLNSVTAADTAVYYCARQSTIGGFFD

YWGQGTLVTVSSASTKGPSVFPLAPSSKSTSGGTAALGCLVKDYFPEPVTVSWNSGALT

SGVHTFPAVLQSSGLYSLSSVVTVPSSSLGTQTYICNVNHKPSNTKVDKKVEPKSCDKT

S"

misc_feature 892..927

/label=CDR1

misc_feature 970..1017

/label=CDR2

misc_feature 1108..1143

/label=CDR3

CDS 1504..1638

/codon_start=1

/label=CD8a

/translation="TTTPAPRPPTPAPTIASQPLSLRPEACRPAAGGAVHTRGLDFACD

"

CDS 1648..1743

/codon_start=1

/label=CD28TM

/translation="PSKPFWVLVVVGGVLACYSLLVTVAFIIFWVR"

CDS 1744..1869

/label=41BB

CDS 1876..2217

/codon_start=1

/label=CD3z

/translation="LRVKFSRSADAPAYQQGQNQLYNELNLGRREEYDVLDKRRGRDPE

MGGKPQRRKNPQEGLYNELQKDKMAEAYSEIGMKGERRRGKGHDGLYQGLSTATKDTYD

ALHMQALPPR"

CDS 2224..2280

/codon_start=1

/product="2A peptide from porcine teschovirus-1

polyprotein"

/label=P2A

/note="Eukaryotic ribosomes fail to insert a peptide bond

between the Gly and Pro residues, yielding separate

polypeptides."

/translation="ATNFSLLKQAGDVEENPGP"

CDS 2281..2991

/codon_start=1

/label=mcherry

/translation="MVSKGEEDNMAIIKEFMRFKVHMEGSVNGHEFEIEGEGEGRPYEG

TQTAKLKVTKGGPLPFAWDILSPQFMYGSKAYVKHPADIPDYLKLSFPEGFKWERVMNF

EDGGVVTVTQDSSLQDGEFIYKVKLRGTNFPSDGPVMQKKTMGWEASSERMYPEDGALK

GEIKQRLKLKDGGHYDAEVKTTYKAKKPVQLPGAYNVNIKLDITSHNEDYTIVEQYERA

EGRHSTGGMDELYK"

ORIGIN

1 gccaccatgc tcaggctgct cttggctctc aacttattcc cttcaattca agtaacagga

61 gggtcttcgg actacaagga tcatgacgga gactataagg atcacgatat tgattacaaa

121 gatgacgacg acaaagttaa cgagctccag cctgcctccg tgtctgggtc tcttggacag

181 tcgatcacca tctcctgcac tggaaccagc agtgatattg ggaattataa ccttgtctcc

241 tggtaccaac aacacccagg caaagccccc aaactcataa tttatgaggt cactaagcgg

301 ccctcagggg tttctaatcg cttctctggc tccaagtctg gcaacacggc ctccctgacc

361 atctctgggc tccaggctga ggacgaggct aattactact gcagctcata tacagccacc

421 aagaattact ggattttcgg cggagggacc aagctgaccg tccgaggtca gcccaaggct

481 gccccctcgg tcactctgtt cccgccctcc tctgaggagc ttcaagccaa caaggccaca

541 ctggtgtgtc tcataagtga cttctacccg ggagccgtga cagtggcctg gaaggcagat

601 agcagccccg tcaaggcggg agtggagacc accacaccct ccaaacaaag caacaacaag

661 tacgcggcca gcagctatct gagcctgacg cctgagcagt ggaagtccca cagaagctac

721 agctgccagg tcacgcatga agggagcacc gtggagaaga cagtggcccc tacagaatgt

781 tcaggcggcg gcggcagcgg cggcggcggc agcggcggcg gcggcagcct cgagtcgggc

841 ccaggactgg tgaagccttc ggagaccctg tccctcacct gcactgtctc tggtggctcc

901 atcagcagtg ttaattccta ctggggctgg atccgccagc ccccagggaa ggggctggag

961 tggattggga atttctatta tagtgggaac acccactaca acccgtccct caagagtcga

1021 gtcaccatat ccgtaggcac gtccaagaac cagttctccc tgaagctgaa ctctgtgacc

1081 gccgcagaca cggctgtata ttactgtgcg agacagtcga ccataggggg cttctttgac

1141 tactggggcc agggaaccct ggtcaccgtc tcctcagcct ccaccaaggg cccatcggtc

1201 ttccccctgg caccctcctc caagagcacc tctgggggca cagcggccct gggctgcctg

1261 gtcaaggact acttccccga accggtgaca gtgtcgtgga attccggcgc cctgaccagc

1321 ggcgtgcaca cattccctgc cgtgctgcag agctccggcc tgtacagcct gagcagcgtg

1381 gtgacagtgc ccagcagctc cctgggcaca cagacataca tctgcaacgt gaaccacaag

1441 cccagcaata caaaggtgga caagaaggtg gagcctaagt cctgcgacaa gaccagcgtg

1501 aacaccacca caccagcccc cagacctccc acccccgctc ctacaatcgc ctcccagcct

1561 ctgtccctga ggcctgaggc ctgtaggcct gccgccggag gagctgtgca caccagaggc

1621 ctggacttcg cctgcgatag aaggcctccc tccaagccct tttgggtgct ggtggtggtg

1681 ggcggcgtgc tggcttgcta ctccctgctg gtgacagtgg cttttatcat cttctgggtg

1741 aggaagagag gcaggaagaa gctgctgtac atctttaagc agccctttat gagacctgtg

1801 cagaccaccc aggaggagga cggctgcagc tgcagattcc ctgaggagga ggagggcggc

1861 tgtgagctgg ccagcctgag ggtgaagttc agcaggtccg ccgacgcccc tgcctaccag

1921 cagggacaga accagctgta caacgagctg aatctgggca ggagagagga gtacgatgtg

1981 ctggacaaga gaaggggcag ggaccctgag atgggcggca agcctcagag aagaaagaac

2041 ccccaggagg gcctgtacaa cgaactgcag aaggataaga tggccgaggc ctacagcgag

2101 atcggcatga agggcgagag aaggaggggc aagggccacg atggcctgta ccagggcctg

2161 agcaccgcca caaaggatac ctacgatgcc ctgcacatgc aggccctgcc ccccagagcc

2221 agcgctacaa acttctccct gctgaagcag gccggcgatg tggaggagaa ccccggccct

2281 atggtgtcca agggcgagga ggacaatatg gccatcatca aggagttcat gagattcaaa

2341 gtgcacatgg agggctccgt gaacggccac gagttcgaga tcgagggcga gggcgaggga

2401 agaccttacg agggcacaca gaccgccaag ctgaaggtga ccaagggcgg ccctctgccc

2461 tttgcctggg acatcctgag ccctcagttc atgtacggca gcaaggccta cgtgaagcac

2521 cccgccgaca tccctgatta cctgaagctg agcttccctg agggcttcaa gtgggagaga

2581 gtgatgaatt tcgaggacgg cggcgtggtg accgtgaccc aggattccag cctgcaggac

2641 ggcgagttta tctacaaggt gaagctgagg ggcacaaatt tccctagcga tggccctgtg

2701 atgcagaaga agaccatggg ctgggaggcc agctccgaga gaatgtaccc cgaggacggc

2761 gccctgaagg gcgagatcaa gcagaggctg aagctgaagg atggcggcca ctacgacgcc

2821 gaggtgaaga ccacatacaa ggccaagaag cctgtgcagc tgcctggcgc ctacaacgtg

2881 aatatcaagc tggatatcac atcccacaac gaggactaca caatcgtgga gcagtacgag

2941 agagccgagg gcaggcactc caccggcgga atggatgagc tgtacaagtg actcgag

//

| **Oligo library:**  VLCHR1_1Gly | CCAATACGGATGACGGGGCTCTTCACAGTCGATCACCATCTCCTGCGGGGGAACCAGCAGTGATATTGGGTGAAGAGCCCTCTGTTATTGCCGCC |
| --- | --- |
| VLCHR1_1Ala | CCAATACGGATGACGGGGCTCTTCACAGTCGATCACCATCTCCTGCGCTGGAACCAGCAGTGATATTGGGTGAAGAGCCCTCTGTTATTGCCGCC |
| VLCHR1_1Val | CCAATACGGATGACGGGGCTCTTCACAGTCGATCACCATCTCCTGCGTGGGAACCAGCAGTGATATTGGGTGAAGAGCCCTCTGTTATTGCCGCC |
| VLCHR1_1Leu | CCAATACGGATGACGGGGCTCTTCACAGTCGATCACCATCTCCTGCCTAGGAACCAGCAGTGATATTGGGTGAAGAGCCCTCTGTTATTGCCGCC |
| VLCHR1_1Ile | CCAATACGGATGACGGGGCTCTTCACAGTCGATCACCATCTCCTGCATCGGAACCAGCAGTGATATTGGGTGAAGAGCCCTCTGTTATTGCCGCC |
| VLCHR1_1Pro | CCAATACGGATGACGGGGCTCTTCACAGTCGATCACCATCTCCTGCCCAGGAACCAGCAGTGATATTGGGTGAAGAGCCCTCTGTTATTGCCGCC |
| VLCHR1_1Phe | CCAATACGGATGACGGGGCTCTTCACAGTCGATCACCATCTCCTGCTTTGGAACCAGCAGTGATATTGGGTGAAGAGCCCTCTGTTATTGCCGCC |
| VLCHR1_1Tyr | CCAATACGGATGACGGGGCTCTTCACAGTCGATCACCATCTCCTGCTACGGAACCAGCAGTGATATTGGGTGAAGAGCCCTCTGTTATTGCCGCC |
| VLCHR1_1Trp | CCAATACGGATGACGGGGCTCTTCACAGTCGATCACCATCTCCTGCTGGGGAACCAGCAGTGATATTGGGTGAAGAGCCCTCTGTTATTGCCGCC |
| VLCHR1_1Ser | CCAATACGGATGACGGGGCTCTTCACAGTCGATCACCATCTCCTGCTCAGGAACCAGCAGTGATATTGGGTGAAGAGCCCTCTGTTATTGCCGCC |
| VLCHR1_1Cys | CCAATACGGATGACGGGGCTCTTCACAGTCGATCACCATCTCCTGCTGCGGAACCAGCAGTGATATTGGGTGAAGAGCCCTCTGTTATTGCCGCC |
| VLCHR1_1Met | CCAATACGGATGACGGGGCTCTTCACAGTCGATCACCATCTCCTGCATGGGAACCAGCAGTGATATTGGGTGAAGAGCCCTCTGTTATTGCCGCC |
| VLCHR1_1Asn | CCAATACGGATGACGGGGCTCTTCACAGTCGATCACCATCTCCTGCAATGGAACCAGCAGTGATATTGGGTGAAGAGCCCTCTGTTATTGCCGCC |
| VLCHR1_1Gln | CCAATACGGATGACGGGGCTCTTCACAGTCGATCACCATCTCCTGCCAGGGAACCAGCAGTGATATTGGGTGAAGAGCCCTCTGTTATTGCCGCC |
| VLCHR1_1Asp | CCAATACGGATGACGGGGCTCTTCACAGTCGATCACCATCTCCTGCGACGGAACCAGCAGTGATATTGGGTGAAGAGCCCTCTGTTATTGCCGCC |
| VLCHR1_1Glu | CCAATACGGATGACGGGGCTCTTCACAGTCGATCACCATCTCCTGCGAAGGAACCAGCAGTGATATTGGGTGAAGAGCCCTCTGTTATTGCCGCC |
| VLCHR1_1Lys | CCAATACGGATGACGGGGCTCTTCACAGTCGATCACCATCTCCTGCAAGGGAACCAGCAGTGATATTGGGTGAAGAGCCCTCTGTTATTGCCGCC |
| VLCHR1_1Arg | CCAATACGGATGACGGGGCTCTTCACAGTCGATCACCATCTCCTGCCGCGGAACCAGCAGTGATATTGGGTGAAGAGCCCTCTGTTATTGCCGCC |
| VLCHR1_1His | CCAATACGGATGACGGGGCTCTTCACAGTCGATCACCATCTCCTGCCATGGAACCAGCAGTGATATTGGGTGAAGAGCCCTCTGTTATTGCCGCC |
| VLCHR1_2Ala | CCAATACGGATGACGGGGCTCTTCATCGATCACCATCTCCTGCACTGCTACCAGCAGTGATATTGGGAATTGAAGAGCCCTCTGTTATTGCCGCC |
| VLCHR1_2Val | CCAATACGGATGACGGGGCTCTTCATCGATCACCATCTCCTGCACTGTTACCAGCAGTGATATTGGGAATTGAAGAGCCCTCTGTTATTGCCGCC |
| VLCHR1_2Leu | CCAATACGGATGACGGGGCTCTTCATCGATCACCATCTCCTGCACTCTTACCAGCAGTGATATTGGGAATTGAAGAGCCCTCTGTTATTGCCGCC |
| VLCHR1_2Ile | CCAATACGGATGACGGGGCTCTTCATCGATCACCATCTCCTGCACTATAACCAGCAGTGATATTGGGAATTGAAGAGCCCTCTGTTATTGCCGCC |
| VLCHR1_2Pro | CCAATACGGATGACGGGGCTCTTCATCGATCACCATCTCCTGCACTCCGACCAGCAGTGATATTGGGAATTGAAGAGCCCTCTGTTATTGCCGCC |
| VLCHR1_2Phe | CCAATACGGATGACGGGGCTCTTCATCGATCACCATCTCCTGCACTTTCACCAGCAGTGATATTGGGAATTGAAGAGCCCTCTGTTATTGCCGCC |
| VLCHR1_2Tyr | CCAATACGGATGACGGGGCTCTTCATCGATCACCATCTCCTGCACTTATACCAGCAGTGATATTGGGAATTGAAGAGCCCTCTGTTATTGCCGCC |
| VLCHR1_2Trp | CCAATACGGATGACGGGGCTCTTCATCGATCACCATCTCCTGCACTTGGACCAGCAGTGATATTGGGAATTGAAGAGCCCTCTGTTATTGCCGCC |
| VLCHR1_2Ser | CCAATACGGATGACGGGGCTCTTCATCGATCACCATCTCCTGCACTAGCACCAGCAGTGATATTGGGAATTGAAGAGCCCTCTGTTATTGCCGCC |
| VLCHR1_2Thr | CCAATACGGATGACGGGGCTCTTCATCGATCACCATCTCCTGCACTACGACCAGCAGTGATATTGGGAATTGAAGAGCCCTCTGTTATTGCCGCC |
| VLCHR1_2Cys | CCAATACGGATGACGGGGCTCTTCATCGATCACCATCTCCTGCACTTGTACCAGCAGTGATATTGGGAATTGAAGAGCCCTCTGTTATTGCCGCC |
| VLCHR1_2Met | CCAATACGGATGACGGGGCTCTTCATCGATCACCATCTCCTGCACTATGACCAGCAGTGATATTGGGAATTGAAGAGCCCTCTGTTATTGCCGCC |
| VLCHR1_2Asn | CCAATACGGATGACGGGGCTCTTCATCGATCACCATCTCCTGCACTAACACCAGCAGTGATATTGGGAATTGAAGAGCCCTCTGTTATTGCCGCC |
| VLCHR1_2Gln | CCAATACGGATGACGGGGCTCTTCATCGATCACCATCTCCTGCACTCAAACCAGCAGTGATATTGGGAATTGAAGAGCCCTCTGTTATTGCCGCC |
| VLCHR1_2Asp | CCAATACGGATGACGGGGCTCTTCATCGATCACCATCTCCTGCACTGATACCAGCAGTGATATTGGGAATTGAAGAGCCCTCTGTTATTGCCGCC |
| VLCHR1_2Glu | CCAATACGGATGACGGGGCTCTTCATCGATCACCATCTCCTGCACTGAAACCAGCAGTGATATTGGGAATTGAAGAGCCCTCTGTTATTGCCGCC |
| VLCHR1_2Lys | CCAATACGGATGACGGGGCTCTTCATCGATCACCATCTCCTGCACTAAGACCAGCAGTGATATTGGGAATTGAAGAGCCCTCTGTTATTGCCGCC |
| VLCHR1_2Arg | CCAATACGGATGACGGGGCTCTTCATCGATCACCATCTCCTGCACTCGCACCAGCAGTGATATTGGGAATTGAAGAGCCCTCTGTTATTGCCGCC |
| VLCHR1_2His | CCAATACGGATGACGGGGCTCTTCATCGATCACCATCTCCTGCACTCACACCAGCAGTGATATTGGGAATTGAAGAGCCCTCTGTTATTGCCGCC |
| VLCHR1_3Gly | CCAATACGGATGACGGGGCTCTTCAATCACCATCTCCTGCACTGGAGGTAGCAGTGATATTGGGAATTATTGAAGAGCCCTCTGTTATTGCCGCC |
| VLCHR1_3Ala | CCAATACGGATGACGGGGCTCTTCAATCACCATCTCCTGCACTGGAGCGAGCAGTGATATTGGGAATTATTGAAGAGCCCTCTGTTATTGCCGCC |
| VLCHR1_3Val | CCAATACGGATGACGGGGCTCTTCAATCACCATCTCCTGCACTGGAGTAAGCAGTGATATTGGGAATTATTGAAGAGCCCTCTGTTATTGCCGCC |
| VLCHR1_3Leu | CCAATACGGATGACGGGGCTCTTCAATCACCATCTCCTGCACTGGACTTAGCAGTGATATTGGGAATTATTGAAGAGCCCTCTGTTATTGCCGCC |
| VLCHR1_3Ile | CCAATACGGATGACGGGGCTCTTCAATCACCATCTCCTGCACTGGAATTAGCAGTGATATTGGGAATTATTGAAGAGCCCTCTGTTATTGCCGCC |
| VLCHR1_3Pro | CCAATACGGATGACGGGGCTCTTCAATCACCATCTCCTGCACTGGACCAAGCAGTGATATTGGGAATTATTGAAGAGCCCTCTGTTATTGCCGCC |
| VLCHR1_3Phe | CCAATACGGATGACGGGGCTCTTCAATCACCATCTCCTGCACTGGATTTAGCAGTGATATTGGGAATTATTGAAGAGCCCTCTGTTATTGCCGCC |
| VLCHR1_3Tyr | CCAATACGGATGACGGGGCTCTTCAATCACCATCTCCTGCACTGGATACAGCAGTGATATTGGGAATTATTGAAGAGCCCTCTGTTATTGCCGCC |
| VLCHR1_3Trp | CCAATACGGATGACGGGGCTCTTCAATCACCATCTCCTGCACTGGATGGAGCAGTGATATTGGGAATTATTGAAGAGCCCTCTGTTATTGCCGCC |
| VLCHR1_3Ser | CCAATACGGATGACGGGGCTCTTCAATCACCATCTCCTGCACTGGAAGTAGCAGTGATATTGGGAATTATTGAAGAGCCCTCTGTTATTGCCGCC |
| VLCHR1_3Cys | CCAATACGGATGACGGGGCTCTTCAATCACCATCTCCTGCACTGGATGTAGCAGTGATATTGGGAATTATTGAAGAGCCCTCTGTTATTGCCGCC |
| VLCHR1_3Met | CCAATACGGATGACGGGGCTCTTCAATCACCATCTCCTGCACTGGAATGAGCAGTGATATTGGGAATTATTGAAGAGCCCTCTGTTATTGCCGCC |
| VLCHR1_3Asn | CCAATACGGATGACGGGGCTCTTCAATCACCATCTCCTGCACTGGAAACAGCAGTGATATTGGGAATTATTGAAGAGCCCTCTGTTATTGCCGCC |
| VLCHR1_3Gln | CCAATACGGATGACGGGGCTCTTCAATCACCATCTCCTGCACTGGACAGAGCAGTGATATTGGGAATTATTGAAGAGCCCTCTGTTATTGCCGCC |
| VLCHR1_3Asp | CCAATACGGATGACGGGGCTCTTCAATCACCATCTCCTGCACTGGAGATAGCAGTGATATTGGGAATTATTGAAGAGCCCTCTGTTATTGCCGCC |
| VLCHR1_3Glu | CCAATACGGATGACGGGGCTCTTCAATCACCATCTCCTGCACTGGAGAGAGCAGTGATATTGGGAATTATTGAAGAGCCCTCTGTTATTGCCGCC |
| VLCHR1_3Lys | CCAATACGGATGACGGGGCTCTTCAATCACCATCTCCTGCACTGGAAAAAGCAGTGATATTGGGAATTATTGAAGAGCCCTCTGTTATTGCCGCC |
| VLCHR1_3Arg | CCAATACGGATGACGGGGCTCTTCAATCACCATCTCCTGCACTGGAAGGAGCAGTGATATTGGGAATTATTGAAGAGCCCTCTGTTATTGCCGCC |
| VLCHR1_3His | CCAATACGGATGACGGGGCTCTTCAATCACCATCTCCTGCACTGGACATAGCAGTGATATTGGGAATTATTGAAGAGCCCTCTGTTATTGCCGCC |
| VLCHR1_4Gly | CCAATACGGATGACGGGGCTCTTCAACCATCTCCTGCACTGGAACCGGTAGTGATATTGGGAATTATAACTGAAGAGCCCTCTGTTATTGCCGCC |
| VLCHR1_4Ala | CCAATACGGATGACGGGGCTCTTCAACCATCTCCTGCACTGGAACCGCCAGTGATATTGGGAATTATAACTGAAGAGCCCTCTGTTATTGCCGCC |
| VLCHR1_4Val | CCAATACGGATGACGGGGCTCTTCAACCATCTCCTGCACTGGAACCGTGAGTGATATTGGGAATTATAACTGAAGAGCCCTCTGTTATTGCCGCC |
| VLCHR1_4Leu | CCAATACGGATGACGGGGCTCTTCAACCATCTCCTGCACTGGAACCTTAAGTGATATTGGGAATTATAACTGAAGAGCCCTCTGTTATTGCCGCC |
| VLCHR1_4Ile | CCAATACGGATGACGGGGCTCTTCAACCATCTCCTGCACTGGAACCATTAGTGATATTGGGAATTATAACTGAAGAGCCCTCTGTTATTGCCGCC |
| VLCHR1_4Pro | CCAATACGGATGACGGGGCTCTTCAACCATCTCCTGCACTGGAACCCCAAGTGATATTGGGAATTATAACTGAAGAGCCCTCTGTTATTGCCGCC |
| VLCHR1_4Phe | CCAATACGGATGACGGGGCTCTTCAACCATCTCCTGCACTGGAACCTTTAGTGATATTGGGAATTATAACTGAAGAGCCCTCTGTTATTGCCGCC |
| VLCHR1_4Tyr | CCAATACGGATGACGGGGCTCTTCAACCATCTCCTGCACTGGAACCTACAGTGATATTGGGAATTATAACTGAAGAGCCCTCTGTTATTGCCGCC |
| VLCHR1_4Trp | CCAATACGGATGACGGGGCTCTTCAACCATCTCCTGCACTGGAACCTGGAGTGATATTGGGAATTATAACTGAAGAGCCCTCTGTTATTGCCGCC |
| VLCHR1_4Thr | CCAATACGGATGACGGGGCTCTTCAACCATCTCCTGCACTGGAACCACGAGTGATATTGGGAATTATAACTGAAGAGCCCTCTGTTATTGCCGCC |
| VLCHR1_4Cys | CCAATACGGATGACGGGGCTCTTCAACCATCTCCTGCACTGGAACCTGTAGTGATATTGGGAATTATAACTGAAGAGCCCTCTGTTATTGCCGCC |
| VLCHR1_4Met | CCAATACGGATGACGGGGCTCTTCAACCATCTCCTGCACTGGAACCATGAGTGATATTGGGAATTATAACTGAAGAGCCCTCTGTTATTGCCGCC |
| VLCHR1_4Asn | CCAATACGGATGACGGGGCTCTTCAACCATCTCCTGCACTGGAACCAACAGTGATATTGGGAATTATAACTGAAGAGCCCTCTGTTATTGCCGCC |
| VLCHR1_4Gln | CCAATACGGATGACGGGGCTCTTCAACCATCTCCTGCACTGGAACCCAGAGTGATATTGGGAATTATAACTGAAGAGCCCTCTGTTATTGCCGCC |
| VLCHR1_4Asp | CCAATACGGATGACGGGGCTCTTCAACCATCTCCTGCACTGGAACCGACAGTGATATTGGGAATTATAACTGAAGAGCCCTCTGTTATTGCCGCC |
| VLCHR1_4Glu | CCAATACGGATGACGGGGCTCTTCAACCATCTCCTGCACTGGAACCGAAAGTGATATTGGGAATTATAACTGAAGAGCCCTCTGTTATTGCCGCC |
| VLCHR1_4Lys | CCAATACGGATGACGGGGCTCTTCAACCATCTCCTGCACTGGAACCAAAAGTGATATTGGGAATTATAACTGAAGAGCCCTCTGTTATTGCCGCC |
| VLCHR1_4Arg | CCAATACGGATGACGGGGCTCTTCAACCATCTCCTGCACTGGAACCCGTAGTGATATTGGGAATTATAACTGAAGAGCCCTCTGTTATTGCCGCC |
| VLCHR1_4His | CCAATACGGATGACGGGGCTCTTCAACCATCTCCTGCACTGGAACCCATAGTGATATTGGGAATTATAACTGAAGAGCCCTCTGTTATTGCCGCC |
| VLCHR1_5Gly | CCAATACGGATGACGGGGCTCTTCAATCTCCTGCACTGGAACCAGCGGGGATATTGGGAATTATAACCTTTGAAGAGCCCTCTGTTATTGCCGCC |
| VLCHR1_5Ala | CCAATACGGATGACGGGGCTCTTCAATCTCCTGCACTGGAACCAGCGCGGATATTGGGAATTATAACCTTTGAAGAGCCCTCTGTTATTGCCGCC |
| VLCHR1_5Val | CCAATACGGATGACGGGGCTCTTCAATCTCCTGCACTGGAACCAGCGTAGATATTGGGAATTATAACCTTTGAAGAGCCCTCTGTTATTGCCGCC |
| VLCHR1_5Leu | CCAATACGGATGACGGGGCTCTTCAATCTCCTGCACTGGAACCAGCCTGGATATTGGGAATTATAACCTTTGAAGAGCCCTCTGTTATTGCCGCC |
| VLCHR1_5Ile | CCAATACGGATGACGGGGCTCTTCAATCTCCTGCACTGGAACCAGCATAGATATTGGGAATTATAACCTTTGAAGAGCCCTCTGTTATTGCCGCC |
| VLCHR1_5Pro | CCAATACGGATGACGGGGCTCTTCAATCTCCTGCACTGGAACCAGCCCCGATATTGGGAATTATAACCTTTGAAGAGCCCTCTGTTATTGCCGCC |
| VLCHR1_5Phe | CCAATACGGATGACGGGGCTCTTCAATCTCCTGCACTGGAACCAGCTTCGATATTGGGAATTATAACCTTTGAAGAGCCCTCTGTTATTGCCGCC |
| VLCHR1_5Tyr | CCAATACGGATGACGGGGCTCTTCAATCTCCTGCACTGGAACCAGCTATGATATTGGGAATTATAACCTTTGAAGAGCCCTCTGTTATTGCCGCC |
| VLCHR1_5Trp | CCAATACGGATGACGGGGCTCTTCAATCTCCTGCACTGGAACCAGCTGGGATATTGGGAATTATAACCTTTGAAGAGCCCTCTGTTATTGCCGCC |
| VLCHR1_5Thr | CCAATACGGATGACGGGGCTCTTCAATCTCCTGCACTGGAACCAGCACGGATATTGGGAATTATAACCTTTGAAGAGCCCTCTGTTATTGCCGCC |
| VLCHR1_5Cys | CCAATACGGATGACGGGGCTCTTCAATCTCCTGCACTGGAACCAGCTGCGATATTGGGAATTATAACCTTTGAAGAGCCCTCTGTTATTGCCGCC |
| VLCHR1_5Met | CCAATACGGATGACGGGGCTCTTCAATCTCCTGCACTGGAACCAGCATGGATATTGGGAATTATAACCTTTGAAGAGCCCTCTGTTATTGCCGCC |
| VLCHR1_5Asn | CCAATACGGATGACGGGGCTCTTCAATCTCCTGCACTGGAACCAGCAACGATATTGGGAATTATAACCTTTGAAGAGCCCTCTGTTATTGCCGCC |
| VLCHR1_5Gln | CCAATACGGATGACGGGGCTCTTCAATCTCCTGCACTGGAACCAGCCAGGATATTGGGAATTATAACCTTTGAAGAGCCCTCTGTTATTGCCGCC |
| VLCHR1_5Asp | CCAATACGGATGACGGGGCTCTTCAATCTCCTGCACTGGAACCAGCGACGATATTGGGAATTATAACCTTTGAAGAGCCCTCTGTTATTGCCGCC |
| VLCHR1_5Glu | CCAATACGGATGACGGGGCTCTTCAATCTCCTGCACTGGAACCAGCGAGGATATTGGGAATTATAACCTTTGAAGAGCCCTCTGTTATTGCCGCC |
| VLCHR1_5Lys | CCAATACGGATGACGGGGCTCTTCAATCTCCTGCACTGGAACCAGCAAAGATATTGGGAATTATAACCTTTGAAGAGCCCTCTGTTATTGCCGCC |
| VLCHR1_5Arg | CCAATACGGATGACGGGGCTCTTCAATCTCCTGCACTGGAACCAGCCGGGATATTGGGAATTATAACCTTTGAAGAGCCCTCTGTTATTGCCGCC |
| VLCHR1_5His | CCAATACGGATGACGGGGCTCTTCAATCTCCTGCACTGGAACCAGCCATGATATTGGGAATTATAACCTTTGAAGAGCCCTCTGTTATTGCCGCC |
| VLCHR1_6Gly | CCAATACGGATGACGGGGCTCTTCATCCTGCACTGGAACCAGCAGTGGGATTGGGAATTATAACCTTGTCTGAAGAGCCCTCTGTTATTGCCGCC |
| VLCHR1_6Ala | CCAATACGGATGACGGGGCTCTTCATCCTGCACTGGAACCAGCAGTGCCATTGGGAATTATAACCTTGTCTGAAGAGCCCTCTGTTATTGCCGCC |
| VLCHR1_6Val | CCAATACGGATGACGGGGCTCTTCATCCTGCACTGGAACCAGCAGTGTAATTGGGAATTATAACCTTGTCTGAAGAGCCCTCTGTTATTGCCGCC |
| VLCHR1_6Leu | CCAATACGGATGACGGGGCTCTTCATCCTGCACTGGAACCAGCAGTTTAATTGGGAATTATAACCTTGTCTGAAGAGCCCTCTGTTATTGCCGCC |
| VLCHR1_6Ile | CCAATACGGATGACGGGGCTCTTCATCCTGCACTGGAACCAGCAGTATCATTGGGAATTATAACCTTGTCTGAAGAGCCCTCTGTTATTGCCGCC |
| VLCHR1_6Pro | CCAATACGGATGACGGGGCTCTTCATCCTGCACTGGAACCAGCAGTCCTATTGGGAATTATAACCTTGTCTGAAGAGCCCTCTGTTATTGCCGCC |
| VLCHR1_6Phe | CCAATACGGATGACGGGGCTCTTCATCCTGCACTGGAACCAGCAGTTTTATTGGGAATTATAACCTTGTCTGAAGAGCCCTCTGTTATTGCCGCC |
| VLCHR1_6Tyr | CCAATACGGATGACGGGGCTCTTCATCCTGCACTGGAACCAGCAGTTATATTGGGAATTATAACCTTGTCTGAAGAGCCCTCTGTTATTGCCGCC |
| VLCHR1_6Trp | CCAATACGGATGACGGGGCTCTTCATCCTGCACTGGAACCAGCAGTTGGATTGGGAATTATAACCTTGTCTGAAGAGCCCTCTGTTATTGCCGCC |
| VLCHR1_6Ser | CCAATACGGATGACGGGGCTCTTCATCCTGCACTGGAACCAGCAGTAGTATTGGGAATTATAACCTTGTCTGAAGAGCCCTCTGTTATTGCCGCC |
| VLCHR1_6Thr | CCAATACGGATGACGGGGCTCTTCATCCTGCACTGGAACCAGCAGTACAATTGGGAATTATAACCTTGTCTGAAGAGCCCTCTGTTATTGCCGCC |
| VLCHR1_6Cys | CCAATACGGATGACGGGGCTCTTCATCCTGCACTGGAACCAGCAGTTGTATTGGGAATTATAACCTTGTCTGAAGAGCCCTCTGTTATTGCCGCC |
| VLCHR1_6Met | CCAATACGGATGACGGGGCTCTTCATCCTGCACTGGAACCAGCAGTATGATTGGGAATTATAACCTTGTCTGAAGAGCCCTCTGTTATTGCCGCC |
| VLCHR1_6Asn | CCAATACGGATGACGGGGCTCTTCATCCTGCACTGGAACCAGCAGTAATATTGGGAATTATAACCTTGTCTGAAGAGCCCTCTGTTATTGCCGCC |
| VLCHR1_6Gln | CCAATACGGATGACGGGGCTCTTCATCCTGCACTGGAACCAGCAGTCAAATTGGGAATTATAACCTTGTCTGAAGAGCCCTCTGTTATTGCCGCC |
| VLCHR1_6Glu | CCAATACGGATGACGGGGCTCTTCATCCTGCACTGGAACCAGCAGTGAAATTGGGAATTATAACCTTGTCTGAAGAGCCCTCTGTTATTGCCGCC |
| VLCHR1_6Lys | CCAATACGGATGACGGGGCTCTTCATCCTGCACTGGAACCAGCAGTAAAATTGGGAATTATAACCTTGTCTGAAGAGCCCTCTGTTATTGCCGCC |
| VLCHR1_6Arg | CCAATACGGATGACGGGGCTCTTCATCCTGCACTGGAACCAGCAGTCGCATTGGGAATTATAACCTTGTCTGAAGAGCCCTCTGTTATTGCCGCC |
| VLCHR1_6His | CCAATACGGATGACGGGGCTCTTCATCCTGCACTGGAACCAGCAGTCACATTGGGAATTATAACCTTGTCTGAAGAGCCCTCTGTTATTGCCGCC |
| VLCHR1_7Gly | CCAATACGGATGACGGGGCTCTTCATGCACTGGAACCAGCAGTGATGGCGGGAATTATAACCTTGTCTCCTGAAGAGCCCTCTGTTATTGCCGCC |
| VLCHR1_7Ala | CCAATACGGATGACGGGGCTCTTCATGCACTGGAACCAGCAGTGATGCCGGGAATTATAACCTTGTCTCCTGAAGAGCCCTCTGTTATTGCCGCC |
| VLCHR1_7Val | CCAATACGGATGACGGGGCTCTTCATGCACTGGAACCAGCAGTGATGTAGGGAATTATAACCTTGTCTCCTGAAGAGCCCTCTGTTATTGCCGCC |
| VLCHR1_7Leu | CCAATACGGATGACGGGGCTCTTCATGCACTGGAACCAGCAGTGATCTCGGGAATTATAACCTTGTCTCCTGAAGAGCCCTCTGTTATTGCCGCC |
| VLCHR1_7Pro | CCAATACGGATGACGGGGCTCTTCATGCACTGGAACCAGCAGTGATCCAGGGAATTATAACCTTGTCTCCTGAAGAGCCCTCTGTTATTGCCGCC |
| VLCHR1_7Phe | CCAATACGGATGACGGGGCTCTTCATGCACTGGAACCAGCAGTGATTTTGGGAATTATAACCTTGTCTCCTGAAGAGCCCTCTGTTATTGCCGCC |
| VLCHR1_7Tyr | CCAATACGGATGACGGGGCTCTTCATGCACTGGAACCAGCAGTGATTATGGGAATTATAACCTTGTCTCCTGAAGAGCCCTCTGTTATTGCCGCC |
| VLCHR1_7Trp | CCAATACGGATGACGGGGCTCTTCATGCACTGGAACCAGCAGTGATTGGGGGAATTATAACCTTGTCTCCTGAAGAGCCCTCTGTTATTGCCGCC |
| VLCHR1_7Ser | CCAATACGGATGACGGGGCTCTTCATGCACTGGAACCAGCAGTGATTCGGGGAATTATAACCTTGTCTCCTGAAGAGCCCTCTGTTATTGCCGCC |
| VLCHR1_7Thr | CCAATACGGATGACGGGGCTCTTCATGCACTGGAACCAGCAGTGATACCGGGAATTATAACCTTGTCTCCTGAAGAGCCCTCTGTTATTGCCGCC |
| VLCHR1_7Cys | CCAATACGGATGACGGGGCTCTTCATGCACTGGAACCAGCAGTGATTGCGGGAATTATAACCTTGTCTCCTGAAGAGCCCTCTGTTATTGCCGCC |
| VLCHR1_7Met | CCAATACGGATGACGGGGCTCTTCATGCACTGGAACCAGCAGTGATATGGGGAATTATAACCTTGTCTCCTGAAGAGCCCTCTGTTATTGCCGCC |
| VLCHR1_7Asn | CCAATACGGATGACGGGGCTCTTCATGCACTGGAACCAGCAGTGATAACGGGAATTATAACCTTGTCTCCTGAAGAGCCCTCTGTTATTGCCGCC |
| VLCHR1_7Gln | CCAATACGGATGACGGGGCTCTTCATGCACTGGAACCAGCAGTGATCAGGGGAATTATAACCTTGTCTCCTGAAGAGCCCTCTGTTATTGCCGCC |
| VLCHR1_7Asp | CCAATACGGATGACGGGGCTCTTCATGCACTGGAACCAGCAGTGATGATGGGAATTATAACCTTGTCTCCTGAAGAGCCCTCTGTTATTGCCGCC |
| VLCHR1_7Glu | CCAATACGGATGACGGGGCTCTTCATGCACTGGAACCAGCAGTGATGAGGGGAATTATAACCTTGTCTCCTGAAGAGCCCTCTGTTATTGCCGCC |
| VLCHR1_7Lys | CCAATACGGATGACGGGGCTCTTCATGCACTGGAACCAGCAGTGATAAGGGGAATTATAACCTTGTCTCCTGAAGAGCCCTCTGTTATTGCCGCC |
| VLCHR1_7Arg | CCAATACGGATGACGGGGCTCTTCATGCACTGGAACCAGCAGTGATCGAGGGAATTATAACCTTGTCTCCTGAAGAGCCCTCTGTTATTGCCGCC |
| VLCHR1_7His | CCAATACGGATGACGGGGCTCTTCATGCACTGGAACCAGCAGTGATCATGGGAATTATAACCTTGTCTCCTGAAGAGCCCTCTGTTATTGCCGCC |
| VLCHR1_8Ala | CCAATACGGATGACGGGGCTCTTCAACTGGAACCAGCAGTGATATTGCGAATTATAACCTTGTCTCCTGGTGAAGAGCCCTCTGTTATTGCCGCC |
| VLCHR1_8Val | CCAATACGGATGACGGGGCTCTTCAACTGGAACCAGCAGTGATATTGTAAATTATAACCTTGTCTCCTGGTGAAGAGCCCTCTGTTATTGCCGCC |
| VLCHR1_8Leu | CCAATACGGATGACGGGGCTCTTCAACTGGAACCAGCAGTGATATTCTAAATTATAACCTTGTCTCCTGGTGAAGAGCCCTCTGTTATTGCCGCC |
| VLCHR1_8Ile | CCAATACGGATGACGGGGCTCTTCAACTGGAACCAGCAGTGATATTATCAATTATAACCTTGTCTCCTGGTGAAGAGCCCTCTGTTATTGCCGCC |
| VLCHR1_8Pro | CCAATACGGATGACGGGGCTCTTCAACTGGAACCAGCAGTGATATTCCAAATTATAACCTTGTCTCCTGGTGAAGAGCCCTCTGTTATTGCCGCC |
| VLCHR1_8Phe | CCAATACGGATGACGGGGCTCTTCAACTGGAACCAGCAGTGATATTTTTAATTATAACCTTGTCTCCTGGTGAAGAGCCCTCTGTTATTGCCGCC |
| VLCHR1_8Tyr | CCAATACGGATGACGGGGCTCTTCAACTGGAACCAGCAGTGATATTTACAATTATAACCTTGTCTCCTGGTGAAGAGCCCTCTGTTATTGCCGCC |
| VLCHR1_8Trp | CCAATACGGATGACGGGGCTCTTCAACTGGAACCAGCAGTGATATTTGGAATTATAACCTTGTCTCCTGGTGAAGAGCCCTCTGTTATTGCCGCC |
| VLCHR1_8Ser | CCAATACGGATGACGGGGCTCTTCAACTGGAACCAGCAGTGATATTTCAAATTATAACCTTGTCTCCTGGTGAAGAGCCCTCTGTTATTGCCGCC |
| VLCHR1_8Thr | CCAATACGGATGACGGGGCTCTTCAACTGGAACCAGCAGTGATATTACGAATTATAACCTTGTCTCCTGGTGAAGAGCCCTCTGTTATTGCCGCC |
| VLCHR1_8Cys | CCAATACGGATGACGGGGCTCTTCAACTGGAACCAGCAGTGATATTTGCAATTATAACCTTGTCTCCTGGTGAAGAGCCCTCTGTTATTGCCGCC |
| VLCHR1_8Met | CCAATACGGATGACGGGGCTCTTCAACTGGAACCAGCAGTGATATTATGAATTATAACCTTGTCTCCTGGTGAAGAGCCCTCTGTTATTGCCGCC |
| VLCHR1_8Asn | CCAATACGGATGACGGGGCTCTTCAACTGGAACCAGCAGTGATATTAATAATTATAACCTTGTCTCCTGGTGAAGAGCCCTCTGTTATTGCCGCC |
| VLCHR1_8Gln | CCAATACGGATGACGGGGCTCTTCAACTGGAACCAGCAGTGATATTCAAAATTATAACCTTGTCTCCTGGTGAAGAGCCCTCTGTTATTGCCGCC |
| VLCHR1_8Asp | CCAATACGGATGACGGGGCTCTTCAACTGGAACCAGCAGTGATATTGACAATTATAACCTTGTCTCCTGGTGAAGAGCCCTCTGTTATTGCCGCC |
| VLCHR1_8Glu | CCAATACGGATGACGGGGCTCTTCAACTGGAACCAGCAGTGATATTGAGAATTATAACCTTGTCTCCTGGTGAAGAGCCCTCTGTTATTGCCGCC |
| VLCHR1_8Lys | CCAATACGGATGACGGGGCTCTTCAACTGGAACCAGCAGTGATATTAAAAATTATAACCTTGTCTCCTGGTGAAGAGCCCTCTGTTATTGCCGCC |
| VLCHR1_8Arg | CCAATACGGATGACGGGGCTCTTCAACTGGAACCAGCAGTGATATTCGCAATTATAACCTTGTCTCCTGGTGAAGAGCCCTCTGTTATTGCCGCC |
| VLCHR1_8His | CCAATACGGATGACGGGGCTCTTCAACTGGAACCAGCAGTGATATTCACAATTATAACCTTGTCTCCTGGTGAAGAGCCCTCTGTTATTGCCGCC |
| VLCHR1_9Gly | CCAATACGGATGACGGGGCTCTTCAGGAACCAGCAGTGATATTGGGGGGTATAACCTTGTCTCCTGGTACTGAAGAGCCCTCTGTTATTGCCGCC |
| VLCHR1_9Ala | CCAATACGGATGACGGGGCTCTTCAGGAACCAGCAGTGATATTGGGGCTTATAACCTTGTCTCCTGGTACTGAAGAGCCCTCTGTTATTGCCGCC |
| VLCHR1_9Val | CCAATACGGATGACGGGGCTCTTCAGGAACCAGCAGTGATATTGGGGTCTATAACCTTGTCTCCTGGTACTGAAGAGCCCTCTGTTATTGCCGCC |
| VLCHR1_9Leu | CCAATACGGATGACGGGGCTCTTCAGGAACCAGCAGTGATATTGGGTTATATAACCTTGTCTCCTGGTACTGAAGAGCCCTCTGTTATTGCCGCC |
| VLCHR1_9Ile | CCAATACGGATGACGGGGCTCTTCAGGAACCAGCAGTGATATTGGGATCTATAACCTTGTCTCCTGGTACTGAAGAGCCCTCTGTTATTGCCGCC |
| VLCHR1_9Pro | CCAATACGGATGACGGGGCTCTTCAGGAACCAGCAGTGATATTGGGCCTTATAACCTTGTCTCCTGGTACTGAAGAGCCCTCTGTTATTGCCGCC |
| VLCHR1_9Phe | CCAATACGGATGACGGGGCTCTTCAGGAACCAGCAGTGATATTGGGTTCTATAACCTTGTCTCCTGGTACTGAAGAGCCCTCTGTTATTGCCGCC |
| VLCHR1_9Tyr | CCAATACGGATGACGGGGCTCTTCAGGAACCAGCAGTGATATTGGGTATTATAACCTTGTCTCCTGGTACTGAAGAGCCCTCTGTTATTGCCGCC |
| VLCHR1_9Trp | CCAATACGGATGACGGGGCTCTTCAGGAACCAGCAGTGATATTGGGTGGTATAACCTTGTCTCCTGGTACTGAAGAGCCCTCTGTTATTGCCGCC |
| VLCHR1_9Ser | CCAATACGGATGACGGGGCTCTTCAGGAACCAGCAGTGATATTGGGTCTTATAACCTTGTCTCCTGGTACTGAAGAGCCCTCTGTTATTGCCGCC |
| VLCHR1_9Thr | CCAATACGGATGACGGGGCTCTTCAGGAACCAGCAGTGATATTGGGACCTATAACCTTGTCTCCTGGTACTGAAGAGCCCTCTGTTATTGCCGCC |
| VLCHR1_9Cys | CCAATACGGATGACGGGGCTCTTCAGGAACCAGCAGTGATATTGGGTGTTATAACCTTGTCTCCTGGTACTGAAGAGCCCTCTGTTATTGCCGCC |
| VLCHR1_9Met | CCAATACGGATGACGGGGCTCTTCAGGAACCAGCAGTGATATTGGGATGTATAACCTTGTCTCCTGGTACTGAAGAGCCCTCTGTTATTGCCGCC |
| VLCHR1_9Gln | CCAATACGGATGACGGGGCTCTTCAGGAACCAGCAGTGATATTGGGCAGTATAACCTTGTCTCCTGGTACTGAAGAGCCCTCTGTTATTGCCGCC |
| VLCHR1_9Asp | CCAATACGGATGACGGGGCTCTTCAGGAACCAGCAGTGATATTGGGGATTATAACCTTGTCTCCTGGTACTGAAGAGCCCTCTGTTATTGCCGCC |
| VLCHR1_9Glu | CCAATACGGATGACGGGGCTCTTCAGGAACCAGCAGTGATATTGGGGAGTATAACCTTGTCTCCTGGTACTGAAGAGCCCTCTGTTATTGCCGCC |
| VLCHR1_9Lys | CCAATACGGATGACGGGGCTCTTCAGGAACCAGCAGTGATATTGGGAAATATAACCTTGTCTCCTGGTACTGAAGAGCCCTCTGTTATTGCCGCC |
| VLCHR1_9Arg | CCAATACGGATGACGGGGCTCTTCAGGAACCAGCAGTGATATTGGGCGCTATAACCTTGTCTCCTGGTACTGAAGAGCCCTCTGTTATTGCCGCC |
| VLCHR1_9His | CCAATACGGATGACGGGGCTCTTCAGGAACCAGCAGTGATATTGGGCACTATAACCTTGTCTCCTGGTACTGAAGAGCCCTCTGTTATTGCCGCC |
| VLCHR1_10Gly | CCAATACGGATGACGGGGCTCTTCAACCAGCAGTGATATTGGGAATGGGAACCTTGTCTCCTGGTACCAATGAAGAGCCCTCTGTTATTGCCGCC |
| VLCHR1_10Ala | CCAATACGGATGACGGGGCTCTTCAACCAGCAGTGATATTGGGAATGCCAACCTTGTCTCCTGGTACCAATGAAGAGCCCTCTGTTATTGCCGCC |
| VLCHR1_10Val | CCAATACGGATGACGGGGCTCTTCAACCAGCAGTGATATTGGGAATGTCAACCTTGTCTCCTGGTACCAATGAAGAGCCCTCTGTTATTGCCGCC |
| VLCHR1_10Leu | CCAATACGGATGACGGGGCTCTTCAACCAGCAGTGATATTGGGAATTTGAACCTTGTCTCCTGGTACCAATGAAGAGCCCTCTGTTATTGCCGCC |
| VLCHR1_10Ile | CCAATACGGATGACGGGGCTCTTCAACCAGCAGTGATATTGGGAATATAAACCTTGTCTCCTGGTACCAATGAAGAGCCCTCTGTTATTGCCGCC |
| VLCHR1_10Pro | CCAATACGGATGACGGGGCTCTTCAACCAGCAGTGATATTGGGAATCCCAACCTTGTCTCCTGGTACCAATGAAGAGCCCTCTGTTATTGCCGCC |
| VLCHR1_10Phe | CCAATACGGATGACGGGGCTCTTCAACCAGCAGTGATATTGGGAATTTCAACCTTGTCTCCTGGTACCAATGAAGAGCCCTCTGTTATTGCCGCC |
| VLCHR1_10Trp | CCAATACGGATGACGGGGCTCTTCAACCAGCAGTGATATTGGGAATTGGAACCTTGTCTCCTGGTACCAATGAAGAGCCCTCTGTTATTGCCGCC |
| VLCHR1_10Ser | CCAATACGGATGACGGGGCTCTTCAACCAGCAGTGATATTGGGAATTCTAACCTTGTCTCCTGGTACCAATGAAGAGCCCTCTGTTATTGCCGCC |
| VLCHR1_10Thr | CCAATACGGATGACGGGGCTCTTCAACCAGCAGTGATATTGGGAATACGAACCTTGTCTCCTGGTACCAATGAAGAGCCCTCTGTTATTGCCGCC |
| VLCHR1_10Cys | CCAATACGGATGACGGGGCTCTTCAACCAGCAGTGATATTGGGAATTGTAACCTTGTCTCCTGGTACCAATGAAGAGCCCTCTGTTATTGCCGCC |
| VLCHR1_10Met | CCAATACGGATGACGGGGCTCTTCAACCAGCAGTGATATTGGGAATATGAACCTTGTCTCCTGGTACCAATGAAGAGCCCTCTGTTATTGCCGCC |
| VLCHR1_10Asn | CCAATACGGATGACGGGGCTCTTCAACCAGCAGTGATATTGGGAATAATAACCTTGTCTCCTGGTACCAATGAAGAGCCCTCTGTTATTGCCGCC |
| VLCHR1_10Gln | CCAATACGGATGACGGGGCTCTTCAACCAGCAGTGATATTGGGAATCAGAACCTTGTCTCCTGGTACCAATGAAGAGCCCTCTGTTATTGCCGCC |
| VLCHR1_10Asp | CCAATACGGATGACGGGGCTCTTCAACCAGCAGTGATATTGGGAATGATAACCTTGTCTCCTGGTACCAATGAAGAGCCCTCTGTTATTGCCGCC |
| VLCHR1_10Glu | CCAATACGGATGACGGGGCTCTTCAACCAGCAGTGATATTGGGAATGAGAACCTTGTCTCCTGGTACCAATGAAGAGCCCTCTGTTATTGCCGCC |
| VLCHR1_10Lys | CCAATACGGATGACGGGGCTCTTCAACCAGCAGTGATATTGGGAATAAGAACCTTGTCTCCTGGTACCAATGAAGAGCCCTCTGTTATTGCCGCC |
| VLCHR1_10Arg | CCAATACGGATGACGGGGCTCTTCAACCAGCAGTGATATTGGGAATAGAAACCTTGTCTCCTGGTACCAATGAAGAGCCCTCTGTTATTGCCGCC |
| VLCHR1_10His | CCAATACGGATGACGGGGCTCTTCAACCAGCAGTGATATTGGGAATCATAACCTTGTCTCCTGGTACCAATGAAGAGCCCTCTGTTATTGCCGCC |
| VLCHR1_11Gly | CCAATACGGATGACGGGGCTCTTCAAGCAGTGATATTGGGAATTATGGGCTTGTCTCCTGGTACCAACAATGAAGAGCCCTCTGTTATTGCCGCC |
| VLCHR1_11Ala | CCAATACGGATGACGGGGCTCTTCAAGCAGTGATATTGGGAATTATGCTCTTGTCTCCTGGTACCAACAATGAAGAGCCCTCTGTTATTGCCGCC |
| VLCHR1_11Val | CCAATACGGATGACGGGGCTCTTCAAGCAGTGATATTGGGAATTATGTTCTTGTCTCCTGGTACCAACAATGAAGAGCCCTCTGTTATTGCCGCC |
| VLCHR1_11Leu | CCAATACGGATGACGGGGCTCTTCAAGCAGTGATATTGGGAATTATCTACTTGTCTCCTGGTACCAACAATGAAGAGCCCTCTGTTATTGCCGCC |
| VLCHR1_11Ile | CCAATACGGATGACGGGGCTCTTCAAGCAGTGATATTGGGAATTATATCCTTGTCTCCTGGTACCAACAATGAAGAGCCCTCTGTTATTGCCGCC |
| VLCHR1_11Pro | CCAATACGGATGACGGGGCTCTTCAAGCAGTGATATTGGGAATTATCCACTTGTCTCCTGGTACCAACAATGAAGAGCCCTCTGTTATTGCCGCC |
| VLCHR1_11Phe | CCAATACGGATGACGGGGCTCTTCAAGCAGTGATATTGGGAATTATTTCCTTGTCTCCTGGTACCAACAATGAAGAGCCCTCTGTTATTGCCGCC |
| VLCHR1_11Tyr | CCAATACGGATGACGGGGCTCTTCAAGCAGTGATATTGGGAATTATTACCTTGTCTCCTGGTACCAACAATGAAGAGCCCTCTGTTATTGCCGCC |
| VLCHR1_11Trp | CCAATACGGATGACGGGGCTCTTCAAGCAGTGATATTGGGAATTATTGGCTTGTCTCCTGGTACCAACAATGAAGAGCCCTCTGTTATTGCCGCC |
| VLCHR1_11Ser | CCAATACGGATGACGGGGCTCTTCAAGCAGTGATATTGGGAATTATAGTCTTGTCTCCTGGTACCAACAATGAAGAGCCCTCTGTTATTGCCGCC |
| VLCHR1_11Thr | CCAATACGGATGACGGGGCTCTTCAAGCAGTGATATTGGGAATTATACGCTTGTCTCCTGGTACCAACAATGAAGAGCCCTCTGTTATTGCCGCC |
| VLCHR1_11Cys | CCAATACGGATGACGGGGCTCTTCAAGCAGTGATATTGGGAATTATTGTCTTGTCTCCTGGTACCAACAATGAAGAGCCCTCTGTTATTGCCGCC |
| VLCHR1_11Met | CCAATACGGATGACGGGGCTCTTCAAGCAGTGATATTGGGAATTATATGCTTGTCTCCTGGTACCAACAATGAAGAGCCCTCTGTTATTGCCGCC |
| VLCHR1_11Gln | CCAATACGGATGACGGGGCTCTTCAAGCAGTGATATTGGGAATTATCAGCTTGTCTCCTGGTACCAACAATGAAGAGCCCTCTGTTATTGCCGCC |
| VLCHR1_11Asp | CCAATACGGATGACGGGGCTCTTCAAGCAGTGATATTGGGAATTATGACCTTGTCTCCTGGTACCAACAATGAAGAGCCCTCTGTTATTGCCGCC |
| VLCHR1_11Glu | CCAATACGGATGACGGGGCTCTTCAAGCAGTGATATTGGGAATTATGAACTTGTCTCCTGGTACCAACAATGAAGAGCCCTCTGTTATTGCCGCC |
| VLCHR1_11Lys | CCAATACGGATGACGGGGCTCTTCAAGCAGTGATATTGGGAATTATAAGCTTGTCTCCTGGTACCAACAATGAAGAGCCCTCTGTTATTGCCGCC |
| VLCHR1_11Arg | CCAATACGGATGACGGGGCTCTTCAAGCAGTGATATTGGGAATTATAGGCTTGTCTCCTGGTACCAACAATGAAGAGCCCTCTGTTATTGCCGCC |
| VLCHR1_11His | CCAATACGGATGACGGGGCTCTTCAAGCAGTGATATTGGGAATTATCATCTTGTCTCCTGGTACCAACAATGAAGAGCCCTCTGTTATTGCCGCC |
| VLCHR1_12Gly | CCAATACGGATGACGGGGCTCTTCAAGTGATATTGGGAATTATAACGGTGTCTCCTGGTACCAACAACACTGAAGAGCCCTCTGTTATTGCCGCC |
| VLCHR1_12Ala | CCAATACGGATGACGGGGCTCTTCAAGTGATATTGGGAATTATAACGCCGTCTCCTGGTACCAACAACACTGAAGAGCCCTCTGTTATTGCCGCC |
| VLCHR1_12Val | CCAATACGGATGACGGGGCTCTTCAAGTGATATTGGGAATTATAACGTTGTCTCCTGGTACCAACAACACTGAAGAGCCCTCTGTTATTGCCGCC |
| VLCHR1_12Ile | CCAATACGGATGACGGGGCTCTTCAAGTGATATTGGGAATTATAACATCGTCTCCTGGTACCAACAACACTGAAGAGCCCTCTGTTATTGCCGCC |
| VLCHR1_12Pro | CCAATACGGATGACGGGGCTCTTCAAGTGATATTGGGAATTATAACCCCGTCTCCTGGTACCAACAACACTGAAGAGCCCTCTGTTATTGCCGCC |
| VLCHR1_12Phe | CCAATACGGATGACGGGGCTCTTCAAGTGATATTGGGAATTATAACTTCGTCTCCTGGTACCAACAACACTGAAGAGCCCTCTGTTATTGCCGCC |
| VLCHR1_12Tyr | CCAATACGGATGACGGGGCTCTTCAAGTGATATTGGGAATTATAACTACGTCTCCTGGTACCAACAACACTGAAGAGCCCTCTGTTATTGCCGCC |
| VLCHR1_12Trp | CCAATACGGATGACGGGGCTCTTCAAGTGATATTGGGAATTATAACTGGGTCTCCTGGTACCAACAACACTGAAGAGCCCTCTGTTATTGCCGCC |
| VLCHR1_12Ser | CCAATACGGATGACGGGGCTCTTCAAGTGATATTGGGAATTATAACTCCGTCTCCTGGTACCAACAACACTGAAGAGCCCTCTGTTATTGCCGCC |
| VLCHR1_12Thr | CCAATACGGATGACGGGGCTCTTCAAGTGATATTGGGAATTATAACACCGTCTCCTGGTACCAACAACACTGAAGAGCCCTCTGTTATTGCCGCC |
| VLCHR1_12Cys | CCAATACGGATGACGGGGCTCTTCAAGTGATATTGGGAATTATAACTGTGTCTCCTGGTACCAACAACACTGAAGAGCCCTCTGTTATTGCCGCC |
| VLCHR1_12Met | CCAATACGGATGACGGGGCTCTTCAAGTGATATTGGGAATTATAACATGGTCTCCTGGTACCAACAACACTGAAGAGCCCTCTGTTATTGCCGCC |
| VLCHR1_12Asn | CCAATACGGATGACGGGGCTCTTCAAGTGATATTGGGAATTATAACAACGTCTCCTGGTACCAACAACACTGAAGAGCCCTCTGTTATTGCCGCC |
| VLCHR1_12Gln | CCAATACGGATGACGGGGCTCTTCAAGTGATATTGGGAATTATAACCAGGTCTCCTGGTACCAACAACACTGAAGAGCCCTCTGTTATTGCCGCC |
| VLCHR1_12Asp | CCAATACGGATGACGGGGCTCTTCAAGTGATATTGGGAATTATAACGATGTCTCCTGGTACCAACAACACTGAAGAGCCCTCTGTTATTGCCGCC |
| VLCHR1_12Glu | CCAATACGGATGACGGGGCTCTTCAAGTGATATTGGGAATTATAACGAAGTCTCCTGGTACCAACAACACTGAAGAGCCCTCTGTTATTGCCGCC |
| VLCHR1_12Lys | CCAATACGGATGACGGGGCTCTTCAAGTGATATTGGGAATTATAACAAAGTCTCCTGGTACCAACAACACTGAAGAGCCCTCTGTTATTGCCGCC |
| VLCHR1_12Arg | CCAATACGGATGACGGGGCTCTTCAAGTGATATTGGGAATTATAACAGAGTCTCCTGGTACCAACAACACTGAAGAGCCCTCTGTTATTGCCGCC |
| VLCHR1_12His | CCAATACGGATGACGGGGCTCTTCAAGTGATATTGGGAATTATAACCACGTCTCCTGGTACCAACAACACTGAAGAGCCCTCTGTTATTGCCGCC |
| VLCHR1_13Gly | CCAATACGGATGACGGGGCTCTTCAGATATTGGGAATTATAACCTTGGGTCCTGGTACCAACAACACCCATGAAGAGCCCTCTGTTATTGCCGCC |
| VLCHR1_13Ala | CCAATACGGATGACGGGGCTCTTCAGATATTGGGAATTATAACCTTGCGTCCTGGTACCAACAACACCCATGAAGAGCCCTCTGTTATTGCCGCC |
| VLCHR1_13Leu | CCAATACGGATGACGGGGCTCTTCAGATATTGGGAATTATAACCTTTTGTCCTGGTACCAACAACACCCATGAAGAGCCCTCTGTTATTGCCGCC |
| VLCHR1_13Ile | CCAATACGGATGACGGGGCTCTTCAGATATTGGGAATTATAACCTTATATCCTGGTACCAACAACACCCATGAAGAGCCCTCTGTTATTGCCGCC |
| VLCHR1_13Pro | CCAATACGGATGACGGGGCTCTTCAGATATTGGGAATTATAACCTTCCGTCCTGGTACCAACAACACCCATGAAGAGCCCTCTGTTATTGCCGCC |
| VLCHR1_13Phe | CCAATACGGATGACGGGGCTCTTCAGATATTGGGAATTATAACCTTTTTTCCTGGTACCAACAACACCCATGAAGAGCCCTCTGTTATTGCCGCC |
| VLCHR1_13Tyr | CCAATACGGATGACGGGGCTCTTCAGATATTGGGAATTATAACCTTTATTCCTGGTACCAACAACACCCATGAAGAGCCCTCTGTTATTGCCGCC |
| VLCHR1_13Trp | CCAATACGGATGACGGGGCTCTTCAGATATTGGGAATTATAACCTTTGGTCCTGGTACCAACAACACCCATGAAGAGCCCTCTGTTATTGCCGCC |
| VLCHR1_13Ser | CCAATACGGATGACGGGGCTCTTCAGATATTGGGAATTATAACCTTTCATCCTGGTACCAACAACACCCATGAAGAGCCCTCTGTTATTGCCGCC |
| VLCHR1_13Thr | CCAATACGGATGACGGGGCTCTTCAGATATTGGGAATTATAACCTTACTTCCTGGTACCAACAACACCCATGAAGAGCCCTCTGTTATTGCCGCC |
| VLCHR1_13Cys | CCAATACGGATGACGGGGCTCTTCAGATATTGGGAATTATAACCTTTGTTCCTGGTACCAACAACACCCATGAAGAGCCCTCTGTTATTGCCGCC |
| VLCHR1_13Met | CCAATACGGATGACGGGGCTCTTCAGATATTGGGAATTATAACCTTATGTCCTGGTACCAACAACACCCATGAAGAGCCCTCTGTTATTGCCGCC |
| VLCHR1_13Asn | CCAATACGGATGACGGGGCTCTTCAGATATTGGGAATTATAACCTTAACTCCTGGTACCAACAACACCCATGAAGAGCCCTCTGTTATTGCCGCC |
| VLCHR1_13Gln | CCAATACGGATGACGGGGCTCTTCAGATATTGGGAATTATAACCTTCAGTCCTGGTACCAACAACACCCATGAAGAGCCCTCTGTTATTGCCGCC |
| VLCHR1_13Asp | CCAATACGGATGACGGGGCTCTTCAGATATTGGGAATTATAACCTTGACTCCTGGTACCAACAACACCCATGAAGAGCCCTCTGTTATTGCCGCC |
| VLCHR1_13Glu | CCAATACGGATGACGGGGCTCTTCAGATATTGGGAATTATAACCTTGAATCCTGGTACCAACAACACCCATGAAGAGCCCTCTGTTATTGCCGCC |
| VLCHR1_13Lys | CCAATACGGATGACGGGGCTCTTCAGATATTGGGAATTATAACCTTAAGTCCTGGTACCAACAACACCCATGAAGAGCCCTCTGTTATTGCCGCC |
| VLCHR1_13Arg | CCAATACGGATGACGGGGCTCTTCAGATATTGGGAATTATAACCTTCGGTCCTGGTACCAACAACACCCATGAAGAGCCCTCTGTTATTGCCGCC |
| VLCHR1_13His | CCAATACGGATGACGGGGCTCTTCAGATATTGGGAATTATAACCTTCATTCCTGGTACCAACAACACCCATGAAGAGCCCTCTGTTATTGCCGCC |
| VLCHR1_14Gly | CCAATACGGATGACGGGGCTCTTCAATTGGGAATTATAACCTTGTCGGGTGGTACCAACAACACCCAGGCTGAAGAGCCCTCTGTTATTGCCGCC |
| VLCHR1_14Ala | CCAATACGGATGACGGGGCTCTTCAATTGGGAATTATAACCTTGTCGCATGGTACCAACAACACCCAGGCTGAAGAGCCCTCTGTTATTGCCGCC |
| VLCHR1_14Val | CCAATACGGATGACGGGGCTCTTCAATTGGGAATTATAACCTTGTCGTATGGTACCAACAACACCCAGGCTGAAGAGCCCTCTGTTATTGCCGCC |
| VLCHR1_14Leu | CCAATACGGATGACGGGGCTCTTCAATTGGGAATTATAACCTTGTCTTGTGGTACCAACAACACCCAGGCTGAAGAGCCCTCTGTTATTGCCGCC |
| VLCHR1_14Ile | CCAATACGGATGACGGGGCTCTTCAATTGGGAATTATAACCTTGTCATTTGGTACCAACAACACCCAGGCTGAAGAGCCCTCTGTTATTGCCGCC |
| VLCHR1_14Pro | CCAATACGGATGACGGGGCTCTTCAATTGGGAATTATAACCTTGTCCCTTGGTACCAACAACACCCAGGCTGAAGAGCCCTCTGTTATTGCCGCC |
| VLCHR1_14Phe | CCAATACGGATGACGGGGCTCTTCAATTGGGAATTATAACCTTGTCTTCTGGTACCAACAACACCCAGGCTGAAGAGCCCTCTGTTATTGCCGCC |
| VLCHR1_14Tyr | CCAATACGGATGACGGGGCTCTTCAATTGGGAATTATAACCTTGTCTACTGGTACCAACAACACCCAGGCTGAAGAGCCCTCTGTTATTGCCGCC |
| VLCHR1_14Trp | CCAATACGGATGACGGGGCTCTTCAATTGGGAATTATAACCTTGTCTGGTGGTACCAACAACACCCAGGCTGAAGAGCCCTCTGTTATTGCCGCC |
| VLCHR1_14Thr | CCAATACGGATGACGGGGCTCTTCAATTGGGAATTATAACCTTGTCACTTGGTACCAACAACACCCAGGCTGAAGAGCCCTCTGTTATTGCCGCC |
| VLCHR1_14Cys | CCAATACGGATGACGGGGCTCTTCAATTGGGAATTATAACCTTGTCTGTTGGTACCAACAACACCCAGGCTGAAGAGCCCTCTGTTATTGCCGCC |
| VLCHR1_14Met | CCAATACGGATGACGGGGCTCTTCAATTGGGAATTATAACCTTGTCATGTGGTACCAACAACACCCAGGCTGAAGAGCCCTCTGTTATTGCCGCC |
| VLCHR1_14Asn | CCAATACGGATGACGGGGCTCTTCAATTGGGAATTATAACCTTGTCAACTGGTACCAACAACACCCAGGCTGAAGAGCCCTCTGTTATTGCCGCC |
| VLCHR1_14Gln | CCAATACGGATGACGGGGCTCTTCAATTGGGAATTATAACCTTGTCCAGTGGTACCAACAACACCCAGGCTGAAGAGCCCTCTGTTATTGCCGCC |
| VLCHR1_14Asp | CCAATACGGATGACGGGGCTCTTCAATTGGGAATTATAACCTTGTCGATTGGTACCAACAACACCCAGGCTGAAGAGCCCTCTGTTATTGCCGCC |
| VLCHR1_14Glu | CCAATACGGATGACGGGGCTCTTCAATTGGGAATTATAACCTTGTCGAGTGGTACCAACAACACCCAGGCTGAAGAGCCCTCTGTTATTGCCGCC |
| VLCHR1_14Lys | CCAATACGGATGACGGGGCTCTTCAATTGGGAATTATAACCTTGTCAAATGGTACCAACAACACCCAGGCTGAAGAGCCCTCTGTTATTGCCGCC |
| VLCHR1_14Arg | CCAATACGGATGACGGGGCTCTTCAATTGGGAATTATAACCTTGTCCGCTGGTACCAACAACACCCAGGCTGAAGAGCCCTCTGTTATTGCCGCC |
| VLCHR1_14His | CCAATACGGATGACGGGGCTCTTCAATTGGGAATTATAACCTTGTCCATTGGTACCAACAACACCCAGGCTGAAGAGCCCTCTGTTATTGCCGCC |
| VLCHR2_1Gly | CCAATACGGATGACGGGGCTCTTCAGCCCCCAAACTCATAATTTATGGAGTCACTAAGCGGCCCTCAGGGTGAAGAGCCCTCTGTTATTGCCGCC |
| VLCHR2_1Ala | CCAATACGGATGACGGGGCTCTTCAGCCCCCAAACTCATAATTTATGCCGTCACTAAGCGGCCCTCAGGGTGAAGAGCCCTCTGTTATTGCCGCC |
| VLCHR2_1Val | CCAATACGGATGACGGGGCTCTTCAGCCCCCAAACTCATAATTTATGTGGTCACTAAGCGGCCCTCAGGGTGAAGAGCCCTCTGTTATTGCCGCC |
| VLCHR2_1Leu | CCAATACGGATGACGGGGCTCTTCAGCCCCCAAACTCATAATTTATTTAGTCACTAAGCGGCCCTCAGGGTGAAGAGCCCTCTGTTATTGCCGCC |
| VLCHR2_1Ile | CCAATACGGATGACGGGGCTCTTCAGCCCCCAAACTCATAATTTATATAGTCACTAAGCGGCCCTCAGGGTGAAGAGCCCTCTGTTATTGCCGCC |
| VLCHR2_1Pro | CCAATACGGATGACGGGGCTCTTCAGCCCCCAAACTCATAATTTATCCCGTCACTAAGCGGCCCTCAGGGTGAAGAGCCCTCTGTTATTGCCGCC |
| VLCHR2_1Phe | CCAATACGGATGACGGGGCTCTTCAGCCCCCAAACTCATAATTTATTTCGTCACTAAGCGGCCCTCAGGGTGAAGAGCCCTCTGTTATTGCCGCC |
| VLCHR2_1Tyr | CCAATACGGATGACGGGGCTCTTCAGCCCCCAAACTCATAATTTATTATGTCACTAAGCGGCCCTCAGGGTGAAGAGCCCTCTGTTATTGCCGCC |
| VLCHR2_1Trp | CCAATACGGATGACGGGGCTCTTCAGCCCCCAAACTCATAATTTATTGGGTCACTAAGCGGCCCTCAGGGTGAAGAGCCCTCTGTTATTGCCGCC |
| VLCHR2_1Ser | CCAATACGGATGACGGGGCTCTTCAGCCCCCAAACTCATAATTTATTCGGTCACTAAGCGGCCCTCAGGGTGAAGAGCCCTCTGTTATTGCCGCC |
| VLCHR2_1Thr | CCAATACGGATGACGGGGCTCTTCAGCCCCCAAACTCATAATTTATACAGTCACTAAGCGGCCCTCAGGGTGAAGAGCCCTCTGTTATTGCCGCC |
| VLCHR2_1Cys | CCAATACGGATGACGGGGCTCTTCAGCCCCCAAACTCATAATTTATTGTGTCACTAAGCGGCCCTCAGGGTGAAGAGCCCTCTGTTATTGCCGCC |
| VLCHR2_1Met | CCAATACGGATGACGGGGCTCTTCAGCCCCCAAACTCATAATTTATATGGTCACTAAGCGGCCCTCAGGGTGAAGAGCCCTCTGTTATTGCCGCC |
| VLCHR2_1Asn | CCAATACGGATGACGGGGCTCTTCAGCCCCCAAACTCATAATTTATAATGTCACTAAGCGGCCCTCAGGGTGAAGAGCCCTCTGTTATTGCCGCC |
| VLCHR2_1Gln | CCAATACGGATGACGGGGCTCTTCAGCCCCCAAACTCATAATTTATCAGGTCACTAAGCGGCCCTCAGGGTGAAGAGCCCTCTGTTATTGCCGCC |
| VLCHR2_1Asp | CCAATACGGATGACGGGGCTCTTCAGCCCCCAAACTCATAATTTATGACGTCACTAAGCGGCCCTCAGGGTGAAGAGCCCTCTGTTATTGCCGCC |
| VLCHR2_1Lys | CCAATACGGATGACGGGGCTCTTCAGCCCCCAAACTCATAATTTATAAAGTCACTAAGCGGCCCTCAGGGTGAAGAGCCCTCTGTTATTGCCGCC |
| VLCHR2_1Arg | CCAATACGGATGACGGGGCTCTTCAGCCCCCAAACTCATAATTTATCGCGTCACTAAGCGGCCCTCAGGGTGAAGAGCCCTCTGTTATTGCCGCC |
| VLCHR2_1His | CCAATACGGATGACGGGGCTCTTCAGCCCCCAAACTCATAATTTATCATGTCACTAAGCGGCCCTCAGGGTGAAGAGCCCTCTGTTATTGCCGCC |
| VLCHR2_2Gly | CCAATACGGATGACGGGGCTCTTCACCCAAACTCATAATTTATGAGGGGACTAAGCGGCCCTCAGGGGTTTGAAGAGCCCTCTGTTATTGCCGCC |
| VLCHR2_2Ala | CCAATACGGATGACGGGGCTCTTCACCCAAACTCATAATTTATGAGGCAACTAAGCGGCCCTCAGGGGTTTGAAGAGCCCTCTGTTATTGCCGCC |
| VLCHR2_2Leu | CCAATACGGATGACGGGGCTCTTCACCCAAACTCATAATTTATGAGTTAACTAAGCGGCCCTCAGGGGTTTGAAGAGCCCTCTGTTATTGCCGCC |
| VLCHR2_2Ile | CCAATACGGATGACGGGGCTCTTCACCCAAACTCATAATTTATGAGATTACTAAGCGGCCCTCAGGGGTTTGAAGAGCCCTCTGTTATTGCCGCC |
| VLCHR2_2Pro | CCAATACGGATGACGGGGCTCTTCACCCAAACTCATAATTTATGAGCCCACTAAGCGGCCCTCAGGGGTTTGAAGAGCCCTCTGTTATTGCCGCC |
| VLCHR2_2Phe | CCAATACGGATGACGGGGCTCTTCACCCAAACTCATAATTTATGAGTTTACTAAGCGGCCCTCAGGGGTTTGAAGAGCCCTCTGTTATTGCCGCC |
| VLCHR2_2Tyr | CCAATACGGATGACGGGGCTCTTCACCCAAACTCATAATTTATGAGTATACTAAGCGGCCCTCAGGGGTTTGAAGAGCCCTCTGTTATTGCCGCC |
| VLCHR2_2Trp | CCAATACGGATGACGGGGCTCTTCACCCAAACTCATAATTTATGAGTGGACTAAGCGGCCCTCAGGGGTTTGAAGAGCCCTCTGTTATTGCCGCC |
| VLCHR2_2Ser | CCAATACGGATGACGGGGCTCTTCACCCAAACTCATAATTTATGAGAGCACTAAGCGGCCCTCAGGGGTTTGAAGAGCCCTCTGTTATTGCCGCC |
| VLCHR2_2Thr | CCAATACGGATGACGGGGCTCTTCACCCAAACTCATAATTTATGAGACCACTAAGCGGCCCTCAGGGGTTTGAAGAGCCCTCTGTTATTGCCGCC |
| VLCHR2_2Cys | CCAATACGGATGACGGGGCTCTTCACCCAAACTCATAATTTATGAGTGTACTAAGCGGCCCTCAGGGGTTTGAAGAGCCCTCTGTTATTGCCGCC |
| VLCHR2_2Met | CCAATACGGATGACGGGGCTCTTCACCCAAACTCATAATTTATGAGATGACTAAGCGGCCCTCAGGGGTTTGAAGAGCCCTCTGTTATTGCCGCC |
| VLCHR2_2Asn | CCAATACGGATGACGGGGCTCTTCACCCAAACTCATAATTTATGAGAACACTAAGCGGCCCTCAGGGGTTTGAAGAGCCCTCTGTTATTGCCGCC |
| VLCHR2_2Gln | CCAATACGGATGACGGGGCTCTTCACCCAAACTCATAATTTATGAGCAGACTAAGCGGCCCTCAGGGGTTTGAAGAGCCCTCTGTTATTGCCGCC |
| VLCHR2_2Asp | CCAATACGGATGACGGGGCTCTTCACCCAAACTCATAATTTATGAGGATACTAAGCGGCCCTCAGGGGTTTGAAGAGCCCTCTGTTATTGCCGCC |
| VLCHR2_2Glu | CCAATACGGATGACGGGGCTCTTCACCCAAACTCATAATTTATGAGGAAACTAAGCGGCCCTCAGGGGTTTGAAGAGCCCTCTGTTATTGCCGCC |
| VLCHR2_2Lys | CCAATACGGATGACGGGGCTCTTCACCCAAACTCATAATTTATGAGAAAACTAAGCGGCCCTCAGGGGTTTGAAGAGCCCTCTGTTATTGCCGCC |
| VLCHR2_2Arg | CCAATACGGATGACGGGGCTCTTCACCCAAACTCATAATTTATGAGCGAACTAAGCGGCCCTCAGGGGTTTGAAGAGCCCTCTGTTATTGCCGCC |
| VLCHR2_2His | CCAATACGGATGACGGGGCTCTTCACCCAAACTCATAATTTATGAGCATACTAAGCGGCCCTCAGGGGTTTGAAGAGCCCTCTGTTATTGCCGCC |
| VLCHR2_3Gly | CCAATACGGATGACGGGGCTCTTCAAAACTCATAATTTATGAGGTCGGGAAGCGGCCCTCAGGGGTTTCTTGAAGAGCCCTCTGTTATTGCCGCC |
| VLCHR2_3Ala | CCAATACGGATGACGGGGCTCTTCAAAACTCATAATTTATGAGGTCGCAAAGCGGCCCTCAGGGGTTTCTTGAAGAGCCCTCTGTTATTGCCGCC |
| VLCHR2_3Val | CCAATACGGATGACGGGGCTCTTCAAAACTCATAATTTATGAGGTCGTTAAGCGGCCCTCAGGGGTTTCTTGAAGAGCCCTCTGTTATTGCCGCC |
| VLCHR2_3Leu | CCAATACGGATGACGGGGCTCTTCAAAACTCATAATTTATGAGGTCTTAAAGCGGCCCTCAGGGGTTTCTTGAAGAGCCCTCTGTTATTGCCGCC |
| VLCHR2_3Ile | CCAATACGGATGACGGGGCTCTTCAAAACTCATAATTTATGAGGTCATCAAGCGGCCCTCAGGGGTTTCTTGAAGAGCCCTCTGTTATTGCCGCC |
| VLCHR2_3Pro | CCAATACGGATGACGGGGCTCTTCAAAACTCATAATTTATGAGGTCCCCAAGCGGCCCTCAGGGGTTTCTTGAAGAGCCCTCTGTTATTGCCGCC |
| VLCHR2_3Phe | CCAATACGGATGACGGGGCTCTTCAAAACTCATAATTTATGAGGTCTTCAAGCGGCCCTCAGGGGTTTCTTGAAGAGCCCTCTGTTATTGCCGCC |
| VLCHR2_3Tyr | CCAATACGGATGACGGGGCTCTTCAAAACTCATAATTTATGAGGTCTATAAGCGGCCCTCAGGGGTTTCTTGAAGAGCCCTCTGTTATTGCCGCC |
| VLCHR2_3Trp | CCAATACGGATGACGGGGCTCTTCAAAACTCATAATTTATGAGGTCTGGAAGCGGCCCTCAGGGGTTTCTTGAAGAGCCCTCTGTTATTGCCGCC |
| VLCHR2_3Ser | CCAATACGGATGACGGGGCTCTTCAAAACTCATAATTTATGAGGTCTCAAAGCGGCCCTCAGGGGTTTCTTGAAGAGCCCTCTGTTATTGCCGCC |
| VLCHR2_3Cys | CCAATACGGATGACGGGGCTCTTCAAAACTCATAATTTATGAGGTCTGCAAGCGGCCCTCAGGGGTTTCTTGAAGAGCCCTCTGTTATTGCCGCC |
| VLCHR2_3Met | CCAATACGGATGACGGGGCTCTTCAAAACTCATAATTTATGAGGTCATGAAGCGGCCCTCAGGGGTTTCTTGAAGAGCCCTCTGTTATTGCCGCC |
| VLCHR2_3Asn | CCAATACGGATGACGGGGCTCTTCAAAACTCATAATTTATGAGGTCAACAAGCGGCCCTCAGGGGTTTCTTGAAGAGCCCTCTGTTATTGCCGCC |
| VLCHR2_3Gln | CCAATACGGATGACGGGGCTCTTCAAAACTCATAATTTATGAGGTCCAAAAGCGGCCCTCAGGGGTTTCTTGAAGAGCCCTCTGTTATTGCCGCC |
| VLCHR2_3Asp | CCAATACGGATGACGGGGCTCTTCAAAACTCATAATTTATGAGGTCGACAAGCGGCCCTCAGGGGTTTCTTGAAGAGCCCTCTGTTATTGCCGCC |
| VLCHR2_3Glu | CCAATACGGATGACGGGGCTCTTCAAAACTCATAATTTATGAGGTCGAAAAGCGGCCCTCAGGGGTTTCTTGAAGAGCCCTCTGTTATTGCCGCC |
| VLCHR2_3Lys | CCAATACGGATGACGGGGCTCTTCAAAACTCATAATTTATGAGGTCAAGAAGCGGCCCTCAGGGGTTTCTTGAAGAGCCCTCTGTTATTGCCGCC |
| VLCHR2_3Arg | CCAATACGGATGACGGGGCTCTTCAAAACTCATAATTTATGAGGTCAGGAAGCGGCCCTCAGGGGTTTCTTGAAGAGCCCTCTGTTATTGCCGCC |
| VLCHR2_3His | CCAATACGGATGACGGGGCTCTTCAAAACTCATAATTTATGAGGTCCACAAGCGGCCCTCAGGGGTTTCTTGAAGAGCCCTCTGTTATTGCCGCC |
| VLCHR2_4Gly | CCAATACGGATGACGGGGCTCTTCACTCATAATTTATGAGGTCACTGGGCGGCCCTCAGGGGTTTCTAATTGAAGAGCCCTCTGTTATTGCCGCC |
| VLCHR2_4Ala | CCAATACGGATGACGGGGCTCTTCACTCATAATTTATGAGGTCACTGCCCGGCCCTCAGGGGTTTCTAATTGAAGAGCCCTCTGTTATTGCCGCC |
| VLCHR2_4Val | CCAATACGGATGACGGGGCTCTTCACTCATAATTTATGAGGTCACTGTACGGCCCTCAGGGGTTTCTAATTGAAGAGCCCTCTGTTATTGCCGCC |
| VLCHR2_4Leu | CCAATACGGATGACGGGGCTCTTCACTCATAATTTATGAGGTCACTCTCCGGCCCTCAGGGGTTTCTAATTGAAGAGCCCTCTGTTATTGCCGCC |
| VLCHR2_4Ile | CCAATACGGATGACGGGGCTCTTCACTCATAATTTATGAGGTCACTATCCGGCCCTCAGGGGTTTCTAATTGAAGAGCCCTCTGTTATTGCCGCC |
| VLCHR2_4Pro | CCAATACGGATGACGGGGCTCTTCACTCATAATTTATGAGGTCACTCCGCGGCCCTCAGGGGTTTCTAATTGAAGAGCCCTCTGTTATTGCCGCC |
| VLCHR2_4Phe | CCAATACGGATGACGGGGCTCTTCACTCATAATTTATGAGGTCACTTTTCGGCCCTCAGGGGTTTCTAATTGAAGAGCCCTCTGTTATTGCCGCC |
| VLCHR2_4Tyr | CCAATACGGATGACGGGGCTCTTCACTCATAATTTATGAGGTCACTTATCGGCCCTCAGGGGTTTCTAATTGAAGAGCCCTCTGTTATTGCCGCC |
| VLCHR2_4Trp | CCAATACGGATGACGGGGCTCTTCACTCATAATTTATGAGGTCACTTGGCGGCCCTCAGGGGTTTCTAATTGAAGAGCCCTCTGTTATTGCCGCC |
| VLCHR2_4Ser | CCAATACGGATGACGGGGCTCTTCACTCATAATTTATGAGGTCACTAGCCGGCCCTCAGGGGTTTCTAATTGAAGAGCCCTCTGTTATTGCCGCC |
| VLCHR2_4Thr | CCAATACGGATGACGGGGCTCTTCACTCATAATTTATGAGGTCACTACACGGCCCTCAGGGGTTTCTAATTGAAGAGCCCTCTGTTATTGCCGCC |
| VLCHR2_4Cys | CCAATACGGATGACGGGGCTCTTCACTCATAATTTATGAGGTCACTTGCCGGCCCTCAGGGGTTTCTAATTGAAGAGCCCTCTGTTATTGCCGCC |
| VLCHR2_4Met | CCAATACGGATGACGGGGCTCTTCACTCATAATTTATGAGGTCACTATGCGGCCCTCAGGGGTTTCTAATTGAAGAGCCCTCTGTTATTGCCGCC |
| VLCHR2_4Asn | CCAATACGGATGACGGGGCTCTTCACTCATAATTTATGAGGTCACTAATCGGCCCTCAGGGGTTTCTAATTGAAGAGCCCTCTGTTATTGCCGCC |
| VLCHR2_4Gln | CCAATACGGATGACGGGGCTCTTCACTCATAATTTATGAGGTCACTCAGCGGCCCTCAGGGGTTTCTAATTGAAGAGCCCTCTGTTATTGCCGCC |
| VLCHR2_4Asp | CCAATACGGATGACGGGGCTCTTCACTCATAATTTATGAGGTCACTGACCGGCCCTCAGGGGTTTCTAATTGAAGAGCCCTCTGTTATTGCCGCC |
| VLCHR2_4Glu | CCAATACGGATGACGGGGCTCTTCACTCATAATTTATGAGGTCACTGAACGGCCCTCAGGGGTTTCTAATTGAAGAGCCCTCTGTTATTGCCGCC |
| VLCHR2_4Arg | CCAATACGGATGACGGGGCTCTTCACTCATAATTTATGAGGTCACTCGTCGGCCCTCAGGGGTTTCTAATTGAAGAGCCCTCTGTTATTGCCGCC |
| VLCHR2_4His | CCAATACGGATGACGGGGCTCTTCACTCATAATTTATGAGGTCACTCACCGGCCCTCAGGGGTTTCTAATTGAAGAGCCCTCTGTTATTGCCGCC |
| VLCHR2_5Gly | CCAATACGGATGACGGGGCTCTTCAATAATTTATGAGGTCACTAAGGGTCCCTCAGGGGTTTCTAATCGCTGAAGAGCCCTCTGTTATTGCCGCC |
| VLCHR2_5Ala | CCAATACGGATGACGGGGCTCTTCAATAATTTATGAGGTCACTAAGGCGCCCTCAGGGGTTTCTAATCGCTGAAGAGCCCTCTGTTATTGCCGCC |
| VLCHR2_5Val | CCAATACGGATGACGGGGCTCTTCAATAATTTATGAGGTCACTAAGGTGCCCTCAGGGGTTTCTAATCGCTGAAGAGCCCTCTGTTATTGCCGCC |
| VLCHR2_5Leu | CCAATACGGATGACGGGGCTCTTCAATAATTTATGAGGTCACTAAGTTGCCCTCAGGGGTTTCTAATCGCTGAAGAGCCCTCTGTTATTGCCGCC |
| VLCHR2_5Ile | CCAATACGGATGACGGGGCTCTTCAATAATTTATGAGGTCACTAAGATTCCCTCAGGGGTTTCTAATCGCTGAAGAGCCCTCTGTTATTGCCGCC |
| VLCHR2_5Pro | CCAATACGGATGACGGGGCTCTTCAATAATTTATGAGGTCACTAAGCCGCCCTCAGGGGTTTCTAATCGCTGAAGAGCCCTCTGTTATTGCCGCC |
| VLCHR2_5Phe | CCAATACGGATGACGGGGCTCTTCAATAATTTATGAGGTCACTAAGTTTCCCTCAGGGGTTTCTAATCGCTGAAGAGCCCTCTGTTATTGCCGCC |
| VLCHR2_5Tyr | CCAATACGGATGACGGGGCTCTTCAATAATTTATGAGGTCACTAAGTATCCCTCAGGGGTTTCTAATCGCTGAAGAGCCCTCTGTTATTGCCGCC |
| VLCHR2_5Trp | CCAATACGGATGACGGGGCTCTTCAATAATTTATGAGGTCACTAAGTGGCCCTCAGGGGTTTCTAATCGCTGAAGAGCCCTCTGTTATTGCCGCC |
| VLCHR2_5Ser | CCAATACGGATGACGGGGCTCTTCAATAATTTATGAGGTCACTAAGTCGCCCTCAGGGGTTTCTAATCGCTGAAGAGCCCTCTGTTATTGCCGCC |
| VLCHR2_5Thr | CCAATACGGATGACGGGGCTCTTCAATAATTTATGAGGTCACTAAGACACCCTCAGGGGTTTCTAATCGCTGAAGAGCCCTCTGTTATTGCCGCC |
| VLCHR2_5Cys | CCAATACGGATGACGGGGCTCTTCAATAATTTATGAGGTCACTAAGTGTCCCTCAGGGGTTTCTAATCGCTGAAGAGCCCTCTGTTATTGCCGCC |
| VLCHR2_5Met | CCAATACGGATGACGGGGCTCTTCAATAATTTATGAGGTCACTAAGATGCCCTCAGGGGTTTCTAATCGCTGAAGAGCCCTCTGTTATTGCCGCC |
| VLCHR2_5Asn | CCAATACGGATGACGGGGCTCTTCAATAATTTATGAGGTCACTAAGAATCCCTCAGGGGTTTCTAATCGCTGAAGAGCCCTCTGTTATTGCCGCC |
| VLCHR2_5Gln | CCAATACGGATGACGGGGCTCTTCAATAATTTATGAGGTCACTAAGCAGCCCTCAGGGGTTTCTAATCGCTGAAGAGCCCTCTGTTATTGCCGCC |
| VLCHR2_5Asp | CCAATACGGATGACGGGGCTCTTCAATAATTTATGAGGTCACTAAGGATCCCTCAGGGGTTTCTAATCGCTGAAGAGCCCTCTGTTATTGCCGCC |
| VLCHR2_5Glu | CCAATACGGATGACGGGGCTCTTCAATAATTTATGAGGTCACTAAGGAGCCCTCAGGGGTTTCTAATCGCTGAAGAGCCCTCTGTTATTGCCGCC |
| VLCHR2_5Lys | CCAATACGGATGACGGGGCTCTTCAATAATTTATGAGGTCACTAAGAAGCCCTCAGGGGTTTCTAATCGCTGAAGAGCCCTCTGTTATTGCCGCC |
| VLCHR2_5His | CCAATACGGATGACGGGGCTCTTCAATAATTTATGAGGTCACTAAGCACCCCTCAGGGGTTTCTAATCGCTGAAGAGCCCTCTGTTATTGCCGCC |
| VLCHR2_6Gly | CCAATACGGATGACGGGGCTCTTCAATTTATGAGGTCACTAAGCGGGGTTCAGGGGTTTCTAATCGCTTCTGAAGAGCCCTCTGTTATTGCCGCC |
| VLCHR2_6Ala | CCAATACGGATGACGGGGCTCTTCAATTTATGAGGTCACTAAGCGGGCTTCAGGGGTTTCTAATCGCTTCTGAAGAGCCCTCTGTTATTGCCGCC |
| VLCHR2_6Val | CCAATACGGATGACGGGGCTCTTCAATTTATGAGGTCACTAAGCGGGTATCAGGGGTTTCTAATCGCTTCTGAAGAGCCCTCTGTTATTGCCGCC |
| VLCHR2_6Leu | CCAATACGGATGACGGGGCTCTTCAATTTATGAGGTCACTAAGCGGCTATCAGGGGTTTCTAATCGCTTCTGAAGAGCCCTCTGTTATTGCCGCC |
| VLCHR2_6Ile | CCAATACGGATGACGGGGCTCTTCAATTTATGAGGTCACTAAGCGGATCTCAGGGGTTTCTAATCGCTTCTGAAGAGCCCTCTGTTATTGCCGCC |
| VLCHR2_6Phe | CCAATACGGATGACGGGGCTCTTCAATTTATGAGGTCACTAAGCGGTTCTCAGGGGTTTCTAATCGCTTCTGAAGAGCCCTCTGTTATTGCCGCC |
| VLCHR2_6Tyr | CCAATACGGATGACGGGGCTCTTCAATTTATGAGGTCACTAAGCGGTACTCAGGGGTTTCTAATCGCTTCTGAAGAGCCCTCTGTTATTGCCGCC |
| VLCHR2_6Trp | CCAATACGGATGACGGGGCTCTTCAATTTATGAGGTCACTAAGCGGTGGTCAGGGGTTTCTAATCGCTTCTGAAGAGCCCTCTGTTATTGCCGCC |
| VLCHR2_6Ser | CCAATACGGATGACGGGGCTCTTCAATTTATGAGGTCACTAAGCGGTCATCAGGGGTTTCTAATCGCTTCTGAAGAGCCCTCTGTTATTGCCGCC |
| VLCHR2_6Thr | CCAATACGGATGACGGGGCTCTTCAATTTATGAGGTCACTAAGCGGACATCAGGGGTTTCTAATCGCTTCTGAAGAGCCCTCTGTTATTGCCGCC |
| VLCHR2_6Cys | CCAATACGGATGACGGGGCTCTTCAATTTATGAGGTCACTAAGCGGTGTTCAGGGGTTTCTAATCGCTTCTGAAGAGCCCTCTGTTATTGCCGCC |
| VLCHR2_6Met | CCAATACGGATGACGGGGCTCTTCAATTTATGAGGTCACTAAGCGGATGTCAGGGGTTTCTAATCGCTTCTGAAGAGCCCTCTGTTATTGCCGCC |
| VLCHR2_6Asn | CCAATACGGATGACGGGGCTCTTCAATTTATGAGGTCACTAAGCGGAATTCAGGGGTTTCTAATCGCTTCTGAAGAGCCCTCTGTTATTGCCGCC |
| VLCHR2_6Gln | CCAATACGGATGACGGGGCTCTTCAATTTATGAGGTCACTAAGCGGCAGTCAGGGGTTTCTAATCGCTTCTGAAGAGCCCTCTGTTATTGCCGCC |
| VLCHR2_6Asp | CCAATACGGATGACGGGGCTCTTCAATTTATGAGGTCACTAAGCGGGACTCAGGGGTTTCTAATCGCTTCTGAAGAGCCCTCTGTTATTGCCGCC |
| VLCHR2_6Glu | CCAATACGGATGACGGGGCTCTTCAATTTATGAGGTCACTAAGCGGGAGTCAGGGGTTTCTAATCGCTTCTGAAGAGCCCTCTGTTATTGCCGCC |
| VLCHR2_6Lys | CCAATACGGATGACGGGGCTCTTCAATTTATGAGGTCACTAAGCGGAAATCAGGGGTTTCTAATCGCTTCTGAAGAGCCCTCTGTTATTGCCGCC |
| VLCHR2_6Arg | CCAATACGGATGACGGGGCTCTTCAATTTATGAGGTCACTAAGCGGCGATCAGGGGTTTCTAATCGCTTCTGAAGAGCCCTCTGTTATTGCCGCC |
| VLCHR2_6His | CCAATACGGATGACGGGGCTCTTCAATTTATGAGGTCACTAAGCGGCACTCAGGGGTTTCTAATCGCTTCTGAAGAGCCCTCTGTTATTGCCGCC |
| VLCHR2_7Gly | CCAATACGGATGACGGGGCTCTTCATATGAGGTCACTAAGCGGCCCGGGGGGGTTTCTAATCGCTTCTCTTGAAGAGCCCTCTGTTATTGCCGCC |
| VLCHR2_7Ala | CCAATACGGATGACGGGGCTCTTCATATGAGGTCACTAAGCGGCCCGCTGGGGTTTCTAATCGCTTCTCTTGAAGAGCCCTCTGTTATTGCCGCC |
| VLCHR2_7Val | CCAATACGGATGACGGGGCTCTTCATATGAGGTCACTAAGCGGCCCGTTGGGGTTTCTAATCGCTTCTCTTGAAGAGCCCTCTGTTATTGCCGCC |
| VLCHR2_7Leu | CCAATACGGATGACGGGGCTCTTCATATGAGGTCACTAAGCGGCCCCTAGGGGTTTCTAATCGCTTCTCTTGAAGAGCCCTCTGTTATTGCCGCC |
| VLCHR2_7Ile | CCAATACGGATGACGGGGCTCTTCATATGAGGTCACTAAGCGGCCCATAGGGGTTTCTAATCGCTTCTCTTGAAGAGCCCTCTGTTATTGCCGCC |
| VLCHR2_7Pro | CCAATACGGATGACGGGGCTCTTCATATGAGGTCACTAAGCGGCCCCCTGGGGTTTCTAATCGCTTCTCTTGAAGAGCCCTCTGTTATTGCCGCC |
| VLCHR2_7Phe | CCAATACGGATGACGGGGCTCTTCATATGAGGTCACTAAGCGGCCCTTCGGGGTTTCTAATCGCTTCTCTTGAAGAGCCCTCTGTTATTGCCGCC |
| VLCHR2_7Tyr | CCAATACGGATGACGGGGCTCTTCATATGAGGTCACTAAGCGGCCCTACGGGGTTTCTAATCGCTTCTCTTGAAGAGCCCTCTGTTATTGCCGCC |
| VLCHR2_7Trp | CCAATACGGATGACGGGGCTCTTCATATGAGGTCACTAAGCGGCCCTGGGGGGTTTCTAATCGCTTCTCTTGAAGAGCCCTCTGTTATTGCCGCC |
| VLCHR2_7Thr | CCAATACGGATGACGGGGCTCTTCATATGAGGTCACTAAGCGGCCCACCGGGGTTTCTAATCGCTTCTCTTGAAGAGCCCTCTGTTATTGCCGCC |
| VLCHR2_7Cys | CCAATACGGATGACGGGGCTCTTCATATGAGGTCACTAAGCGGCCCTGTGGGGTTTCTAATCGCTTCTCTTGAAGAGCCCTCTGTTATTGCCGCC |
| VLCHR2_7Met | CCAATACGGATGACGGGGCTCTTCATATGAGGTCACTAAGCGGCCCATGGGGGTTTCTAATCGCTTCTCTTGAAGAGCCCTCTGTTATTGCCGCC |
| VLCHR2_7Asn | CCAATACGGATGACGGGGCTCTTCATATGAGGTCACTAAGCGGCCCAATGGGGTTTCTAATCGCTTCTCTTGAAGAGCCCTCTGTTATTGCCGCC |
| VLCHR2_7Gln | CCAATACGGATGACGGGGCTCTTCATATGAGGTCACTAAGCGGCCCCAGGGGGTTTCTAATCGCTTCTCTTGAAGAGCCCTCTGTTATTGCCGCC |
| VLCHR2_7Asp | CCAATACGGATGACGGGGCTCTTCATATGAGGTCACTAAGCGGCCCGATGGGGTTTCTAATCGCTTCTCTTGAAGAGCCCTCTGTTATTGCCGCC |
| VLCHR2_7Glu | CCAATACGGATGACGGGGCTCTTCATATGAGGTCACTAAGCGGCCCGAGGGGGTTTCTAATCGCTTCTCTTGAAGAGCCCTCTGTTATTGCCGCC |
| VLCHR2_7Lys | CCAATACGGATGACGGGGCTCTTCATATGAGGTCACTAAGCGGCCCAAGGGGGTTTCTAATCGCTTCTCTTGAAGAGCCCTCTGTTATTGCCGCC |
| VLCHR2_7Arg | CCAATACGGATGACGGGGCTCTTCATATGAGGTCACTAAGCGGCCCCGCGGGGTTTCTAATCGCTTCTCTTGAAGAGCCCTCTGTTATTGCCGCC |
| VLCHR2_7His | CCAATACGGATGACGGGGCTCTTCATATGAGGTCACTAAGCGGCCCCACGGGGTTTCTAATCGCTTCTCTTGAAGAGCCCTCTGTTATTGCCGCC |
| VLCHR3_1Gly | CCAATACGGATGACGGGGCTCTTCAGACGAGGCTAATTACTACTGCGGATCATATACAGCCACCAAGAATTGAAGAGCCCTCTGTTATTGCCGCC |
| VLCHR3_1Ala | CCAATACGGATGACGGGGCTCTTCAGACGAGGCTAATTACTACTGCGCGTCATATACAGCCACCAAGAATTGAAGAGCCCTCTGTTATTGCCGCC |
| VLCHR3_1Val | CCAATACGGATGACGGGGCTCTTCAGACGAGGCTAATTACTACTGCGTCTCATATACAGCCACCAAGAATTGAAGAGCCCTCTGTTATTGCCGCC |
| VLCHR3_1Leu | CCAATACGGATGACGGGGCTCTTCAGACGAGGCTAATTACTACTGCTTGTCATATACAGCCACCAAGAATTGAAGAGCCCTCTGTTATTGCCGCC |
| VLCHR3_1Ile | CCAATACGGATGACGGGGCTCTTCAGACGAGGCTAATTACTACTGCATCTCATATACAGCCACCAAGAATTGAAGAGCCCTCTGTTATTGCCGCC |
| VLCHR3_1Pro | CCAATACGGATGACGGGGCTCTTCAGACGAGGCTAATTACTACTGCCCATCATATACAGCCACCAAGAATTGAAGAGCCCTCTGTTATTGCCGCC |
| VLCHR3_1Phe | CCAATACGGATGACGGGGCTCTTCAGACGAGGCTAATTACTACTGCTTCTCATATACAGCCACCAAGAATTGAAGAGCCCTCTGTTATTGCCGCC |
| VLCHR3_1Tyr | CCAATACGGATGACGGGGCTCTTCAGACGAGGCTAATTACTACTGCTATTCATATACAGCCACCAAGAATTGAAGAGCCCTCTGTTATTGCCGCC |
| VLCHR3_1Trp | CCAATACGGATGACGGGGCTCTTCAGACGAGGCTAATTACTACTGCTGGTCATATACAGCCACCAAGAATTGAAGAGCCCTCTGTTATTGCCGCC |
| VLCHR3_1Thr | CCAATACGGATGACGGGGCTCTTCAGACGAGGCTAATTACTACTGCACTTCATATACAGCCACCAAGAATTGAAGAGCCCTCTGTTATTGCCGCC |
| VLCHR3_1Cys | CCAATACGGATGACGGGGCTCTTCAGACGAGGCTAATTACTACTGCTGCTCATATACAGCCACCAAGAATTGAAGAGCCCTCTGTTATTGCCGCC |
| VLCHR3_1Met | CCAATACGGATGACGGGGCTCTTCAGACGAGGCTAATTACTACTGCATGTCATATACAGCCACCAAGAATTGAAGAGCCCTCTGTTATTGCCGCC |
| VLCHR3_1Asn | CCAATACGGATGACGGGGCTCTTCAGACGAGGCTAATTACTACTGCAATTCATATACAGCCACCAAGAATTGAAGAGCCCTCTGTTATTGCCGCC |
| VLCHR3_1Gln | CCAATACGGATGACGGGGCTCTTCAGACGAGGCTAATTACTACTGCCAGTCATATACAGCCACCAAGAATTGAAGAGCCCTCTGTTATTGCCGCC |
| VLCHR3_1Asp | CCAATACGGATGACGGGGCTCTTCAGACGAGGCTAATTACTACTGCGACTCATATACAGCCACCAAGAATTGAAGAGCCCTCTGTTATTGCCGCC |
| VLCHR3_1Glu | CCAATACGGATGACGGGGCTCTTCAGACGAGGCTAATTACTACTGCGAATCATATACAGCCACCAAGAATTGAAGAGCCCTCTGTTATTGCCGCC |
| VLCHR3_1Lys | CCAATACGGATGACGGGGCTCTTCAGACGAGGCTAATTACTACTGCAAGTCATATACAGCCACCAAGAATTGAAGAGCCCTCTGTTATTGCCGCC |
| VLCHR3_1Arg | CCAATACGGATGACGGGGCTCTTCAGACGAGGCTAATTACTACTGCCGATCATATACAGCCACCAAGAATTGAAGAGCCCTCTGTTATTGCCGCC |
| VLCHR3_1His | CCAATACGGATGACGGGGCTCTTCAGACGAGGCTAATTACTACTGCCACTCATATACAGCCACCAAGAATTGAAGAGCCCTCTGTTATTGCCGCC |
| VLCHR3_2Gly | CCAATACGGATGACGGGGCTCTTCAGAGGCTAATTACTACTGCAGCGGTTATACAGCCACCAAGAATTACTGAAGAGCCCTCTGTTATTGCCGCC |
| VLCHR3_2Ala | CCAATACGGATGACGGGGCTCTTCAGAGGCTAATTACTACTGCAGCGCGTATACAGCCACCAAGAATTACTGAAGAGCCCTCTGTTATTGCCGCC |
| VLCHR3_2Val | CCAATACGGATGACGGGGCTCTTCAGAGGCTAATTACTACTGCAGCGTGTATACAGCCACCAAGAATTACTGAAGAGCCCTCTGTTATTGCCGCC |
| VLCHR3_2Leu | CCAATACGGATGACGGGGCTCTTCAGAGGCTAATTACTACTGCAGCCTATATACAGCCACCAAGAATTACTGAAGAGCCCTCTGTTATTGCCGCC |
| VLCHR3_2Ile | CCAATACGGATGACGGGGCTCTTCAGAGGCTAATTACTACTGCAGCATATATACAGCCACCAAGAATTACTGAAGAGCCCTCTGTTATTGCCGCC |
| VLCHR3_2Pro | CCAATACGGATGACGGGGCTCTTCAGAGGCTAATTACTACTGCAGCCCTTATACAGCCACCAAGAATTACTGAAGAGCCCTCTGTTATTGCCGCC |
| VLCHR3_2Phe | CCAATACGGATGACGGGGCTCTTCAGAGGCTAATTACTACTGCAGCTTCTATACAGCCACCAAGAATTACTGAAGAGCCCTCTGTTATTGCCGCC |
| VLCHR3_2Tyr | CCAATACGGATGACGGGGCTCTTCAGAGGCTAATTACTACTGCAGCTATTATACAGCCACCAAGAATTACTGAAGAGCCCTCTGTTATTGCCGCC |
| VLCHR3_2Trp | CCAATACGGATGACGGGGCTCTTCAGAGGCTAATTACTACTGCAGCTGGTATACAGCCACCAAGAATTACTGAAGAGCCCTCTGTTATTGCCGCC |
| VLCHR3_2Thr | CCAATACGGATGACGGGGCTCTTCAGAGGCTAATTACTACTGCAGCACTTATACAGCCACCAAGAATTACTGAAGAGCCCTCTGTTATTGCCGCC |
| VLCHR3_2Cys | CCAATACGGATGACGGGGCTCTTCAGAGGCTAATTACTACTGCAGCTGTTATACAGCCACCAAGAATTACTGAAGAGCCCTCTGTTATTGCCGCC |
| VLCHR3_2Met | CCAATACGGATGACGGGGCTCTTCAGAGGCTAATTACTACTGCAGCATGTATACAGCCACCAAGAATTACTGAAGAGCCCTCTGTTATTGCCGCC |
| VLCHR3_2Asn | CCAATACGGATGACGGGGCTCTTCAGAGGCTAATTACTACTGCAGCAATTATACAGCCACCAAGAATTACTGAAGAGCCCTCTGTTATTGCCGCC |
| VLCHR3_2Gln | CCAATACGGATGACGGGGCTCTTCAGAGGCTAATTACTACTGCAGCCAATATACAGCCACCAAGAATTACTGAAGAGCCCTCTGTTATTGCCGCC |
| VLCHR3_2Asp | CCAATACGGATGACGGGGCTCTTCAGAGGCTAATTACTACTGCAGCGATTATACAGCCACCAAGAATTACTGAAGAGCCCTCTGTTATTGCCGCC |
| VLCHR3_2Glu | CCAATACGGATGACGGGGCTCTTCAGAGGCTAATTACTACTGCAGCGAATATACAGCCACCAAGAATTACTGAAGAGCCCTCTGTTATTGCCGCC |
| VLCHR3_2Lys | CCAATACGGATGACGGGGCTCTTCAGAGGCTAATTACTACTGCAGCAAGTATACAGCCACCAAGAATTACTGAAGAGCCCTCTGTTATTGCCGCC |
| VLCHR3_2Arg | CCAATACGGATGACGGGGCTCTTCAGAGGCTAATTACTACTGCAGCAGGTATACAGCCACCAAGAATTACTGAAGAGCCCTCTGTTATTGCCGCC |
| VLCHR3_2His | CCAATACGGATGACGGGGCTCTTCAGAGGCTAATTACTACTGCAGCCATTATACAGCCACCAAGAATTACTGAAGAGCCCTCTGTTATTGCCGCC |
| VLCHR3_3Gly | CCAATACGGATGACGGGGCTCTTCAGCTAATTACTACTGCAGCTCAGGCACAGCCACCAAGAATTACTGGTGAAGAGCCCTCTGTTATTGCCGCC |
| VLCHR3_3Ala | CCAATACGGATGACGGGGCTCTTCAGCTAATTACTACTGCAGCTCAGCCACAGCCACCAAGAATTACTGGTGAAGAGCCCTCTGTTATTGCCGCC |
| VLCHR3_3Val | CCAATACGGATGACGGGGCTCTTCAGCTAATTACTACTGCAGCTCAGTAACAGCCACCAAGAATTACTGGTGAAGAGCCCTCTGTTATTGCCGCC |
| VLCHR3_3Leu | CCAATACGGATGACGGGGCTCTTCAGCTAATTACTACTGCAGCTCATTGACAGCCACCAAGAATTACTGGTGAAGAGCCCTCTGTTATTGCCGCC |
| VLCHR3_3Ile | CCAATACGGATGACGGGGCTCTTCAGCTAATTACTACTGCAGCTCAATCACAGCCACCAAGAATTACTGGTGAAGAGCCCTCTGTTATTGCCGCC |
| VLCHR3_3Pro | CCAATACGGATGACGGGGCTCTTCAGCTAATTACTACTGCAGCTCACCAACAGCCACCAAGAATTACTGGTGAAGAGCCCTCTGTTATTGCCGCC |
| VLCHR3_3Phe | CCAATACGGATGACGGGGCTCTTCAGCTAATTACTACTGCAGCTCATTTACAGCCACCAAGAATTACTGGTGAAGAGCCCTCTGTTATTGCCGCC |
| VLCHR3_3Trp | CCAATACGGATGACGGGGCTCTTCAGCTAATTACTACTGCAGCTCATGGACAGCCACCAAGAATTACTGGTGAAGAGCCCTCTGTTATTGCCGCC |
| VLCHR3_3Ser | CCAATACGGATGACGGGGCTCTTCAGCTAATTACTACTGCAGCTCATCTACAGCCACCAAGAATTACTGGTGAAGAGCCCTCTGTTATTGCCGCC |
| VLCHR3_3Thr | CCAATACGGATGACGGGGCTCTTCAGCTAATTACTACTGCAGCTCAACTACAGCCACCAAGAATTACTGGTGAAGAGCCCTCTGTTATTGCCGCC |
| VLCHR3_3Cys | CCAATACGGATGACGGGGCTCTTCAGCTAATTACTACTGCAGCTCATGCACAGCCACCAAGAATTACTGGTGAAGAGCCCTCTGTTATTGCCGCC |
| VLCHR3_3Met | CCAATACGGATGACGGGGCTCTTCAGCTAATTACTACTGCAGCTCAATGACAGCCACCAAGAATTACTGGTGAAGAGCCCTCTGTTATTGCCGCC |
| VLCHR3_3Asn | CCAATACGGATGACGGGGCTCTTCAGCTAATTACTACTGCAGCTCAAACACAGCCACCAAGAATTACTGGTGAAGAGCCCTCTGTTATTGCCGCC |
| VLCHR3_3Gln | CCAATACGGATGACGGGGCTCTTCAGCTAATTACTACTGCAGCTCACAAACAGCCACCAAGAATTACTGGTGAAGAGCCCTCTGTTATTGCCGCC |
| VLCHR3_3Asp | CCAATACGGATGACGGGGCTCTTCAGCTAATTACTACTGCAGCTCAGATACAGCCACCAAGAATTACTGGTGAAGAGCCCTCTGTTATTGCCGCC |
| VLCHR3_3Glu | CCAATACGGATGACGGGGCTCTTCAGCTAATTACTACTGCAGCTCAGAAACAGCCACCAAGAATTACTGGTGAAGAGCCCTCTGTTATTGCCGCC |
| VLCHR3_3Lys | CCAATACGGATGACGGGGCTCTTCAGCTAATTACTACTGCAGCTCAAAAACAGCCACCAAGAATTACTGGTGAAGAGCCCTCTGTTATTGCCGCC |
| VLCHR3_3Arg | CCAATACGGATGACGGGGCTCTTCAGCTAATTACTACTGCAGCTCACGCACAGCCACCAAGAATTACTGGTGAAGAGCCCTCTGTTATTGCCGCC |
| VLCHR3_3His | CCAATACGGATGACGGGGCTCTTCAGCTAATTACTACTGCAGCTCACACACAGCCACCAAGAATTACTGGTGAAGAGCCCTCTGTTATTGCCGCC |
| VLCHR3_4Gly | CCAATACGGATGACGGGGCTCTTCAAATTACTACTGCAGCTCATATGGTGCCACCAAGAATTACTGGATTTGAAGAGCCCTCTGTTATTGCCGCC |
| VLCHR3_4Ala | CCAATACGGATGACGGGGCTCTTCAAATTACTACTGCAGCTCATATGCAGCCACCAAGAATTACTGGATTTGAAGAGCCCTCTGTTATTGCCGCC |
| VLCHR3_4Val | CCAATACGGATGACGGGGCTCTTCAAATTACTACTGCAGCTCATATGTGGCCACCAAGAATTACTGGATTTGAAGAGCCCTCTGTTATTGCCGCC |
| VLCHR3_4Leu | CCAATACGGATGACGGGGCTCTTCAAATTACTACTGCAGCTCATATTTGGCCACCAAGAATTACTGGATTTGAAGAGCCCTCTGTTATTGCCGCC |
| VLCHR3_4Ile | CCAATACGGATGACGGGGCTCTTCAAATTACTACTGCAGCTCATATATAGCCACCAAGAATTACTGGATTTGAAGAGCCCTCTGTTATTGCCGCC |
| VLCHR3_4Pro | CCAATACGGATGACGGGGCTCTTCAAATTACTACTGCAGCTCATATCCCGCCACCAAGAATTACTGGATTTGAAGAGCCCTCTGTTATTGCCGCC |
| VLCHR3_4Phe | CCAATACGGATGACGGGGCTCTTCAAATTACTACTGCAGCTCATATTTTGCCACCAAGAATTACTGGATTTGAAGAGCCCTCTGTTATTGCCGCC |
| VLCHR3_4Tyr | CCAATACGGATGACGGGGCTCTTCAAATTACTACTGCAGCTCATATTATGCCACCAAGAATTACTGGATTTGAAGAGCCCTCTGTTATTGCCGCC |
| VLCHR3_4Trp | CCAATACGGATGACGGGGCTCTTCAAATTACTACTGCAGCTCATATTGGGCCACCAAGAATTACTGGATTTGAAGAGCCCTCTGTTATTGCCGCC |
| VLCHR3_4Ser | CCAATACGGATGACGGGGCTCTTCAAATTACTACTGCAGCTCATATTCAGCCACCAAGAATTACTGGATTTGAAGAGCCCTCTGTTATTGCCGCC |
| VLCHR3_4Cys | CCAATACGGATGACGGGGCTCTTCAAATTACTACTGCAGCTCATATTGCGCCACCAAGAATTACTGGATTTGAAGAGCCCTCTGTTATTGCCGCC |
| VLCHR3_4Met | CCAATACGGATGACGGGGCTCTTCAAATTACTACTGCAGCTCATATATGGCCACCAAGAATTACTGGATTTGAAGAGCCCTCTGTTATTGCCGCC |
| VLCHR3_4Asn | CCAATACGGATGACGGGGCTCTTCAAATTACTACTGCAGCTCATATAATGCCACCAAGAATTACTGGATTTGAAGAGCCCTCTGTTATTGCCGCC |
| VLCHR3_4Gln | CCAATACGGATGACGGGGCTCTTCAAATTACTACTGCAGCTCATATCAAGCCACCAAGAATTACTGGATTTGAAGAGCCCTCTGTTATTGCCGCC |
| VLCHR3_4Asp | CCAATACGGATGACGGGGCTCTTCAAATTACTACTGCAGCTCATATGACGCCACCAAGAATTACTGGATTTGAAGAGCCCTCTGTTATTGCCGCC |
| VLCHR3_4Glu | CCAATACGGATGACGGGGCTCTTCAAATTACTACTGCAGCTCATATGAGGCCACCAAGAATTACTGGATTTGAAGAGCCCTCTGTTATTGCCGCC |
| VLCHR3_4Lys | CCAATACGGATGACGGGGCTCTTCAAATTACTACTGCAGCTCATATAAAGCCACCAAGAATTACTGGATTTGAAGAGCCCTCTGTTATTGCCGCC |
| VLCHR3_4Arg | CCAATACGGATGACGGGGCTCTTCAAATTACTACTGCAGCTCATATCGGGCCACCAAGAATTACTGGATTTGAAGAGCCCTCTGTTATTGCCGCC |
| VLCHR3_4His | CCAATACGGATGACGGGGCTCTTCAAATTACTACTGCAGCTCATATCACGCCACCAAGAATTACTGGATTTGAAGAGCCCTCTGTTATTGCCGCC |
| VLCHR3_5Gly | CCAATACGGATGACGGGGCTCTTCATACTACTGCAGCTCATATACAGGCACCAAGAATTACTGGATTTTCTGAAGAGCCCTCTGTTATTGCCGCC |
| VLCHR3_5Val | CCAATACGGATGACGGGGCTCTTCATACTACTGCAGCTCATATACAGTTACCAAGAATTACTGGATTTTCTGAAGAGCCCTCTGTTATTGCCGCC |
| VLCHR3_5Leu | CCAATACGGATGACGGGGCTCTTCATACTACTGCAGCTCATATACACTTACCAAGAATTACTGGATTTTCTGAAGAGCCCTCTGTTATTGCCGCC |
| VLCHR3_5Ile | CCAATACGGATGACGGGGCTCTTCATACTACTGCAGCTCATATACAATCACCAAGAATTACTGGATTTTCTGAAGAGCCCTCTGTTATTGCCGCC |
| VLCHR3_5Pro | CCAATACGGATGACGGGGCTCTTCATACTACTGCAGCTCATATACACCCACCAAGAATTACTGGATTTTCTGAAGAGCCCTCTGTTATTGCCGCC |
| VLCHR3_5Phe | CCAATACGGATGACGGGGCTCTTCATACTACTGCAGCTCATATACATTTACCAAGAATTACTGGATTTTCTGAAGAGCCCTCTGTTATTGCCGCC |
| VLCHR3_5Tyr | CCAATACGGATGACGGGGCTCTTCATACTACTGCAGCTCATATACATATACCAAGAATTACTGGATTTTCTGAAGAGCCCTCTGTTATTGCCGCC |
| VLCHR3_5Trp | CCAATACGGATGACGGGGCTCTTCATACTACTGCAGCTCATATACATGGACCAAGAATTACTGGATTTTCTGAAGAGCCCTCTGTTATTGCCGCC |
| VLCHR3_5Ser | CCAATACGGATGACGGGGCTCTTCATACTACTGCAGCTCATATACAAGTACCAAGAATTACTGGATTTTCTGAAGAGCCCTCTGTTATTGCCGCC |
| VLCHR3_5Thr | CCAATACGGATGACGGGGCTCTTCATACTACTGCAGCTCATATACAACCACCAAGAATTACTGGATTTTCTGAAGAGCCCTCTGTTATTGCCGCC |
| VLCHR3_5Cys | CCAATACGGATGACGGGGCTCTTCATACTACTGCAGCTCATATACATGCACCAAGAATTACTGGATTTTCTGAAGAGCCCTCTGTTATTGCCGCC |
| VLCHR3_5Met | CCAATACGGATGACGGGGCTCTTCATACTACTGCAGCTCATATACAATGACCAAGAATTACTGGATTTTCTGAAGAGCCCTCTGTTATTGCCGCC |
| VLCHR3_5Asn | CCAATACGGATGACGGGGCTCTTCATACTACTGCAGCTCATATACAAACACCAAGAATTACTGGATTTTCTGAAGAGCCCTCTGTTATTGCCGCC |
| VLCHR3_5Gln | CCAATACGGATGACGGGGCTCTTCATACTACTGCAGCTCATATACACAAACCAAGAATTACTGGATTTTCTGAAGAGCCCTCTGTTATTGCCGCC |
| VLCHR3_5Asp | CCAATACGGATGACGGGGCTCTTCATACTACTGCAGCTCATATACAGATACCAAGAATTACTGGATTTTCTGAAGAGCCCTCTGTTATTGCCGCC |
| VLCHR3_5Glu | CCAATACGGATGACGGGGCTCTTCATACTACTGCAGCTCATATACAGAAACCAAGAATTACTGGATTTTCTGAAGAGCCCTCTGTTATTGCCGCC |
| VLCHR3_5Lys | CCAATACGGATGACGGGGCTCTTCATACTACTGCAGCTCATATACAAAAACCAAGAATTACTGGATTTTCTGAAGAGCCCTCTGTTATTGCCGCC |
| VLCHR3_5Arg | CCAATACGGATGACGGGGCTCTTCATACTACTGCAGCTCATATACAAGGACCAAGAATTACTGGATTTTCTGAAGAGCCCTCTGTTATTGCCGCC |
| VLCHR3_5His | CCAATACGGATGACGGGGCTCTTCATACTACTGCAGCTCATATACACATACCAAGAATTACTGGATTTTCTGAAGAGCCCTCTGTTATTGCCGCC |
| VLCHR3_6Gly | CCAATACGGATGACGGGGCTCTTCATACTGCAGCTCATATACAGCCGGAAAGAATTACTGGATTTTCGGCTGAAGAGCCCTCTGTTATTGCCGCC |
| VLCHR3_6Ala | CCAATACGGATGACGGGGCTCTTCATACTGCAGCTCATATACAGCCGCAAAGAATTACTGGATTTTCGGCTGAAGAGCCCTCTGTTATTGCCGCC |
| VLCHR3_6Val | CCAATACGGATGACGGGGCTCTTCATACTGCAGCTCATATACAGCCGTCAAGAATTACTGGATTTTCGGCTGAAGAGCCCTCTGTTATTGCCGCC |
| VLCHR3_6Leu | CCAATACGGATGACGGGGCTCTTCATACTGCAGCTCATATACAGCCCTGAAGAATTACTGGATTTTCGGCTGAAGAGCCCTCTGTTATTGCCGCC |
| VLCHR3_6Ile | CCAATACGGATGACGGGGCTCTTCATACTGCAGCTCATATACAGCCATTAAGAATTACTGGATTTTCGGCTGAAGAGCCCTCTGTTATTGCCGCC |
| VLCHR3_6Pro | CCAATACGGATGACGGGGCTCTTCATACTGCAGCTCATATACAGCCCCTAAGAATTACTGGATTTTCGGCTGAAGAGCCCTCTGTTATTGCCGCC |
| VLCHR3_6Phe | CCAATACGGATGACGGGGCTCTTCATACTGCAGCTCATATACAGCCTTTAAGAATTACTGGATTTTCGGCTGAAGAGCCCTCTGTTATTGCCGCC |
| VLCHR3_6Tyr | CCAATACGGATGACGGGGCTCTTCATACTGCAGCTCATATACAGCCTATAAGAATTACTGGATTTTCGGCTGAAGAGCCCTCTGTTATTGCCGCC |
| VLCHR3_6Trp | CCAATACGGATGACGGGGCTCTTCATACTGCAGCTCATATACAGCCTGGAAGAATTACTGGATTTTCGGCTGAAGAGCCCTCTGTTATTGCCGCC |
| VLCHR3_6Ser | CCAATACGGATGACGGGGCTCTTCATACTGCAGCTCATATACAGCCAGTAAGAATTACTGGATTTTCGGCTGAAGAGCCCTCTGTTATTGCCGCC |
| VLCHR3_6Cys | CCAATACGGATGACGGGGCTCTTCATACTGCAGCTCATATACAGCCTGTAAGAATTACTGGATTTTCGGCTGAAGAGCCCTCTGTTATTGCCGCC |
| VLCHR3_6Met | CCAATACGGATGACGGGGCTCTTCATACTGCAGCTCATATACAGCCATGAAGAATTACTGGATTTTCGGCTGAAGAGCCCTCTGTTATTGCCGCC |
| VLCHR3_6Asn | CCAATACGGATGACGGGGCTCTTCATACTGCAGCTCATATACAGCCAACAAGAATTACTGGATTTTCGGCTGAAGAGCCCTCTGTTATTGCCGCC |
| VLCHR3_6Gln | CCAATACGGATGACGGGGCTCTTCATACTGCAGCTCATATACAGCCCAGAAGAATTACTGGATTTTCGGCTGAAGAGCCCTCTGTTATTGCCGCC |
| VLCHR3_6Asp | CCAATACGGATGACGGGGCTCTTCATACTGCAGCTCATATACAGCCGACAAGAATTACTGGATTTTCGGCTGAAGAGCCCTCTGTTATTGCCGCC |
| VLCHR3_6Glu | CCAATACGGATGACGGGGCTCTTCATACTGCAGCTCATATACAGCCGAAAAGAATTACTGGATTTTCGGCTGAAGAGCCCTCTGTTATTGCCGCC |
| VLCHR3_6Lys | CCAATACGGATGACGGGGCTCTTCATACTGCAGCTCATATACAGCCAAGAAGAATTACTGGATTTTCGGCTGAAGAGCCCTCTGTTATTGCCGCC |
| VLCHR3_6Arg | CCAATACGGATGACGGGGCTCTTCATACTGCAGCTCATATACAGCCCGTAAGAATTACTGGATTTTCGGCTGAAGAGCCCTCTGTTATTGCCGCC |
| VLCHR3_6His | CCAATACGGATGACGGGGCTCTTCATACTGCAGCTCATATACAGCCCACAAGAATTACTGGATTTTCGGCTGAAGAGCCCTCTGTTATTGCCGCC |
| VLCHR3_7Gly | CCAATACGGATGACGGGGCTCTTCATGCAGCTCATATACAGCCACCGGCAATTACTGGATTTTCGGCGGATGAAGAGCCCTCTGTTATTGCCGCC |
| VLCHR3_7Ala | CCAATACGGATGACGGGGCTCTTCATGCAGCTCATATACAGCCACCGCCAATTACTGGATTTTCGGCGGATGAAGAGCCCTCTGTTATTGCCGCC |
| VLCHR3_7Val | CCAATACGGATGACGGGGCTCTTCATGCAGCTCATATACAGCCACCGTTAATTACTGGATTTTCGGCGGATGAAGAGCCCTCTGTTATTGCCGCC |
| VLCHR3_7Leu | CCAATACGGATGACGGGGCTCTTCATGCAGCTCATATACAGCCACCCTAAATTACTGGATTTTCGGCGGATGAAGAGCCCTCTGTTATTGCCGCC |
| VLCHR3_7Ile | CCAATACGGATGACGGGGCTCTTCATGCAGCTCATATACAGCCACCATTAATTACTGGATTTTCGGCGGATGAAGAGCCCTCTGTTATTGCCGCC |
| VLCHR3_7Pro | CCAATACGGATGACGGGGCTCTTCATGCAGCTCATATACAGCCACCCCAAATTACTGGATTTTCGGCGGATGAAGAGCCCTCTGTTATTGCCGCC |
| VLCHR3_7Phe | CCAATACGGATGACGGGGCTCTTCATGCAGCTCATATACAGCCACCTTTAATTACTGGATTTTCGGCGGATGAAGAGCCCTCTGTTATTGCCGCC |
| VLCHR3_7Tyr | CCAATACGGATGACGGGGCTCTTCATGCAGCTCATATACAGCCACCTACAATTACTGGATTTTCGGCGGATGAAGAGCCCTCTGTTATTGCCGCC |
| VLCHR3_7Trp | CCAATACGGATGACGGGGCTCTTCATGCAGCTCATATACAGCCACCTGGAATTACTGGATTTTCGGCGGATGAAGAGCCCTCTGTTATTGCCGCC |
| VLCHR3_7Ser | CCAATACGGATGACGGGGCTCTTCATGCAGCTCATATACAGCCACCTCTAATTACTGGATTTTCGGCGGATGAAGAGCCCTCTGTTATTGCCGCC |
| VLCHR3_7Thr | CCAATACGGATGACGGGGCTCTTCATGCAGCTCATATACAGCCACCACTAATTACTGGATTTTCGGCGGATGAAGAGCCCTCTGTTATTGCCGCC |
| VLCHR3_7Cys | CCAATACGGATGACGGGGCTCTTCATGCAGCTCATATACAGCCACCTGTAATTACTGGATTTTCGGCGGATGAAGAGCCCTCTGTTATTGCCGCC |
| VLCHR3_7Met | CCAATACGGATGACGGGGCTCTTCATGCAGCTCATATACAGCCACCATGAATTACTGGATTTTCGGCGGATGAAGAGCCCTCTGTTATTGCCGCC |
| VLCHR3_7Asn | CCAATACGGATGACGGGGCTCTTCATGCAGCTCATATACAGCCACCAACAATTACTGGATTTTCGGCGGATGAAGAGCCCTCTGTTATTGCCGCC |
| VLCHR3_7Gln | CCAATACGGATGACGGGGCTCTTCATGCAGCTCATATACAGCCACCCAGAATTACTGGATTTTCGGCGGATGAAGAGCCCTCTGTTATTGCCGCC |
| VLCHR3_7Asp | CCAATACGGATGACGGGGCTCTTCATGCAGCTCATATACAGCCACCGACAATTACTGGATTTTCGGCGGATGAAGAGCCCTCTGTTATTGCCGCC |
| VLCHR3_7Glu | CCAATACGGATGACGGGGCTCTTCATGCAGCTCATATACAGCCACCGAGAATTACTGGATTTTCGGCGGATGAAGAGCCCTCTGTTATTGCCGCC |
| VLCHR3_7Arg | CCAATACGGATGACGGGGCTCTTCATGCAGCTCATATACAGCCACCAGGAATTACTGGATTTTCGGCGGATGAAGAGCCCTCTGTTATTGCCGCC |
| VLCHR3_7His | CCAATACGGATGACGGGGCTCTTCATGCAGCTCATATACAGCCACCCACAATTACTGGATTTTCGGCGGATGAAGAGCCCTCTGTTATTGCCGCC |
| VLCHR3_8Gly | CCAATACGGATGACGGGGCTCTTCAAGCTCATATACAGCCACCAAGGGTTACTGGATTTTCGGCGGAGGGTGAAGAGCCCTCTGTTATTGCCGCC |
| VLCHR3_8Ala | CCAATACGGATGACGGGGCTCTTCAAGCTCATATACAGCCACCAAGGCTTACTGGATTTTCGGCGGAGGGTGAAGAGCCCTCTGTTATTGCCGCC |
| VLCHR3_8Val | CCAATACGGATGACGGGGCTCTTCAAGCTCATATACAGCCACCAAGGTCTACTGGATTTTCGGCGGAGGGTGAAGAGCCCTCTGTTATTGCCGCC |
| VLCHR3_8Leu | CCAATACGGATGACGGGGCTCTTCAAGCTCATATACAGCCACCAAGTTGTACTGGATTTTCGGCGGAGGGTGAAGAGCCCTCTGTTATTGCCGCC |
| VLCHR3_8Ile | CCAATACGGATGACGGGGCTCTTCAAGCTCATATACAGCCACCAAGATTTACTGGATTTTCGGCGGAGGGTGAAGAGCCCTCTGTTATTGCCGCC |
| VLCHR3_8Pro | CCAATACGGATGACGGGGCTCTTCAAGCTCATATACAGCCACCAAGCCTTACTGGATTTTCGGCGGAGGGTGAAGAGCCCTCTGTTATTGCCGCC |
| VLCHR3_8Phe | CCAATACGGATGACGGGGCTCTTCAAGCTCATATACAGCCACCAAGTTCTACTGGATTTTCGGCGGAGGGTGAAGAGCCCTCTGTTATTGCCGCC |
| VLCHR3_8Tyr | CCAATACGGATGACGGGGCTCTTCAAGCTCATATACAGCCACCAAGTACTACTGGATTTTCGGCGGAGGGTGAAGAGCCCTCTGTTATTGCCGCC |
| VLCHR3_8Trp | CCAATACGGATGACGGGGCTCTTCAAGCTCATATACAGCCACCAAGTGGTACTGGATTTTCGGCGGAGGGTGAAGAGCCCTCTGTTATTGCCGCC |
| VLCHR3_8Ser | CCAATACGGATGACGGGGCTCTTCAAGCTCATATACAGCCACCAAGTCTTACTGGATTTTCGGCGGAGGGTGAAGAGCCCTCTGTTATTGCCGCC |
| VLCHR3_8Thr | CCAATACGGATGACGGGGCTCTTCAAGCTCATATACAGCCACCAAGACCTACTGGATTTTCGGCGGAGGGTGAAGAGCCCTCTGTTATTGCCGCC |
| VLCHR3_8Cys | CCAATACGGATGACGGGGCTCTTCAAGCTCATATACAGCCACCAAGTGCTACTGGATTTTCGGCGGAGGGTGAAGAGCCCTCTGTTATTGCCGCC |
| VLCHR3_8Met | CCAATACGGATGACGGGGCTCTTCAAGCTCATATACAGCCACCAAGATGTACTGGATTTTCGGCGGAGGGTGAAGAGCCCTCTGTTATTGCCGCC |
| VLCHR3_8Gln | CCAATACGGATGACGGGGCTCTTCAAGCTCATATACAGCCACCAAGCAGTACTGGATTTTCGGCGGAGGGTGAAGAGCCCTCTGTTATTGCCGCC |
| VLCHR3_8Asp | CCAATACGGATGACGGGGCTCTTCAAGCTCATATACAGCCACCAAGGACTACTGGATTTTCGGCGGAGGGTGAAGAGCCCTCTGTTATTGCCGCC |
| VLCHR3_8Glu | CCAATACGGATGACGGGGCTCTTCAAGCTCATATACAGCCACCAAGGAGTACTGGATTTTCGGCGGAGGGTGAAGAGCCCTCTGTTATTGCCGCC |
| VLCHR3_8Lys | CCAATACGGATGACGGGGCTCTTCAAGCTCATATACAGCCACCAAGAAATACTGGATTTTCGGCGGAGGGTGAAGAGCCCTCTGTTATTGCCGCC |
| VLCHR3_8Arg | CCAATACGGATGACGGGGCTCTTCAAGCTCATATACAGCCACCAAGCGCTACTGGATTTTCGGCGGAGGGTGAAGAGCCCTCTGTTATTGCCGCC |
| VLCHR3_8His | CCAATACGGATGACGGGGCTCTTCAAGCTCATATACAGCCACCAAGCACTACTGGATTTTCGGCGGAGGGTGAAGAGCCCTCTGTTATTGCCGCC |
| VLCHR3_9Gly | CCAATACGGATGACGGGGCTCTTCATCATATACAGCCACCAAGAATGGCTGGATTTTCGGCGGAGGGACCTGAAGAGCCCTCTGTTATTGCCGCC |
| VLCHR3_9Ala | CCAATACGGATGACGGGGCTCTTCATCATATACAGCCACCAAGAATGCTTGGATTTTCGGCGGAGGGACCTGAAGAGCCCTCTGTTATTGCCGCC |
| VLCHR3_9Val | CCAATACGGATGACGGGGCTCTTCATCATATACAGCCACCAAGAATGTTTGGATTTTCGGCGGAGGGACCTGAAGAGCCCTCTGTTATTGCCGCC |
| VLCHR3_9Leu | CCAATACGGATGACGGGGCTCTTCATCATATACAGCCACCAAGAATCTCTGGATTTTCGGCGGAGGGACCTGAAGAGCCCTCTGTTATTGCCGCC |
| VLCHR3_9Ile | CCAATACGGATGACGGGGCTCTTCATCATATACAGCCACCAAGAATATTTGGATTTTCGGCGGAGGGACCTGAAGAGCCCTCTGTTATTGCCGCC |
| VLCHR3_9Pro | CCAATACGGATGACGGGGCTCTTCATCATATACAGCCACCAAGAATCCCTGGATTTTCGGCGGAGGGACCTGAAGAGCCCTCTGTTATTGCCGCC |
| VLCHR3_9Phe | CCAATACGGATGACGGGGCTCTTCATCATATACAGCCACCAAGAATTTCTGGATTTTCGGCGGAGGGACCTGAAGAGCCCTCTGTTATTGCCGCC |
| VLCHR3_9Trp | CCAATACGGATGACGGGGCTCTTCATCATATACAGCCACCAAGAATTGGTGGATTTTCGGCGGAGGGACCTGAAGAGCCCTCTGTTATTGCCGCC |
| VLCHR3_9Ser | CCAATACGGATGACGGGGCTCTTCATCATATACAGCCACCAAGAATAGTTGGATTTTCGGCGGAGGGACCTGAAGAGCCCTCTGTTATTGCCGCC |
| VLCHR3_9Thr | CCAATACGGATGACGGGGCTCTTCATCATATACAGCCACCAAGAATACATGGATTTTCGGCGGAGGGACCTGAAGAGCCCTCTGTTATTGCCGCC |
| VLCHR3_9Cys | CCAATACGGATGACGGGGCTCTTCATCATATACAGCCACCAAGAATTGTTGGATTTTCGGCGGAGGGACCTGAAGAGCCCTCTGTTATTGCCGCC |
| VLCHR3_9Met | CCAATACGGATGACGGGGCTCTTCATCATATACAGCCACCAAGAATATGTGGATTTTCGGCGGAGGGACCTGAAGAGCCCTCTGTTATTGCCGCC |
| VLCHR3_9Asn | CCAATACGGATGACGGGGCTCTTCATCATATACAGCCACCAAGAATAATTGGATTTTCGGCGGAGGGACCTGAAGAGCCCTCTGTTATTGCCGCC |
| VLCHR3_9Gln | CCAATACGGATGACGGGGCTCTTCATCATATACAGCCACCAAGAATCAGTGGATTTTCGGCGGAGGGACCTGAAGAGCCCTCTGTTATTGCCGCC |
| VLCHR3_9Asp | CCAATACGGATGACGGGGCTCTTCATCATATACAGCCACCAAGAATGATTGGATTTTCGGCGGAGGGACCTGAAGAGCCCTCTGTTATTGCCGCC |
| VLCHR3_9Glu | CCAATACGGATGACGGGGCTCTTCATCATATACAGCCACCAAGAATGAGTGGATTTTCGGCGGAGGGACCTGAAGAGCCCTCTGTTATTGCCGCC |
| VLCHR3_9Lys | CCAATACGGATGACGGGGCTCTTCATCATATACAGCCACCAAGAATAAGTGGATTTTCGGCGGAGGGACCTGAAGAGCCCTCTGTTATTGCCGCC |
| VLCHR3_9Arg | CCAATACGGATGACGGGGCTCTTCATCATATACAGCCACCAAGAATCGCTGGATTTTCGGCGGAGGGACCTGAAGAGCCCTCTGTTATTGCCGCC |
| VLCHR3_9His | CCAATACGGATGACGGGGCTCTTCATCATATACAGCCACCAAGAATCATTGGATTTTCGGCGGAGGGACCTGAAGAGCCCTCTGTTATTGCCGCC |
| VLCHR3_10Gly | CCAATACGGATGACGGGGCTCTTCATATACAGCCACCAAGAATTACGGTATTTTCGGCGGAGGGACCAAGTGAAGAGCCCTCTGTTATTGCCGCC |
| VLCHR3_10Ala | CCAATACGGATGACGGGGCTCTTCATATACAGCCACCAAGAATTACGCCATTTTCGGCGGAGGGACCAAGTGAAGAGCCCTCTGTTATTGCCGCC |
| VLCHR3_10Val | CCAATACGGATGACGGGGCTCTTCATATACAGCCACCAAGAATTACGTCATTTTCGGCGGAGGGACCAAGTGAAGAGCCCTCTGTTATTGCCGCC |
| VLCHR3_10Leu | CCAATACGGATGACGGGGCTCTTCATATACAGCCACCAAGAATTACCTTATTTTCGGCGGAGGGACCAAGTGAAGAGCCCTCTGTTATTGCCGCC |
| VLCHR3_10Ile | CCAATACGGATGACGGGGCTCTTCATATACAGCCACCAAGAATTACATCATTTTCGGCGGAGGGACCAAGTGAAGAGCCCTCTGTTATTGCCGCC |
| VLCHR3_10Pro | CCAATACGGATGACGGGGCTCTTCATATACAGCCACCAAGAATTACCCAATTTTCGGCGGAGGGACCAAGTGAAGAGCCCTCTGTTATTGCCGCC |
| VLCHR3_10Phe | CCAATACGGATGACGGGGCTCTTCATATACAGCCACCAAGAATTACTTCATTTTCGGCGGAGGGACCAAGTGAAGAGCCCTCTGTTATTGCCGCC |
| VLCHR3_10Tyr | CCAATACGGATGACGGGGCTCTTCATATACAGCCACCAAGAATTACTATATTTTCGGCGGAGGGACCAAGTGAAGAGCCCTCTGTTATTGCCGCC |
| VLCHR3_10Ser | CCAATACGGATGACGGGGCTCTTCATATACAGCCACCAAGAATTACAGCATTTTCGGCGGAGGGACCAAGTGAAGAGCCCTCTGTTATTGCCGCC |
| VLCHR3_10Thr | CCAATACGGATGACGGGGCTCTTCATATACAGCCACCAAGAATTACACAATTTTCGGCGGAGGGACCAAGTGAAGAGCCCTCTGTTATTGCCGCC |
| VLCHR3_10Cys | CCAATACGGATGACGGGGCTCTTCATATACAGCCACCAAGAATTACTGTATTTTCGGCGGAGGGACCAAGTGAAGAGCCCTCTGTTATTGCCGCC |
| VLCHR3_10Met | CCAATACGGATGACGGGGCTCTTCATATACAGCCACCAAGAATTACATGATTTTCGGCGGAGGGACCAAGTGAAGAGCCCTCTGTTATTGCCGCC |
| VLCHR3_10Asn | CCAATACGGATGACGGGGCTCTTCATATACAGCCACCAAGAATTACAATATTTTCGGCGGAGGGACCAAGTGAAGAGCCCTCTGTTATTGCCGCC |
| VLCHR3_10Gln | CCAATACGGATGACGGGGCTCTTCATATACAGCCACCAAGAATTACCAAATTTTCGGCGGAGGGACCAAGTGAAGAGCCCTCTGTTATTGCCGCC |
| VLCHR3_10Asp | CCAATACGGATGACGGGGCTCTTCATATACAGCCACCAAGAATTACGACATTTTCGGCGGAGGGACCAAGTGAAGAGCCCTCTGTTATTGCCGCC |
| VLCHR3_10Glu | CCAATACGGATGACGGGGCTCTTCATATACAGCCACCAAGAATTACGAAATTTTCGGCGGAGGGACCAAGTGAAGAGCCCTCTGTTATTGCCGCC |
| VLCHR3_10Lys | CCAATACGGATGACGGGGCTCTTCATATACAGCCACCAAGAATTACAAAATTTTCGGCGGAGGGACCAAGTGAAGAGCCCTCTGTTATTGCCGCC |
| VLCHR3_10Arg | CCAATACGGATGACGGGGCTCTTCATATACAGCCACCAAGAATTACCGCATTTTCGGCGGAGGGACCAAGTGAAGAGCCCTCTGTTATTGCCGCC |
| VLCHR3_10His | CCAATACGGATGACGGGGCTCTTCATATACAGCCACCAAGAATTACCATATTTTCGGCGGAGGGACCAAGTGAAGAGCCCTCTGTTATTGCCGCC |
| VLCHR3_11Gly | CCAATACGGATGACGGGGCTCTTCAACAGCCACCAAGAATTACTGGGGGTTCGGCGGAGGGACCAAGCTGTGAAGAGCCCTCTGTTATTGCCGCC |
| VLCHR3_11Ala | CCAATACGGATGACGGGGCTCTTCAACAGCCACCAAGAATTACTGGGCTTTCGGCGGAGGGACCAAGCTGTGAAGAGCCCTCTGTTATTGCCGCC |
| VLCHR3_11Val | CCAATACGGATGACGGGGCTCTTCAACAGCCACCAAGAATTACTGGGTCTTCGGCGGAGGGACCAAGCTGTGAAGAGCCCTCTGTTATTGCCGCC |
| VLCHR3_11Leu | CCAATACGGATGACGGGGCTCTTCAACAGCCACCAAGAATTACTGGTTATTCGGCGGAGGGACCAAGCTGTGAAGAGCCCTCTGTTATTGCCGCC |
| VLCHR3_11Pro | CCAATACGGATGACGGGGCTCTTCAACAGCCACCAAGAATTACTGGCCGTTCGGCGGAGGGACCAAGCTGTGAAGAGCCCTCTGTTATTGCCGCC |
| VLCHR3_11Phe | CCAATACGGATGACGGGGCTCTTCAACAGCCACCAAGAATTACTGGTTCTTCGGCGGAGGGACCAAGCTGTGAAGAGCCCTCTGTTATTGCCGCC |
| VLCHR3_11Tyr | CCAATACGGATGACGGGGCTCTTCAACAGCCACCAAGAATTACTGGTACTTCGGCGGAGGGACCAAGCTGTGAAGAGCCCTCTGTTATTGCCGCC |
| VLCHR3_11Trp | CCAATACGGATGACGGGGCTCTTCAACAGCCACCAAGAATTACTGGTGGTTCGGCGGAGGGACCAAGCTGTGAAGAGCCCTCTGTTATTGCCGCC |
| VLCHR3_11Ser | CCAATACGGATGACGGGGCTCTTCAACAGCCACCAAGAATTACTGGAGTTTCGGCGGAGGGACCAAGCTGTGAAGAGCCCTCTGTTATTGCCGCC |
| VLCHR3_11Thr | CCAATACGGATGACGGGGCTCTTCAACAGCCACCAAGAATTACTGGACATTCGGCGGAGGGACCAAGCTGTGAAGAGCCCTCTGTTATTGCCGCC |
| VLCHR3_11Cys | CCAATACGGATGACGGGGCTCTTCAACAGCCACCAAGAATTACTGGTGTTTCGGCGGAGGGACCAAGCTGTGAAGAGCCCTCTGTTATTGCCGCC |
| VLCHR3_11Met | CCAATACGGATGACGGGGCTCTTCAACAGCCACCAAGAATTACTGGATGTTCGGCGGAGGGACCAAGCTGTGAAGAGCCCTCTGTTATTGCCGCC |
| VLCHR3_11Asn | CCAATACGGATGACGGGGCTCTTCAACAGCCACCAAGAATTACTGGAATTTCGGCGGAGGGACCAAGCTGTGAAGAGCCCTCTGTTATTGCCGCC |
| VLCHR3_11Gln | CCAATACGGATGACGGGGCTCTTCAACAGCCACCAAGAATTACTGGCAATTCGGCGGAGGGACCAAGCTGTGAAGAGCCCTCTGTTATTGCCGCC |
| VLCHR3_11Asp | CCAATACGGATGACGGGGCTCTTCAACAGCCACCAAGAATTACTGGGACTTCGGCGGAGGGACCAAGCTGTGAAGAGCCCTCTGTTATTGCCGCC |
| VLCHR3_11Glu | CCAATACGGATGACGGGGCTCTTCAACAGCCACCAAGAATTACTGGGAGTTCGGCGGAGGGACCAAGCTGTGAAGAGCCCTCTGTTATTGCCGCC |
| VLCHR3_11Lys | CCAATACGGATGACGGGGCTCTTCAACAGCCACCAAGAATTACTGGAAATTCGGCGGAGGGACCAAGCTGTGAAGAGCCCTCTGTTATTGCCGCC |
| VLCHR3_11Arg | CCAATACGGATGACGGGGCTCTTCAACAGCCACCAAGAATTACTGGCGCTTCGGCGGAGGGACCAAGCTGTGAAGAGCCCTCTGTTATTGCCGCC |
| VLCHR3_11His | CCAATACGGATGACGGGGCTCTTCAACAGCCACCAAGAATTACTGGCACTTCGGCGGAGGGACCAAGCTGTGAAGAGCCCTCTGTTATTGCCGCC |
| VHCHR1_1Ala | CCAATACGGATGACGGGGCTCTTCATCCCTCACCTGCACTGTCTCTGCTGGCTCCATCAGCAGTGTTAATTGAAGAGCCCTCTGTTATTGCCGCC |
| VHCHR1_1Val | CCAATACGGATGACGGGGCTCTTCATCCCTCACCTGCACTGTCTCTGTTGGCTCCATCAGCAGTGTTAATTGAAGAGCCCTCTGTTATTGCCGCC |
| VHCHR1_1Leu | CCAATACGGATGACGGGGCTCTTCATCCCTCACCTGCACTGTCTCTCTCGGCTCCATCAGCAGTGTTAATTGAAGAGCCCTCTGTTATTGCCGCC |
| VHCHR1_1Ile | CCAATACGGATGACGGGGCTCTTCATCCCTCACCTGCACTGTCTCTATCGGCTCCATCAGCAGTGTTAATTGAAGAGCCCTCTGTTATTGCCGCC |
| VHCHR1_1Pro | CCAATACGGATGACGGGGCTCTTCATCCCTCACCTGCACTGTCTCTCCAGGCTCCATCAGCAGTGTTAATTGAAGAGCCCTCTGTTATTGCCGCC |
| VHCHR1_1Phe | CCAATACGGATGACGGGGCTCTTCATCCCTCACCTGCACTGTCTCTTTTGGCTCCATCAGCAGTGTTAATTGAAGAGCCCTCTGTTATTGCCGCC |
| VHCHR1_1Tyr | CCAATACGGATGACGGGGCTCTTCATCCCTCACCTGCACTGTCTCTTACGGCTCCATCAGCAGTGTTAATTGAAGAGCCCTCTGTTATTGCCGCC |
| VHCHR1_1Trp | CCAATACGGATGACGGGGCTCTTCATCCCTCACCTGCACTGTCTCTTGGGGCTCCATCAGCAGTGTTAATTGAAGAGCCCTCTGTTATTGCCGCC |
| VHCHR1_1Ser | CCAATACGGATGACGGGGCTCTTCATCCCTCACCTGCACTGTCTCTTCAGGCTCCATCAGCAGTGTTAATTGAAGAGCCCTCTGTTATTGCCGCC |
| VHCHR1_1Thr | CCAATACGGATGACGGGGCTCTTCATCCCTCACCTGCACTGTCTCTACGGGCTCCATCAGCAGTGTTAATTGAAGAGCCCTCTGTTATTGCCGCC |
| VHCHR1_1Cys | CCAATACGGATGACGGGGCTCTTCATCCCTCACCTGCACTGTCTCTTGTGGCTCCATCAGCAGTGTTAATTGAAGAGCCCTCTGTTATTGCCGCC |
| VHCHR1_1Met | CCAATACGGATGACGGGGCTCTTCATCCCTCACCTGCACTGTCTCTATGGGCTCCATCAGCAGTGTTAATTGAAGAGCCCTCTGTTATTGCCGCC |
| VHCHR1_1Asn | CCAATACGGATGACGGGGCTCTTCATCCCTCACCTGCACTGTCTCTAATGGCTCCATCAGCAGTGTTAATTGAAGAGCCCTCTGTTATTGCCGCC |
| VHCHR1_1Gln | CCAATACGGATGACGGGGCTCTTCATCCCTCACCTGCACTGTCTCTCAAGGCTCCATCAGCAGTGTTAATTGAAGAGCCCTCTGTTATTGCCGCC |
| VHCHR1_1Asp | CCAATACGGATGACGGGGCTCTTCATCCCTCACCTGCACTGTCTCTGATGGCTCCATCAGCAGTGTTAATTGAAGAGCCCTCTGTTATTGCCGCC |
| VHCHR1_1Glu | CCAATACGGATGACGGGGCTCTTCATCCCTCACCTGCACTGTCTCTGAAGGCTCCATCAGCAGTGTTAATTGAAGAGCCCTCTGTTATTGCCGCC |
| VHCHR1_1Lys | CCAATACGGATGACGGGGCTCTTCATCCCTCACCTGCACTGTCTCTAAGGGCTCCATCAGCAGTGTTAATTGAAGAGCCCTCTGTTATTGCCGCC |
| VHCHR1_1Arg | CCAATACGGATGACGGGGCTCTTCATCCCTCACCTGCACTGTCTCTCGGGGCTCCATCAGCAGTGTTAATTGAAGAGCCCTCTGTTATTGCCGCC |
| VHCHR1_1His | CCAATACGGATGACGGGGCTCTTCATCCCTCACCTGCACTGTCTCTCACGGCTCCATCAGCAGTGTTAATTGAAGAGCCCTCTGTTATTGCCGCC |
| VHCHR1_2Ala | CCAATACGGATGACGGGGCTCTTCACTCACCTGCACTGTCTCTGGTGCCTCCATCAGCAGTGTTAATTCCTGAAGAGCCCTCTGTTATTGCCGCC |
| VHCHR1_2Val | CCAATACGGATGACGGGGCTCTTCACTCACCTGCACTGTCTCTGGTGTATCCATCAGCAGTGTTAATTCCTGAAGAGCCCTCTGTTATTGCCGCC |
| VHCHR1_2Leu | CCAATACGGATGACGGGGCTCTTCACTCACCTGCACTGTCTCTGGTCTTTCCATCAGCAGTGTTAATTCCTGAAGAGCCCTCTGTTATTGCCGCC |
| VHCHR1_2Ile | CCAATACGGATGACGGGGCTCTTCACTCACCTGCACTGTCTCTGGTATATCCATCAGCAGTGTTAATTCCTGAAGAGCCCTCTGTTATTGCCGCC |
| VHCHR1_2Pro | CCAATACGGATGACGGGGCTCTTCACTCACCTGCACTGTCTCTGGTCCGTCCATCAGCAGTGTTAATTCCTGAAGAGCCCTCTGTTATTGCCGCC |
| VHCHR1_2Phe | CCAATACGGATGACGGGGCTCTTCACTCACCTGCACTGTCTCTGGTTTCTCCATCAGCAGTGTTAATTCCTGAAGAGCCCTCTGTTATTGCCGCC |
| VHCHR1_2Tyr | CCAATACGGATGACGGGGCTCTTCACTCACCTGCACTGTCTCTGGTTATTCCATCAGCAGTGTTAATTCCTGAAGAGCCCTCTGTTATTGCCGCC |
| VHCHR1_2Trp | CCAATACGGATGACGGGGCTCTTCACTCACCTGCACTGTCTCTGGTTGGTCCATCAGCAGTGTTAATTCCTGAAGAGCCCTCTGTTATTGCCGCC |
| VHCHR1_2Ser | CCAATACGGATGACGGGGCTCTTCACTCACCTGCACTGTCTCTGGTTCATCCATCAGCAGTGTTAATTCCTGAAGAGCCCTCTGTTATTGCCGCC |
| VHCHR1_2Thr | CCAATACGGATGACGGGGCTCTTCACTCACCTGCACTGTCTCTGGTACTTCCATCAGCAGTGTTAATTCCTGAAGAGCCCTCTGTTATTGCCGCC |
| VHCHR1_2Cys | CCAATACGGATGACGGGGCTCTTCACTCACCTGCACTGTCTCTGGTTGTTCCATCAGCAGTGTTAATTCCTGAAGAGCCCTCTGTTATTGCCGCC |
| VHCHR1_2Met | CCAATACGGATGACGGGGCTCTTCACTCACCTGCACTGTCTCTGGTATGTCCATCAGCAGTGTTAATTCCTGAAGAGCCCTCTGTTATTGCCGCC |
| VHCHR1_2Asn | CCAATACGGATGACGGGGCTCTTCACTCACCTGCACTGTCTCTGGTAATTCCATCAGCAGTGTTAATTCCTGAAGAGCCCTCTGTTATTGCCGCC |
| VHCHR1_2Gln | CCAATACGGATGACGGGGCTCTTCACTCACCTGCACTGTCTCTGGTCAATCCATCAGCAGTGTTAATTCCTGAAGAGCCCTCTGTTATTGCCGCC |
| VHCHR1_2Asp | CCAATACGGATGACGGGGCTCTTCACTCACCTGCACTGTCTCTGGTGACTCCATCAGCAGTGTTAATTCCTGAAGAGCCCTCTGTTATTGCCGCC |
| VHCHR1_2Glu | CCAATACGGATGACGGGGCTCTTCACTCACCTGCACTGTCTCTGGTGAATCCATCAGCAGTGTTAATTCCTGAAGAGCCCTCTGTTATTGCCGCC |
| VHCHR1_2Lys | CCAATACGGATGACGGGGCTCTTCACTCACCTGCACTGTCTCTGGTAAGTCCATCAGCAGTGTTAATTCCTGAAGAGCCCTCTGTTATTGCCGCC |
| VHCHR1_2Arg | CCAATACGGATGACGGGGCTCTTCACTCACCTGCACTGTCTCTGGTCGTTCCATCAGCAGTGTTAATTCCTGAAGAGCCCTCTGTTATTGCCGCC |
| VHCHR1_2His | CCAATACGGATGACGGGGCTCTTCACTCACCTGCACTGTCTCTGGTCATTCCATCAGCAGTGTTAATTCCTGAAGAGCCCTCTGTTATTGCCGCC |
| VHCHR1_3Gly | CCAATACGGATGACGGGGCTCTTCAACCTGCACTGTCTCTGGTGGCGGAATCAGCAGTGTTAATTCCTACTGAAGAGCCCTCTGTTATTGCCGCC |
| VHCHR1_3Ala | CCAATACGGATGACGGGGCTCTTCAACCTGCACTGTCTCTGGTGGCGCCATCAGCAGTGTTAATTCCTACTGAAGAGCCCTCTGTTATTGCCGCC |
| VHCHR1_3Val | CCAATACGGATGACGGGGCTCTTCAACCTGCACTGTCTCTGGTGGCGTAATCAGCAGTGTTAATTCCTACTGAAGAGCCCTCTGTTATTGCCGCC |
| VHCHR1_3Leu | CCAATACGGATGACGGGGCTCTTCAACCTGCACTGTCTCTGGTGGCCTCATCAGCAGTGTTAATTCCTACTGAAGAGCCCTCTGTTATTGCCGCC |
| VHCHR1_3Ile | CCAATACGGATGACGGGGCTCTTCAACCTGCACTGTCTCTGGTGGCATAATCAGCAGTGTTAATTCCTACTGAAGAGCCCTCTGTTATTGCCGCC |
| VHCHR1_3Pro | CCAATACGGATGACGGGGCTCTTCAACCTGCACTGTCTCTGGTGGCCCCATCAGCAGTGTTAATTCCTACTGAAGAGCCCTCTGTTATTGCCGCC |
| VHCHR1_3Phe | CCAATACGGATGACGGGGCTCTTCAACCTGCACTGTCTCTGGTGGCTTCATCAGCAGTGTTAATTCCTACTGAAGAGCCCTCTGTTATTGCCGCC |
| VHCHR1_3Tyr | CCAATACGGATGACGGGGCTCTTCAACCTGCACTGTCTCTGGTGGCTATATCAGCAGTGTTAATTCCTACTGAAGAGCCCTCTGTTATTGCCGCC |
| VHCHR1_3Trp | CCAATACGGATGACGGGGCTCTTCAACCTGCACTGTCTCTGGTGGCTGGATCAGCAGTGTTAATTCCTACTGAAGAGCCCTCTGTTATTGCCGCC |
| VHCHR1_3Thr | CCAATACGGATGACGGGGCTCTTCAACCTGCACTGTCTCTGGTGGCACAATCAGCAGTGTTAATTCCTACTGAAGAGCCCTCTGTTATTGCCGCC |
| VHCHR1_3Cys | CCAATACGGATGACGGGGCTCTTCAACCTGCACTGTCTCTGGTGGCTGTATCAGCAGTGTTAATTCCTACTGAAGAGCCCTCTGTTATTGCCGCC |
| VHCHR1_3Met | CCAATACGGATGACGGGGCTCTTCAACCTGCACTGTCTCTGGTGGCATGATCAGCAGTGTTAATTCCTACTGAAGAGCCCTCTGTTATTGCCGCC |
| VHCHR1_3Asn | CCAATACGGATGACGGGGCTCTTCAACCTGCACTGTCTCTGGTGGCAACATCAGCAGTGTTAATTCCTACTGAAGAGCCCTCTGTTATTGCCGCC |
| VHCHR1_3Gln | CCAATACGGATGACGGGGCTCTTCAACCTGCACTGTCTCTGGTGGCCAGATCAGCAGTGTTAATTCCTACTGAAGAGCCCTCTGTTATTGCCGCC |
| VHCHR1_3Asp | CCAATACGGATGACGGGGCTCTTCAACCTGCACTGTCTCTGGTGGCGATATCAGCAGTGTTAATTCCTACTGAAGAGCCCTCTGTTATTGCCGCC |
| VHCHR1_3Glu | CCAATACGGATGACGGGGCTCTTCAACCTGCACTGTCTCTGGTGGCGAGATCAGCAGTGTTAATTCCTACTGAAGAGCCCTCTGTTATTGCCGCC |
| VHCHR1_3Lys | CCAATACGGATGACGGGGCTCTTCAACCTGCACTGTCTCTGGTGGCAAGATCAGCAGTGTTAATTCCTACTGAAGAGCCCTCTGTTATTGCCGCC |
| VHCHR1_3Arg | CCAATACGGATGACGGGGCTCTTCAACCTGCACTGTCTCTGGTGGCAGAATCAGCAGTGTTAATTCCTACTGAAGAGCCCTCTGTTATTGCCGCC |
| VHCHR1_3His | CCAATACGGATGACGGGGCTCTTCAACCTGCACTGTCTCTGGTGGCCACATCAGCAGTGTTAATTCCTACTGAAGAGCCCTCTGTTATTGCCGCC |
| VHCHR1_4Gly | CCAATACGGATGACGGGGCTCTTCATGCACTGTCTCTGGTGGCTCCGGAAGCAGTGTTAATTCCTACTGGTGAAGAGCCCTCTGTTATTGCCGCC |
| VHCHR1_4Ala | CCAATACGGATGACGGGGCTCTTCATGCACTGTCTCTGGTGGCTCCGCCAGCAGTGTTAATTCCTACTGGTGAAGAGCCCTCTGTTATTGCCGCC |
| VHCHR1_4Val | CCAATACGGATGACGGGGCTCTTCATGCACTGTCTCTGGTGGCTCCGTAAGCAGTGTTAATTCCTACTGGTGAAGAGCCCTCTGTTATTGCCGCC |
| VHCHR1_4Leu | CCAATACGGATGACGGGGCTCTTCATGCACTGTCTCTGGTGGCTCCTTGAGCAGTGTTAATTCCTACTGGTGAAGAGCCCTCTGTTATTGCCGCC |
| VHCHR1_4Pro | CCAATACGGATGACGGGGCTCTTCATGCACTGTCTCTGGTGGCTCCCCAAGCAGTGTTAATTCCTACTGGTGAAGAGCCCTCTGTTATTGCCGCC |
| VHCHR1_4Phe | CCAATACGGATGACGGGGCTCTTCATGCACTGTCTCTGGTGGCTCCTTCAGCAGTGTTAATTCCTACTGGTGAAGAGCCCTCTGTTATTGCCGCC |
| VHCHR1_4Tyr | CCAATACGGATGACGGGGCTCTTCATGCACTGTCTCTGGTGGCTCCTATAGCAGTGTTAATTCCTACTGGTGAAGAGCCCTCTGTTATTGCCGCC |
| VHCHR1_4Trp | CCAATACGGATGACGGGGCTCTTCATGCACTGTCTCTGGTGGCTCCTGGAGCAGTGTTAATTCCTACTGGTGAAGAGCCCTCTGTTATTGCCGCC |
| VHCHR1_4Ser | CCAATACGGATGACGGGGCTCTTCATGCACTGTCTCTGGTGGCTCCAGCAGCAGTGTTAATTCCTACTGGTGAAGAGCCCTCTGTTATTGCCGCC |
| VHCHR1_4Thr | CCAATACGGATGACGGGGCTCTTCATGCACTGTCTCTGGTGGCTCCACTAGCAGTGTTAATTCCTACTGGTGAAGAGCCCTCTGTTATTGCCGCC |
| VHCHR1_4Cys | CCAATACGGATGACGGGGCTCTTCATGCACTGTCTCTGGTGGCTCCTGCAGCAGTGTTAATTCCTACTGGTGAAGAGCCCTCTGTTATTGCCGCC |
| VHCHR1_4Met | CCAATACGGATGACGGGGCTCTTCATGCACTGTCTCTGGTGGCTCCATGAGCAGTGTTAATTCCTACTGGTGAAGAGCCCTCTGTTATTGCCGCC |
| VHCHR1_4Asn | CCAATACGGATGACGGGGCTCTTCATGCACTGTCTCTGGTGGCTCCAACAGCAGTGTTAATTCCTACTGGTGAAGAGCCCTCTGTTATTGCCGCC |
| VHCHR1_4Gln | CCAATACGGATGACGGGGCTCTTCATGCACTGTCTCTGGTGGCTCCCAAAGCAGTGTTAATTCCTACTGGTGAAGAGCCCTCTGTTATTGCCGCC |
| VHCHR1_4Asp | CCAATACGGATGACGGGGCTCTTCATGCACTGTCTCTGGTGGCTCCGACAGCAGTGTTAATTCCTACTGGTGAAGAGCCCTCTGTTATTGCCGCC |
| VHCHR1_4Glu | CCAATACGGATGACGGGGCTCTTCATGCACTGTCTCTGGTGGCTCCGAAAGCAGTGTTAATTCCTACTGGTGAAGAGCCCTCTGTTATTGCCGCC |
| VHCHR1_4Lys | CCAATACGGATGACGGGGCTCTTCATGCACTGTCTCTGGTGGCTCCAAGAGCAGTGTTAATTCCTACTGGTGAAGAGCCCTCTGTTATTGCCGCC |
| VHCHR1_4Arg | CCAATACGGATGACGGGGCTCTTCATGCACTGTCTCTGGTGGCTCCCGGAGCAGTGTTAATTCCTACTGGTGAAGAGCCCTCTGTTATTGCCGCC |
| VHCHR1_4His | CCAATACGGATGACGGGGCTCTTCATGCACTGTCTCTGGTGGCTCCCATAGCAGTGTTAATTCCTACTGGTGAAGAGCCCTCTGTTATTGCCGCC |
| VHCHR1_5Gly | CCAATACGGATGACGGGGCTCTTCAACTGTCTCTGGTGGCTCCATCGGAAGTGTTAATTCCTACTGGGGCTGAAGAGCCCTCTGTTATTGCCGCC |
| VHCHR1_5Ala | CCAATACGGATGACGGGGCTCTTCAACTGTCTCTGGTGGCTCCATCGCGAGTGTTAATTCCTACTGGGGCTGAAGAGCCCTCTGTTATTGCCGCC |
| VHCHR1_5Val | CCAATACGGATGACGGGGCTCTTCAACTGTCTCTGGTGGCTCCATCGTAAGTGTTAATTCCTACTGGGGCTGAAGAGCCCTCTGTTATTGCCGCC |
| VHCHR1_5Leu | CCAATACGGATGACGGGGCTCTTCAACTGTCTCTGGTGGCTCCATCCTCAGTGTTAATTCCTACTGGGGCTGAAGAGCCCTCTGTTATTGCCGCC |
| VHCHR1_5Ile | CCAATACGGATGACGGGGCTCTTCAACTGTCTCTGGTGGCTCCATCATTAGTGTTAATTCCTACTGGGGCTGAAGAGCCCTCTGTTATTGCCGCC |
| VHCHR1_5Pro | CCAATACGGATGACGGGGCTCTTCAACTGTCTCTGGTGGCTCCATCCCGAGTGTTAATTCCTACTGGGGCTGAAGAGCCCTCTGTTATTGCCGCC |
| VHCHR1_5Phe | CCAATACGGATGACGGGGCTCTTCAACTGTCTCTGGTGGCTCCATCTTCAGTGTTAATTCCTACTGGGGCTGAAGAGCCCTCTGTTATTGCCGCC |
| VHCHR1_5Tyr | CCAATACGGATGACGGGGCTCTTCAACTGTCTCTGGTGGCTCCATCTATAGTGTTAATTCCTACTGGGGCTGAAGAGCCCTCTGTTATTGCCGCC |
| VHCHR1_5Trp | CCAATACGGATGACGGGGCTCTTCAACTGTCTCTGGTGGCTCCATCTGGAGTGTTAATTCCTACTGGGGCTGAAGAGCCCTCTGTTATTGCCGCC |
| VHCHR1_5Thr | CCAATACGGATGACGGGGCTCTTCAACTGTCTCTGGTGGCTCCATCACTAGTGTTAATTCCTACTGGGGCTGAAGAGCCCTCTGTTATTGCCGCC |
| VHCHR1_5Cys | CCAATACGGATGACGGGGCTCTTCAACTGTCTCTGGTGGCTCCATCTGTAGTGTTAATTCCTACTGGGGCTGAAGAGCCCTCTGTTATTGCCGCC |
| VHCHR1_5Met | CCAATACGGATGACGGGGCTCTTCAACTGTCTCTGGTGGCTCCATCATGAGTGTTAATTCCTACTGGGGCTGAAGAGCCCTCTGTTATTGCCGCC |
| VHCHR1_5Asn | CCAATACGGATGACGGGGCTCTTCAACTGTCTCTGGTGGCTCCATCAACAGTGTTAATTCCTACTGGGGCTGAAGAGCCCTCTGTTATTGCCGCC |
| VHCHR1_5Gln | CCAATACGGATGACGGGGCTCTTCAACTGTCTCTGGTGGCTCCATCCAAAGTGTTAATTCCTACTGGGGCTGAAGAGCCCTCTGTTATTGCCGCC |
| VHCHR1_5Asp | CCAATACGGATGACGGGGCTCTTCAACTGTCTCTGGTGGCTCCATCGACAGTGTTAATTCCTACTGGGGCTGAAGAGCCCTCTGTTATTGCCGCC |
| VHCHR1_5Glu | CCAATACGGATGACGGGGCTCTTCAACTGTCTCTGGTGGCTCCATCGAGAGTGTTAATTCCTACTGGGGCTGAAGAGCCCTCTGTTATTGCCGCC |
| VHCHR1_5Lys | CCAATACGGATGACGGGGCTCTTCAACTGTCTCTGGTGGCTCCATCAAGAGTGTTAATTCCTACTGGGGCTGAAGAGCCCTCTGTTATTGCCGCC |
| VHCHR1_5Arg | CCAATACGGATGACGGGGCTCTTCAACTGTCTCTGGTGGCTCCATCAGAAGTGTTAATTCCTACTGGGGCTGAAGAGCCCTCTGTTATTGCCGCC |
| VHCHR1_5His | CCAATACGGATGACGGGGCTCTTCAACTGTCTCTGGTGGCTCCATCCATAGTGTTAATTCCTACTGGGGCTGAAGAGCCCTCTGTTATTGCCGCC |
| VHCHR1_6Gly | CCAATACGGATGACGGGGCTCTTCAGTCTCTGGTGGCTCCATCAGCGGTGTTAATTCCTACTGGGGCTGGTGAAGAGCCCTCTGTTATTGCCGCC |
| VHCHR1_6Ala | CCAATACGGATGACGGGGCTCTTCAGTCTCTGGTGGCTCCATCAGCGCGGTTAATTCCTACTGGGGCTGGTGAAGAGCCCTCTGTTATTGCCGCC |
| VHCHR1_6Val | CCAATACGGATGACGGGGCTCTTCAGTCTCTGGTGGCTCCATCAGCGTTGTTAATTCCTACTGGGGCTGGTGAAGAGCCCTCTGTTATTGCCGCC |
| VHCHR1_6Leu | CCAATACGGATGACGGGGCTCTTCAGTCTCTGGTGGCTCCATCAGCTTGGTTAATTCCTACTGGGGCTGGTGAAGAGCCCTCTGTTATTGCCGCC |
| VHCHR1_6Ile | CCAATACGGATGACGGGGCTCTTCAGTCTCTGGTGGCTCCATCAGCATTGTTAATTCCTACTGGGGCTGGTGAAGAGCCCTCTGTTATTGCCGCC |
| VHCHR1_6Pro | CCAATACGGATGACGGGGCTCTTCAGTCTCTGGTGGCTCCATCAGCCCAGTTAATTCCTACTGGGGCTGGTGAAGAGCCCTCTGTTATTGCCGCC |
| VHCHR1_6Phe | CCAATACGGATGACGGGGCTCTTCAGTCTCTGGTGGCTCCATCAGCTTTGTTAATTCCTACTGGGGCTGGTGAAGAGCCCTCTGTTATTGCCGCC |
| VHCHR1_6Tyr | CCAATACGGATGACGGGGCTCTTCAGTCTCTGGTGGCTCCATCAGCTATGTTAATTCCTACTGGGGCTGGTGAAGAGCCCTCTGTTATTGCCGCC |
| VHCHR1_6Trp | CCAATACGGATGACGGGGCTCTTCAGTCTCTGGTGGCTCCATCAGCTGGGTTAATTCCTACTGGGGCTGGTGAAGAGCCCTCTGTTATTGCCGCC |
| VHCHR1_6Thr | CCAATACGGATGACGGGGCTCTTCAGTCTCTGGTGGCTCCATCAGCACGGTTAATTCCTACTGGGGCTGGTGAAGAGCCCTCTGTTATTGCCGCC |
| VHCHR1_6Cys | CCAATACGGATGACGGGGCTCTTCAGTCTCTGGTGGCTCCATCAGCTGCGTTAATTCCTACTGGGGCTGGTGAAGAGCCCTCTGTTATTGCCGCC |
| VHCHR1_6Met | CCAATACGGATGACGGGGCTCTTCAGTCTCTGGTGGCTCCATCAGCATGGTTAATTCCTACTGGGGCTGGTGAAGAGCCCTCTGTTATTGCCGCC |
| VHCHR1_6Asn | CCAATACGGATGACGGGGCTCTTCAGTCTCTGGTGGCTCCATCAGCAACGTTAATTCCTACTGGGGCTGGTGAAGAGCCCTCTGTTATTGCCGCC |
| VHCHR1_6Gln | CCAATACGGATGACGGGGCTCTTCAGTCTCTGGTGGCTCCATCAGCCAGGTTAATTCCTACTGGGGCTGGTGAAGAGCCCTCTGTTATTGCCGCC |
| VHCHR1_6Asp | CCAATACGGATGACGGGGCTCTTCAGTCTCTGGTGGCTCCATCAGCGATGTTAATTCCTACTGGGGCTGGTGAAGAGCCCTCTGTTATTGCCGCC |
| VHCHR1_6Glu | CCAATACGGATGACGGGGCTCTTCAGTCTCTGGTGGCTCCATCAGCGAGGTTAATTCCTACTGGGGCTGGTGAAGAGCCCTCTGTTATTGCCGCC |
| VHCHR1_6Lys | CCAATACGGATGACGGGGCTCTTCAGTCTCTGGTGGCTCCATCAGCAAAGTTAATTCCTACTGGGGCTGGTGAAGAGCCCTCTGTTATTGCCGCC |
| VHCHR1_6Arg | CCAATACGGATGACGGGGCTCTTCAGTCTCTGGTGGCTCCATCAGCCGAGTTAATTCCTACTGGGGCTGGTGAAGAGCCCTCTGTTATTGCCGCC |
| VHCHR1_6His | CCAATACGGATGACGGGGCTCTTCAGTCTCTGGTGGCTCCATCAGCCACGTTAATTCCTACTGGGGCTGGTGAAGAGCCCTCTGTTATTGCCGCC |
| VHCHR1_7Gly | CCAATACGGATGACGGGGCTCTTCATCTGGTGGCTCCATCAGCAGTGGGAATTCCTACTGGGGCTGGATCTGAAGAGCCCTCTGTTATTGCCGCC |
| VHCHR1_7Ala | CCAATACGGATGACGGGGCTCTTCATCTGGTGGCTCCATCAGCAGTGCTAATTCCTACTGGGGCTGGATCTGAAGAGCCCTCTGTTATTGCCGCC |
| VHCHR1_7Leu | CCAATACGGATGACGGGGCTCTTCATCTGGTGGCTCCATCAGCAGTTTGAATTCCTACTGGGGCTGGATCTGAAGAGCCCTCTGTTATTGCCGCC |
| VHCHR1_7Ile | CCAATACGGATGACGGGGCTCTTCATCTGGTGGCTCCATCAGCAGTATCAATTCCTACTGGGGCTGGATCTGAAGAGCCCTCTGTTATTGCCGCC |
| VHCHR1_7Pro | CCAATACGGATGACGGGGCTCTTCATCTGGTGGCTCCATCAGCAGTCCTAATTCCTACTGGGGCTGGATCTGAAGAGCCCTCTGTTATTGCCGCC |
| VHCHR1_7Phe | CCAATACGGATGACGGGGCTCTTCATCTGGTGGCTCCATCAGCAGTTTCAATTCCTACTGGGGCTGGATCTGAAGAGCCCTCTGTTATTGCCGCC |
| VHCHR1_7Tyr | CCAATACGGATGACGGGGCTCTTCATCTGGTGGCTCCATCAGCAGTTATAATTCCTACTGGGGCTGGATCTGAAGAGCCCTCTGTTATTGCCGCC |
| VHCHR1_7Trp | CCAATACGGATGACGGGGCTCTTCATCTGGTGGCTCCATCAGCAGTTGGAATTCCTACTGGGGCTGGATCTGAAGAGCCCTCTGTTATTGCCGCC |
| VHCHR1_7Ser | CCAATACGGATGACGGGGCTCTTCATCTGGTGGCTCCATCAGCAGTTCTAATTCCTACTGGGGCTGGATCTGAAGAGCCCTCTGTTATTGCCGCC |
| VHCHR1_7Thr | CCAATACGGATGACGGGGCTCTTCATCTGGTGGCTCCATCAGCAGTACAAATTCCTACTGGGGCTGGATCTGAAGAGCCCTCTGTTATTGCCGCC |
| VHCHR1_7Cys | CCAATACGGATGACGGGGCTCTTCATCTGGTGGCTCCATCAGCAGTTGTAATTCCTACTGGGGCTGGATCTGAAGAGCCCTCTGTTATTGCCGCC |
| VHCHR1_7Met | CCAATACGGATGACGGGGCTCTTCATCTGGTGGCTCCATCAGCAGTATGAATTCCTACTGGGGCTGGATCTGAAGAGCCCTCTGTTATTGCCGCC |
| VHCHR1_7Asn | CCAATACGGATGACGGGGCTCTTCATCTGGTGGCTCCATCAGCAGTAACAATTCCTACTGGGGCTGGATCTGAAGAGCCCTCTGTTATTGCCGCC |
| VHCHR1_7Gln | CCAATACGGATGACGGGGCTCTTCATCTGGTGGCTCCATCAGCAGTCAGAATTCCTACTGGGGCTGGATCTGAAGAGCCCTCTGTTATTGCCGCC |
| VHCHR1_7Asp | CCAATACGGATGACGGGGCTCTTCATCTGGTGGCTCCATCAGCAGTGACAATTCCTACTGGGGCTGGATCTGAAGAGCCCTCTGTTATTGCCGCC |
| VHCHR1_7Glu | CCAATACGGATGACGGGGCTCTTCATCTGGTGGCTCCATCAGCAGTGAGAATTCCTACTGGGGCTGGATCTGAAGAGCCCTCTGTTATTGCCGCC |
| VHCHR1_7Lys | CCAATACGGATGACGGGGCTCTTCATCTGGTGGCTCCATCAGCAGTAAAAATTCCTACTGGGGCTGGATCTGAAGAGCCCTCTGTTATTGCCGCC |
| VHCHR1_7Arg | CCAATACGGATGACGGGGCTCTTCATCTGGTGGCTCCATCAGCAGTCGTAATTCCTACTGGGGCTGGATCTGAAGAGCCCTCTGTTATTGCCGCC |
| VHCHR1_7His | CCAATACGGATGACGGGGCTCTTCATCTGGTGGCTCCATCAGCAGTCACAATTCCTACTGGGGCTGGATCTGAAGAGCCCTCTGTTATTGCCGCC |
| VHCHR1_8Gly | CCAATACGGATGACGGGGCTCTTCAGGTGGCTCCATCAGCAGTGTTGGATCCTACTGGGGCTGGATCCGCTGAAGAGCCCTCTGTTATTGCCGCC |
| VHCHR1_8Ala | CCAATACGGATGACGGGGCTCTTCAGGTGGCTCCATCAGCAGTGTTGCGTCCTACTGGGGCTGGATCCGCTGAAGAGCCCTCTGTTATTGCCGCC |
| VHCHR1_8Val | CCAATACGGATGACGGGGCTCTTCAGGTGGCTCCATCAGCAGTGTTGTCTCCTACTGGGGCTGGATCCGCTGAAGAGCCCTCTGTTATTGCCGCC |
| VHCHR1_8Leu | CCAATACGGATGACGGGGCTCTTCAGGTGGCTCCATCAGCAGTGTTCTCTCCTACTGGGGCTGGATCCGCTGAAGAGCCCTCTGTTATTGCCGCC |
| VHCHR1_8Ile | CCAATACGGATGACGGGGCTCTTCAGGTGGCTCCATCAGCAGTGTTATCTCCTACTGGGGCTGGATCCGCTGAAGAGCCCTCTGTTATTGCCGCC |
| VHCHR1_8Pro | CCAATACGGATGACGGGGCTCTTCAGGTGGCTCCATCAGCAGTGTTCCGTCCTACTGGGGCTGGATCCGCTGAAGAGCCCTCTGTTATTGCCGCC |
| VHCHR1_8Phe | CCAATACGGATGACGGGGCTCTTCAGGTGGCTCCATCAGCAGTGTTTTTTCCTACTGGGGCTGGATCCGCTGAAGAGCCCTCTGTTATTGCCGCC |
| VHCHR1_8Tyr | CCAATACGGATGACGGGGCTCTTCAGGTGGCTCCATCAGCAGTGTTTACTCCTACTGGGGCTGGATCCGCTGAAGAGCCCTCTGTTATTGCCGCC |
| VHCHR1_8Trp | CCAATACGGATGACGGGGCTCTTCAGGTGGCTCCATCAGCAGTGTTTGGTCCTACTGGGGCTGGATCCGCTGAAGAGCCCTCTGTTATTGCCGCC |
| VHCHR1_8Ser | CCAATACGGATGACGGGGCTCTTCAGGTGGCTCCATCAGCAGTGTTAGCTCCTACTGGGGCTGGATCCGCTGAAGAGCCCTCTGTTATTGCCGCC |
| VHCHR1_8Thr | CCAATACGGATGACGGGGCTCTTCAGGTGGCTCCATCAGCAGTGTTACCTCCTACTGGGGCTGGATCCGCTGAAGAGCCCTCTGTTATTGCCGCC |
| VHCHR1_8Cys | CCAATACGGATGACGGGGCTCTTCAGGTGGCTCCATCAGCAGTGTTTGCTCCTACTGGGGCTGGATCCGCTGAAGAGCCCTCTGTTATTGCCGCC |
| VHCHR1_8Met | CCAATACGGATGACGGGGCTCTTCAGGTGGCTCCATCAGCAGTGTTATGTCCTACTGGGGCTGGATCCGCTGAAGAGCCCTCTGTTATTGCCGCC |
| VHCHR1_8Gln | CCAATACGGATGACGGGGCTCTTCAGGTGGCTCCATCAGCAGTGTTCAGTCCTACTGGGGCTGGATCCGCTGAAGAGCCCTCTGTTATTGCCGCC |
| VHCHR1_8Asp | CCAATACGGATGACGGGGCTCTTCAGGTGGCTCCATCAGCAGTGTTGACTCCTACTGGGGCTGGATCCGCTGAAGAGCCCTCTGTTATTGCCGCC |
| VHCHR1_8Glu | CCAATACGGATGACGGGGCTCTTCAGGTGGCTCCATCAGCAGTGTTGAGTCCTACTGGGGCTGGATCCGCTGAAGAGCCCTCTGTTATTGCCGCC |
| VHCHR1_8Lys | CCAATACGGATGACGGGGCTCTTCAGGTGGCTCCATCAGCAGTGTTAAATCCTACTGGGGCTGGATCCGCTGAAGAGCCCTCTGTTATTGCCGCC |
| VHCHR1_8Arg | CCAATACGGATGACGGGGCTCTTCAGGTGGCTCCATCAGCAGTGTTCGGTCCTACTGGGGCTGGATCCGCTGAAGAGCCCTCTGTTATTGCCGCC |
| VHCHR1_8His | CCAATACGGATGACGGGGCTCTTCAGGTGGCTCCATCAGCAGTGTTCATTCCTACTGGGGCTGGATCCGCTGAAGAGCCCTCTGTTATTGCCGCC |
| VHCHR1_9Gly | CCAATACGGATGACGGGGCTCTTCAGGCTCCATCAGCAGTGTTAATGGTTACTGGGGCTGGATCCGCCAGTGAAGAGCCCTCTGTTATTGCCGCC |
| VHCHR1_9Ala | CCAATACGGATGACGGGGCTCTTCAGGCTCCATCAGCAGTGTTAATGCGTACTGGGGCTGGATCCGCCAGTGAAGAGCCCTCTGTTATTGCCGCC |
| VHCHR1_9Val | CCAATACGGATGACGGGGCTCTTCAGGCTCCATCAGCAGTGTTAATGTCTACTGGGGCTGGATCCGCCAGTGAAGAGCCCTCTGTTATTGCCGCC |
| VHCHR1_9Leu | CCAATACGGATGACGGGGCTCTTCAGGCTCCATCAGCAGTGTTAATCTTTACTGGGGCTGGATCCGCCAGTGAAGAGCCCTCTGTTATTGCCGCC |
| VHCHR1_9Ile | CCAATACGGATGACGGGGCTCTTCAGGCTCCATCAGCAGTGTTAATATTTACTGGGGCTGGATCCGCCAGTGAAGAGCCCTCTGTTATTGCCGCC |
| VHCHR1_9Pro | CCAATACGGATGACGGGGCTCTTCAGGCTCCATCAGCAGTGTTAATCCATACTGGGGCTGGATCCGCCAGTGAAGAGCCCTCTGTTATTGCCGCC |
| VHCHR1_9Phe | CCAATACGGATGACGGGGCTCTTCAGGCTCCATCAGCAGTGTTAATTTTTACTGGGGCTGGATCCGCCAGTGAAGAGCCCTCTGTTATTGCCGCC |
| VHCHR1_9Tyr | CCAATACGGATGACGGGGCTCTTCAGGCTCCATCAGCAGTGTTAATTATTACTGGGGCTGGATCCGCCAGTGAAGAGCCCTCTGTTATTGCCGCC |
| VHCHR1_9Trp | CCAATACGGATGACGGGGCTCTTCAGGCTCCATCAGCAGTGTTAATTGGTACTGGGGCTGGATCCGCCAGTGAAGAGCCCTCTGTTATTGCCGCC |
| VHCHR1_9Thr | CCAATACGGATGACGGGGCTCTTCAGGCTCCATCAGCAGTGTTAATACCTACTGGGGCTGGATCCGCCAGTGAAGAGCCCTCTGTTATTGCCGCC |
| VHCHR1_9Cys | CCAATACGGATGACGGGGCTCTTCAGGCTCCATCAGCAGTGTTAATTGCTACTGGGGCTGGATCCGCCAGTGAAGAGCCCTCTGTTATTGCCGCC |
| VHCHR1_9Met | CCAATACGGATGACGGGGCTCTTCAGGCTCCATCAGCAGTGTTAATATGTACTGGGGCTGGATCCGCCAGTGAAGAGCCCTCTGTTATTGCCGCC |
| VHCHR1_9Asn | CCAATACGGATGACGGGGCTCTTCAGGCTCCATCAGCAGTGTTAATAACTACTGGGGCTGGATCCGCCAGTGAAGAGCCCTCTGTTATTGCCGCC |
| VHCHR1_9Gln | CCAATACGGATGACGGGGCTCTTCAGGCTCCATCAGCAGTGTTAATCAGTACTGGGGCTGGATCCGCCAGTGAAGAGCCCTCTGTTATTGCCGCC |
| VHCHR1_9Asp | CCAATACGGATGACGGGGCTCTTCAGGCTCCATCAGCAGTGTTAATGATTACTGGGGCTGGATCCGCCAGTGAAGAGCCCTCTGTTATTGCCGCC |
| VHCHR1_9Glu | CCAATACGGATGACGGGGCTCTTCAGGCTCCATCAGCAGTGTTAATGAGTACTGGGGCTGGATCCGCCAGTGAAGAGCCCTCTGTTATTGCCGCC |
| VHCHR1_9Lys | CCAATACGGATGACGGGGCTCTTCAGGCTCCATCAGCAGTGTTAATAAATACTGGGGCTGGATCCGCCAGTGAAGAGCCCTCTGTTATTGCCGCC |
| VHCHR1_9Arg | CCAATACGGATGACGGGGCTCTTCAGGCTCCATCAGCAGTGTTAATCGGTACTGGGGCTGGATCCGCCAGTGAAGAGCCCTCTGTTATTGCCGCC |
| VHCHR1_9His | CCAATACGGATGACGGGGCTCTTCAGGCTCCATCAGCAGTGTTAATCACTACTGGGGCTGGATCCGCCAGTGAAGAGCCCTCTGTTATTGCCGCC |
| VHCHR1_10Gly | CCAATACGGATGACGGGGCTCTTCATCCATCAGCAGTGTTAATTCCGGTTGGGGCTGGATCCGCCAGCCCTGAAGAGCCCTCTGTTATTGCCGCC |
| VHCHR1_10Ala | CCAATACGGATGACGGGGCTCTTCATCCATCAGCAGTGTTAATTCCGCTTGGGGCTGGATCCGCCAGCCCTGAAGAGCCCTCTGTTATTGCCGCC |
| VHCHR1_10Val | CCAATACGGATGACGGGGCTCTTCATCCATCAGCAGTGTTAATTCCGTCTGGGGCTGGATCCGCCAGCCCTGAAGAGCCCTCTGTTATTGCCGCC |
| VHCHR1_10Leu | CCAATACGGATGACGGGGCTCTTCATCCATCAGCAGTGTTAATTCCTTGTGGGGCTGGATCCGCCAGCCCTGAAGAGCCCTCTGTTATTGCCGCC |
| VHCHR1_10Ile | CCAATACGGATGACGGGGCTCTTCATCCATCAGCAGTGTTAATTCCATTTGGGGCTGGATCCGCCAGCCCTGAAGAGCCCTCTGTTATTGCCGCC |
| VHCHR1_10Pro | CCAATACGGATGACGGGGCTCTTCATCCATCAGCAGTGTTAATTCCCCATGGGGCTGGATCCGCCAGCCCTGAAGAGCCCTCTGTTATTGCCGCC |
| VHCHR1_10Phe | CCAATACGGATGACGGGGCTCTTCATCCATCAGCAGTGTTAATTCCTTTTGGGGCTGGATCCGCCAGCCCTGAAGAGCCCTCTGTTATTGCCGCC |
| VHCHR1_10Trp | CCAATACGGATGACGGGGCTCTTCATCCATCAGCAGTGTTAATTCCTGGTGGGGCTGGATCCGCCAGCCCTGAAGAGCCCTCTGTTATTGCCGCC |
| VHCHR1_10Ser | CCAATACGGATGACGGGGCTCTTCATCCATCAGCAGTGTTAATTCCTCATGGGGCTGGATCCGCCAGCCCTGAAGAGCCCTCTGTTATTGCCGCC |
| VHCHR1_10Thr | CCAATACGGATGACGGGGCTCTTCATCCATCAGCAGTGTTAATTCCACTTGGGGCTGGATCCGCCAGCCCTGAAGAGCCCTCTGTTATTGCCGCC |
| VHCHR1_10Cys | CCAATACGGATGACGGGGCTCTTCATCCATCAGCAGTGTTAATTCCTGCTGGGGCTGGATCCGCCAGCCCTGAAGAGCCCTCTGTTATTGCCGCC |
| VHCHR1_10Met | CCAATACGGATGACGGGGCTCTTCATCCATCAGCAGTGTTAATTCCATGTGGGGCTGGATCCGCCAGCCCTGAAGAGCCCTCTGTTATTGCCGCC |
| VHCHR1_10Asn | CCAATACGGATGACGGGGCTCTTCATCCATCAGCAGTGTTAATTCCAATTGGGGCTGGATCCGCCAGCCCTGAAGAGCCCTCTGTTATTGCCGCC |
| VHCHR1_10Gln | CCAATACGGATGACGGGGCTCTTCATCCATCAGCAGTGTTAATTCCCAATGGGGCTGGATCCGCCAGCCCTGAAGAGCCCTCTGTTATTGCCGCC |
| VHCHR1_10Asp | CCAATACGGATGACGGGGCTCTTCATCCATCAGCAGTGTTAATTCCGATTGGGGCTGGATCCGCCAGCCCTGAAGAGCCCTCTGTTATTGCCGCC |
| VHCHR1_10Glu | CCAATACGGATGACGGGGCTCTTCATCCATCAGCAGTGTTAATTCCGAATGGGGCTGGATCCGCCAGCCCTGAAGAGCCCTCTGTTATTGCCGCC |
| VHCHR1_10Lys | CCAATACGGATGACGGGGCTCTTCATCCATCAGCAGTGTTAATTCCAAGTGGGGCTGGATCCGCCAGCCCTGAAGAGCCCTCTGTTATTGCCGCC |
| VHCHR1_10Arg | CCAATACGGATGACGGGGCTCTTCATCCATCAGCAGTGTTAATTCCCGTTGGGGCTGGATCCGCCAGCCCTGAAGAGCCCTCTGTTATTGCCGCC |
| VHCHR1_10His | CCAATACGGATGACGGGGCTCTTCATCCATCAGCAGTGTTAATTCCCATTGGGGCTGGATCCGCCAGCCCTGAAGAGCCCTCTGTTATTGCCGCC |
| VHCHR1_11Gly | CCAATACGGATGACGGGGCTCTTCAATCAGCAGTGTTAATTCCTACGGCGGCTGGATCCGCCAGCCCCCATGAAGAGCCCTCTGTTATTGCCGCC |
| VHCHR1_11Ala | CCAATACGGATGACGGGGCTCTTCAATCAGCAGTGTTAATTCCTACGCCGGCTGGATCCGCCAGCCCCCATGAAGAGCCCTCTGTTATTGCCGCC |
| VHCHR1_11Val | CCAATACGGATGACGGGGCTCTTCAATCAGCAGTGTTAATTCCTACGTTGGCTGGATCCGCCAGCCCCCATGAAGAGCCCTCTGTTATTGCCGCC |
| VHCHR1_11Leu | CCAATACGGATGACGGGGCTCTTCAATCAGCAGTGTTAATTCCTACCTGGGCTGGATCCGCCAGCCCCCATGAAGAGCCCTCTGTTATTGCCGCC |
| VHCHR1_11Ile | CCAATACGGATGACGGGGCTCTTCAATCAGCAGTGTTAATTCCTACATTGGCTGGATCCGCCAGCCCCCATGAAGAGCCCTCTGTTATTGCCGCC |
| VHCHR1_11Pro | CCAATACGGATGACGGGGCTCTTCAATCAGCAGTGTTAATTCCTACCCCGGCTGGATCCGCCAGCCCCCATGAAGAGCCCTCTGTTATTGCCGCC |
| VHCHR1_11Phe | CCAATACGGATGACGGGGCTCTTCAATCAGCAGTGTTAATTCCTACTTTGGCTGGATCCGCCAGCCCCCATGAAGAGCCCTCTGTTATTGCCGCC |
| VHCHR1_11Tyr | CCAATACGGATGACGGGGCTCTTCAATCAGCAGTGTTAATTCCTACTACGGCTGGATCCGCCAGCCCCCATGAAGAGCCCTCTGTTATTGCCGCC |
| VHCHR1_11Ser | CCAATACGGATGACGGGGCTCTTCAATCAGCAGTGTTAATTCCTACAGCGGCTGGATCCGCCAGCCCCCATGAAGAGCCCTCTGTTATTGCCGCC |
| VHCHR1_11Thr | CCAATACGGATGACGGGGCTCTTCAATCAGCAGTGTTAATTCCTACACGGGCTGGATCCGCCAGCCCCCATGAAGAGCCCTCTGTTATTGCCGCC |
| VHCHR1_11Cys | CCAATACGGATGACGGGGCTCTTCAATCAGCAGTGTTAATTCCTACTGTGGCTGGATCCGCCAGCCCCCATGAAGAGCCCTCTGTTATTGCCGCC |
| VHCHR1_11Met | CCAATACGGATGACGGGGCTCTTCAATCAGCAGTGTTAATTCCTACATGGGCTGGATCCGCCAGCCCCCATGAAGAGCCCTCTGTTATTGCCGCC |
| VHCHR1_11Asn | CCAATACGGATGACGGGGCTCTTCAATCAGCAGTGTTAATTCCTACAATGGCTGGATCCGCCAGCCCCCATGAAGAGCCCTCTGTTATTGCCGCC |
| VHCHR1_11Gln | CCAATACGGATGACGGGGCTCTTCAATCAGCAGTGTTAATTCCTACCAGGGCTGGATCCGCCAGCCCCCATGAAGAGCCCTCTGTTATTGCCGCC |
| VHCHR1_11Asp | CCAATACGGATGACGGGGCTCTTCAATCAGCAGTGTTAATTCCTACGACGGCTGGATCCGCCAGCCCCCATGAAGAGCCCTCTGTTATTGCCGCC |
| VHCHR1_11Glu | CCAATACGGATGACGGGGCTCTTCAATCAGCAGTGTTAATTCCTACGAAGGCTGGATCCGCCAGCCCCCATGAAGAGCCCTCTGTTATTGCCGCC |
| VHCHR1_11Lys | CCAATACGGATGACGGGGCTCTTCAATCAGCAGTGTTAATTCCTACAAAGGCTGGATCCGCCAGCCCCCATGAAGAGCCCTCTGTTATTGCCGCC |
| VHCHR1_11Arg | CCAATACGGATGACGGGGCTCTTCAATCAGCAGTGTTAATTCCTACCGAGGCTGGATCCGCCAGCCCCCATGAAGAGCCCTCTGTTATTGCCGCC |
| VHCHR1_11His | CCAATACGGATGACGGGGCTCTTCAATCAGCAGTGTTAATTCCTACCACGGCTGGATCCGCCAGCCCCCATGAAGAGCCCTCTGTTATTGCCGCC |
| VHCHR1_12Ala | CCAATACGGATGACGGGGCTCTTCAAGCAGTGTTAATTCCTACTGGGCCTGGATCCGCCAGCCCCCAGGGTGAAGAGCCCTCTGTTATTGCCGCC |
| VHCHR1_12Val | CCAATACGGATGACGGGGCTCTTCAAGCAGTGTTAATTCCTACTGGGTGTGGATCCGCCAGCCCCCAGGGTGAAGAGCCCTCTGTTATTGCCGCC |
| VHCHR1_12Leu | CCAATACGGATGACGGGGCTCTTCAAGCAGTGTTAATTCCTACTGGCTTTGGATCCGCCAGCCCCCAGGGTGAAGAGCCCTCTGTTATTGCCGCC |
| VHCHR1_12Ile | CCAATACGGATGACGGGGCTCTTCAAGCAGTGTTAATTCCTACTGGATATGGATCCGCCAGCCCCCAGGGTGAAGAGCCCTCTGTTATTGCCGCC |
| VHCHR1_12Pro | CCAATACGGATGACGGGGCTCTTCAAGCAGTGTTAATTCCTACTGGCCCTGGATCCGCCAGCCCCCAGGGTGAAGAGCCCTCTGTTATTGCCGCC |
| VHCHR1_12Phe | CCAATACGGATGACGGGGCTCTTCAAGCAGTGTTAATTCCTACTGGTTCTGGATCCGCCAGCCCCCAGGGTGAAGAGCCCTCTGTTATTGCCGCC |
| VHCHR1_12Tyr | CCAATACGGATGACGGGGCTCTTCAAGCAGTGTTAATTCCTACTGGTACTGGATCCGCCAGCCCCCAGGGTGAAGAGCCCTCTGTTATTGCCGCC |
| VHCHR1_12Trp | CCAATACGGATGACGGGGCTCTTCAAGCAGTGTTAATTCCTACTGGTGGTGGATCCGCCAGCCCCCAGGGTGAAGAGCCCTCTGTTATTGCCGCC |
| VHCHR1_12Ser | CCAATACGGATGACGGGGCTCTTCAAGCAGTGTTAATTCCTACTGGAGCTGGATCCGCCAGCCCCCAGGGTGAAGAGCCCTCTGTTATTGCCGCC |
| VHCHR1_12Thr | CCAATACGGATGACGGGGCTCTTCAAGCAGTGTTAATTCCTACTGGACATGGATCCGCCAGCCCCCAGGGTGAAGAGCCCTCTGTTATTGCCGCC |
| VHCHR1_12Cys | CCAATACGGATGACGGGGCTCTTCAAGCAGTGTTAATTCCTACTGGTGCTGGATCCGCCAGCCCCCAGGGTGAAGAGCCCTCTGTTATTGCCGCC |
| VHCHR1_12Met | CCAATACGGATGACGGGGCTCTTCAAGCAGTGTTAATTCCTACTGGATGTGGATCCGCCAGCCCCCAGGGTGAAGAGCCCTCTGTTATTGCCGCC |
| VHCHR1_12Asn | CCAATACGGATGACGGGGCTCTTCAAGCAGTGTTAATTCCTACTGGAACTGGATCCGCCAGCCCCCAGGGTGAAGAGCCCTCTGTTATTGCCGCC |
| VHCHR1_12Gln | CCAATACGGATGACGGGGCTCTTCAAGCAGTGTTAATTCCTACTGGCAGTGGATCCGCCAGCCCCCAGGGTGAAGAGCCCTCTGTTATTGCCGCC |
| VHCHR1_12Asp | CCAATACGGATGACGGGGCTCTTCAAGCAGTGTTAATTCCTACTGGGATTGGATCCGCCAGCCCCCAGGGTGAAGAGCCCTCTGTTATTGCCGCC |
| VHCHR1_12Glu | CCAATACGGATGACGGGGCTCTTCAAGCAGTGTTAATTCCTACTGGGAGTGGATCCGCCAGCCCCCAGGGTGAAGAGCCCTCTGTTATTGCCGCC |
| VHCHR1_12Lys | CCAATACGGATGACGGGGCTCTTCAAGCAGTGTTAATTCCTACTGGAAATGGATCCGCCAGCCCCCAGGGTGAAGAGCCCTCTGTTATTGCCGCC |
| VHCHR1_12Arg | CCAATACGGATGACGGGGCTCTTCAAGCAGTGTTAATTCCTACTGGCGCTGGATCCGCCAGCCCCCAGGGTGAAGAGCCCTCTGTTATTGCCGCC |
| VHCHR1_12His | CCAATACGGATGACGGGGCTCTTCAAGCAGTGTTAATTCCTACTGGCACTGGATCCGCCAGCCCCCAGGGTGAAGAGCCCTCTGTTATTGCCGCC |
| VHCHR2_1Gly | CCAATACGGATGACGGGGCTCTTCAAAGGGGCTGGAGTGGATTGGGGGTTTCTATTATAGTGGGAACACCTGAAGAGCCCTCTGTTATTGCCGCC |
| VHCHR2_1Ala | CCAATACGGATGACGGGGCTCTTCAAAGGGGCTGGAGTGGATTGGGGCTTTCTATTATAGTGGGAACACCTGAAGAGCCCTCTGTTATTGCCGCC |
| VHCHR2_1Val | CCAATACGGATGACGGGGCTCTTCAAAGGGGCTGGAGTGGATTGGGGTGTTCTATTATAGTGGGAACACCTGAAGAGCCCTCTGTTATTGCCGCC |
| VHCHR2_1Leu | CCAATACGGATGACGGGGCTCTTCAAAGGGGCTGGAGTGGATTGGGCTTTTCTATTATAGTGGGAACACCTGAAGAGCCCTCTGTTATTGCCGCC |
| VHCHR2_1Ile | CCAATACGGATGACGGGGCTCTTCAAAGGGGCTGGAGTGGATTGGGATATTCTATTATAGTGGGAACACCTGAAGAGCCCTCTGTTATTGCCGCC |
| VHCHR2_1Pro | CCAATACGGATGACGGGGCTCTTCAAAGGGGCTGGAGTGGATTGGGCCGTTCTATTATAGTGGGAACACCTGAAGAGCCCTCTGTTATTGCCGCC |
| VHCHR2_1Phe | CCAATACGGATGACGGGGCTCTTCAAAGGGGCTGGAGTGGATTGGGTTCTTCTATTATAGTGGGAACACCTGAAGAGCCCTCTGTTATTGCCGCC |
| VHCHR2_1Tyr | CCAATACGGATGACGGGGCTCTTCAAAGGGGCTGGAGTGGATTGGGTATTTCTATTATAGTGGGAACACCTGAAGAGCCCTCTGTTATTGCCGCC |
| VHCHR2_1Trp | CCAATACGGATGACGGGGCTCTTCAAAGGGGCTGGAGTGGATTGGGTGGTTCTATTATAGTGGGAACACCTGAAGAGCCCTCTGTTATTGCCGCC |
| VHCHR2_1Ser | CCAATACGGATGACGGGGCTCTTCAAAGGGGCTGGAGTGGATTGGGAGTTTCTATTATAGTGGGAACACCTGAAGAGCCCTCTGTTATTGCCGCC |
| VHCHR2_1Thr | CCAATACGGATGACGGGGCTCTTCAAAGGGGCTGGAGTGGATTGGGACATTCTATTATAGTGGGAACACCTGAAGAGCCCTCTGTTATTGCCGCC |
| VHCHR2_1Cys | CCAATACGGATGACGGGGCTCTTCAAAGGGGCTGGAGTGGATTGGGTGTTTCTATTATAGTGGGAACACCTGAAGAGCCCTCTGTTATTGCCGCC |
| VHCHR2_1Met | CCAATACGGATGACGGGGCTCTTCAAAGGGGCTGGAGTGGATTGGGATGTTCTATTATAGTGGGAACACCTGAAGAGCCCTCTGTTATTGCCGCC |
| VHCHR2_1Gln | CCAATACGGATGACGGGGCTCTTCAAAGGGGCTGGAGTGGATTGGGCAATTCTATTATAGTGGGAACACCTGAAGAGCCCTCTGTTATTGCCGCC |
| VHCHR2_1Asp | CCAATACGGATGACGGGGCTCTTCAAAGGGGCTGGAGTGGATTGGGGATTTCTATTATAGTGGGAACACCTGAAGAGCCCTCTGTTATTGCCGCC |
| VHCHR2_1Glu | CCAATACGGATGACGGGGCTCTTCAAAGGGGCTGGAGTGGATTGGGGAATTCTATTATAGTGGGAACACCTGAAGAGCCCTCTGTTATTGCCGCC |
| VHCHR2_1Lys | CCAATACGGATGACGGGGCTCTTCAAAGGGGCTGGAGTGGATTGGGAAATTCTATTATAGTGGGAACACCTGAAGAGCCCTCTGTTATTGCCGCC |
| VHCHR2_1Arg | CCAATACGGATGACGGGGCTCTTCAAAGGGGCTGGAGTGGATTGGGCGATTCTATTATAGTGGGAACACCTGAAGAGCCCTCTGTTATTGCCGCC |
| VHCHR2_1His | CCAATACGGATGACGGGGCTCTTCAAAGGGGCTGGAGTGGATTGGGCACTTCTATTATAGTGGGAACACCTGAAGAGCCCTCTGTTATTGCCGCC |
| VHCHR2_2Gly | CCAATACGGATGACGGGGCTCTTCAGGGCTGGAGTGGATTGGGAATGGTTATTATAGTGGGAACACCCACTGAAGAGCCCTCTGTTATTGCCGCC |
| VHCHR2_2Ala | CCAATACGGATGACGGGGCTCTTCAGGGCTGGAGTGGATTGGGAATGCATATTATAGTGGGAACACCCACTGAAGAGCCCTCTGTTATTGCCGCC |
| VHCHR2_2Val | CCAATACGGATGACGGGGCTCTTCAGGGCTGGAGTGGATTGGGAATGTCTATTATAGTGGGAACACCCACTGAAGAGCCCTCTGTTATTGCCGCC |
| VHCHR2_2Leu | CCAATACGGATGACGGGGCTCTTCAGGGCTGGAGTGGATTGGGAATCTGTATTATAGTGGGAACACCCACTGAAGAGCCCTCTGTTATTGCCGCC |
| VHCHR2_2Ile | CCAATACGGATGACGGGGCTCTTCAGGGCTGGAGTGGATTGGGAATATATATTATAGTGGGAACACCCACTGAAGAGCCCTCTGTTATTGCCGCC |
| VHCHR2_2Pro | CCAATACGGATGACGGGGCTCTTCAGGGCTGGAGTGGATTGGGAATCCCTATTATAGTGGGAACACCCACTGAAGAGCCCTCTGTTATTGCCGCC |
| VHCHR2_2Tyr | CCAATACGGATGACGGGGCTCTTCAGGGCTGGAGTGGATTGGGAATTACTATTATAGTGGGAACACCCACTGAAGAGCCCTCTGTTATTGCCGCC |
| VHCHR2_2Trp | CCAATACGGATGACGGGGCTCTTCAGGGCTGGAGTGGATTGGGAATTGGTATTATAGTGGGAACACCCACTGAAGAGCCCTCTGTTATTGCCGCC |
| VHCHR2_2Ser | CCAATACGGATGACGGGGCTCTTCAGGGCTGGAGTGGATTGGGAATAGCTATTATAGTGGGAACACCCACTGAAGAGCCCTCTGTTATTGCCGCC |
| VHCHR2_2Thr | CCAATACGGATGACGGGGCTCTTCAGGGCTGGAGTGGATTGGGAATACCTATTATAGTGGGAACACCCACTGAAGAGCCCTCTGTTATTGCCGCC |
| VHCHR2_2Cys | CCAATACGGATGACGGGGCTCTTCAGGGCTGGAGTGGATTGGGAATTGTTATTATAGTGGGAACACCCACTGAAGAGCCCTCTGTTATTGCCGCC |
| VHCHR2_2Met | CCAATACGGATGACGGGGCTCTTCAGGGCTGGAGTGGATTGGGAATATGTATTATAGTGGGAACACCCACTGAAGAGCCCTCTGTTATTGCCGCC |
| VHCHR2_2Asn | CCAATACGGATGACGGGGCTCTTCAGGGCTGGAGTGGATTGGGAATAACTATTATAGTGGGAACACCCACTGAAGAGCCCTCTGTTATTGCCGCC |
| VHCHR2_2Gln | CCAATACGGATGACGGGGCTCTTCAGGGCTGGAGTGGATTGGGAATCAGTATTATAGTGGGAACACCCACTGAAGAGCCCTCTGTTATTGCCGCC |
| VHCHR2_2Asp | CCAATACGGATGACGGGGCTCTTCAGGGCTGGAGTGGATTGGGAATGATTATTATAGTGGGAACACCCACTGAAGAGCCCTCTGTTATTGCCGCC |
| VHCHR2_2Glu | CCAATACGGATGACGGGGCTCTTCAGGGCTGGAGTGGATTGGGAATGAATATTATAGTGGGAACACCCACTGAAGAGCCCTCTGTTATTGCCGCC |
| VHCHR2_2Lys | CCAATACGGATGACGGGGCTCTTCAGGGCTGGAGTGGATTGGGAATAAATATTATAGTGGGAACACCCACTGAAGAGCCCTCTGTTATTGCCGCC |
| VHCHR2_2Arg | CCAATACGGATGACGGGGCTCTTCAGGGCTGGAGTGGATTGGGAATCGTTATTATAGTGGGAACACCCACTGAAGAGCCCTCTGTTATTGCCGCC |
| VHCHR2_2His | CCAATACGGATGACGGGGCTCTTCAGGGCTGGAGTGGATTGGGAATCACTATTATAGTGGGAACACCCACTGAAGAGCCCTCTGTTATTGCCGCC |
| VHCHR2_3Gly | CCAATACGGATGACGGGGCTCTTCACTGGAGTGGATTGGGAATTTCGGCTATAGTGGGAACACCCACTACTGAAGAGCCCTCTGTTATTGCCGCC |
| VHCHR2_3Ala | CCAATACGGATGACGGGGCTCTTCACTGGAGTGGATTGGGAATTTCGCCTATAGTGGGAACACCCACTACTGAAGAGCCCTCTGTTATTGCCGCC |
| VHCHR2_3Val | CCAATACGGATGACGGGGCTCTTCACTGGAGTGGATTGGGAATTTCGTGTATAGTGGGAACACCCACTACTGAAGAGCCCTCTGTTATTGCCGCC |
| VHCHR2_3Leu | CCAATACGGATGACGGGGCTCTTCACTGGAGTGGATTGGGAATTTCTTGTATAGTGGGAACACCCACTACTGAAGAGCCCTCTGTTATTGCCGCC |
| VHCHR2_3Ile | CCAATACGGATGACGGGGCTCTTCACTGGAGTGGATTGGGAATTTCATCTATAGTGGGAACACCCACTACTGAAGAGCCCTCTGTTATTGCCGCC |
| VHCHR2_3Pro | CCAATACGGATGACGGGGCTCTTCACTGGAGTGGATTGGGAATTTCCCCTATAGTGGGAACACCCACTACTGAAGAGCCCTCTGTTATTGCCGCC |
| VHCHR2_3Phe | CCAATACGGATGACGGGGCTCTTCACTGGAGTGGATTGGGAATTTCTTTTATAGTGGGAACACCCACTACTGAAGAGCCCTCTGTTATTGCCGCC |
| VHCHR2_3Trp | CCAATACGGATGACGGGGCTCTTCACTGGAGTGGATTGGGAATTTCTGGTATAGTGGGAACACCCACTACTGAAGAGCCCTCTGTTATTGCCGCC |
| VHCHR2_3Ser | CCAATACGGATGACGGGGCTCTTCACTGGAGTGGATTGGGAATTTCTCCTATAGTGGGAACACCCACTACTGAAGAGCCCTCTGTTATTGCCGCC |
| VHCHR2_3Thr | CCAATACGGATGACGGGGCTCTTCACTGGAGTGGATTGGGAATTTCACCTATAGTGGGAACACCCACTACTGAAGAGCCCTCTGTTATTGCCGCC |
| VHCHR2_3Cys | CCAATACGGATGACGGGGCTCTTCACTGGAGTGGATTGGGAATTTCTGTTATAGTGGGAACACCCACTACTGAAGAGCCCTCTGTTATTGCCGCC |
| VHCHR2_3Met | CCAATACGGATGACGGGGCTCTTCACTGGAGTGGATTGGGAATTTCATGTATAGTGGGAACACCCACTACTGAAGAGCCCTCTGTTATTGCCGCC |
| VHCHR2_3Asn | CCAATACGGATGACGGGGCTCTTCACTGGAGTGGATTGGGAATTTCAATTATAGTGGGAACACCCACTACTGAAGAGCCCTCTGTTATTGCCGCC |
| VHCHR2_3Gln | CCAATACGGATGACGGGGCTCTTCACTGGAGTGGATTGGGAATTTCCAGTATAGTGGGAACACCCACTACTGAAGAGCCCTCTGTTATTGCCGCC |
| VHCHR2_3Asp | CCAATACGGATGACGGGGCTCTTCACTGGAGTGGATTGGGAATTTCGACTATAGTGGGAACACCCACTACTGAAGAGCCCTCTGTTATTGCCGCC |
| VHCHR2_3Glu | CCAATACGGATGACGGGGCTCTTCACTGGAGTGGATTGGGAATTTCGAATATAGTGGGAACACCCACTACTGAAGAGCCCTCTGTTATTGCCGCC |
| VHCHR2_3Lys | CCAATACGGATGACGGGGCTCTTCACTGGAGTGGATTGGGAATTTCAAGTATAGTGGGAACACCCACTACTGAAGAGCCCTCTGTTATTGCCGCC |
| VHCHR2_3Arg | CCAATACGGATGACGGGGCTCTTCACTGGAGTGGATTGGGAATTTCCGCTATAGTGGGAACACCCACTACTGAAGAGCCCTCTGTTATTGCCGCC |
| VHCHR2_3His | CCAATACGGATGACGGGGCTCTTCACTGGAGTGGATTGGGAATTTCCACTATAGTGGGAACACCCACTACTGAAGAGCCCTCTGTTATTGCCGCC |
| VHCHR2_4Gly | CCAATACGGATGACGGGGCTCTTCAGAGTGGATTGGGAATTTCTATGGCAGTGGGAACACCCACTACAACTGAAGAGCCCTCTGTTATTGCCGCC |
| VHCHR2_4Ala | CCAATACGGATGACGGGGCTCTTCAGAGTGGATTGGGAATTTCTATGCGAGTGGGAACACCCACTACAACTGAAGAGCCCTCTGTTATTGCCGCC |
| VHCHR2_4Val | CCAATACGGATGACGGGGCTCTTCAGAGTGGATTGGGAATTTCTATGTCAGTGGGAACACCCACTACAACTGAAGAGCCCTCTGTTATTGCCGCC |
| VHCHR2_4Leu | CCAATACGGATGACGGGGCTCTTCAGAGTGGATTGGGAATTTCTATTTAAGTGGGAACACCCACTACAACTGAAGAGCCCTCTGTTATTGCCGCC |
| VHCHR2_4Ile | CCAATACGGATGACGGGGCTCTTCAGAGTGGATTGGGAATTTCTATATAAGTGGGAACACCCACTACAACTGAAGAGCCCTCTGTTATTGCCGCC |
| VHCHR2_4Pro | CCAATACGGATGACGGGGCTCTTCAGAGTGGATTGGGAATTTCTATCCTAGTGGGAACACCCACTACAACTGAAGAGCCCTCTGTTATTGCCGCC |
| VHCHR2_4Phe | CCAATACGGATGACGGGGCTCTTCAGAGTGGATTGGGAATTTCTATTTTAGTGGGAACACCCACTACAACTGAAGAGCCCTCTGTTATTGCCGCC |
| VHCHR2_4Trp | CCAATACGGATGACGGGGCTCTTCAGAGTGGATTGGGAATTTCTATTGGAGTGGGAACACCCACTACAACTGAAGAGCCCTCTGTTATTGCCGCC |
| VHCHR2_4Ser | CCAATACGGATGACGGGGCTCTTCAGAGTGGATTGGGAATTTCTATAGTAGTGGGAACACCCACTACAACTGAAGAGCCCTCTGTTATTGCCGCC |
| VHCHR2_4Thr | CCAATACGGATGACGGGGCTCTTCAGAGTGGATTGGGAATTTCTATACTAGTGGGAACACCCACTACAACTGAAGAGCCCTCTGTTATTGCCGCC |
| VHCHR2_4Cys | CCAATACGGATGACGGGGCTCTTCAGAGTGGATTGGGAATTTCTATTGCAGTGGGAACACCCACTACAACTGAAGAGCCCTCTGTTATTGCCGCC |
| VHCHR2_4Met | CCAATACGGATGACGGGGCTCTTCAGAGTGGATTGGGAATTTCTATATGAGTGGGAACACCCACTACAACTGAAGAGCCCTCTGTTATTGCCGCC |
| VHCHR2_4Asn | CCAATACGGATGACGGGGCTCTTCAGAGTGGATTGGGAATTTCTATAATAGTGGGAACACCCACTACAACTGAAGAGCCCTCTGTTATTGCCGCC |
| VHCHR2_4Gln | CCAATACGGATGACGGGGCTCTTCAGAGTGGATTGGGAATTTCTATCAAAGTGGGAACACCCACTACAACTGAAGAGCCCTCTGTTATTGCCGCC |
| VHCHR2_4Asp | CCAATACGGATGACGGGGCTCTTCAGAGTGGATTGGGAATTTCTATGATAGTGGGAACACCCACTACAACTGAAGAGCCCTCTGTTATTGCCGCC |
| VHCHR2_4Glu | CCAATACGGATGACGGGGCTCTTCAGAGTGGATTGGGAATTTCTATGAGAGTGGGAACACCCACTACAACTGAAGAGCCCTCTGTTATTGCCGCC |
| VHCHR2_4Lys | CCAATACGGATGACGGGGCTCTTCAGAGTGGATTGGGAATTTCTATAAAAGTGGGAACACCCACTACAACTGAAGAGCCCTCTGTTATTGCCGCC |
| VHCHR2_4Arg | CCAATACGGATGACGGGGCTCTTCAGAGTGGATTGGGAATTTCTATAGGAGTGGGAACACCCACTACAACTGAAGAGCCCTCTGTTATTGCCGCC |
| VHCHR2_4His | CCAATACGGATGACGGGGCTCTTCAGAGTGGATTGGGAATTTCTATCATAGTGGGAACACCCACTACAACTGAAGAGCCCTCTGTTATTGCCGCC |
| VHCHR2_5Gly | CCAATACGGATGACGGGGCTCTTCATGGATTGGGAATTTCTATTATGGAGGGAACACCCACTACAACCCGTGAAGAGCCCTCTGTTATTGCCGCC |
| VHCHR2_5Ala | CCAATACGGATGACGGGGCTCTTCATGGATTGGGAATTTCTATTATGCGGGGAACACCCACTACAACCCGTGAAGAGCCCTCTGTTATTGCCGCC |
| VHCHR2_5Val | CCAATACGGATGACGGGGCTCTTCATGGATTGGGAATTTCTATTATGTTGGGAACACCCACTACAACCCGTGAAGAGCCCTCTGTTATTGCCGCC |
| VHCHR2_5Leu | CCAATACGGATGACGGGGCTCTTCATGGATTGGGAATTTCTATTATCTTGGGAACACCCACTACAACCCGTGAAGAGCCCTCTGTTATTGCCGCC |
| VHCHR2_5Ile | CCAATACGGATGACGGGGCTCTTCATGGATTGGGAATTTCTATTATATAGGGAACACCCACTACAACCCGTGAAGAGCCCTCTGTTATTGCCGCC |
| VHCHR2_5Pro | CCAATACGGATGACGGGGCTCTTCATGGATTGGGAATTTCTATTATCCCGGGAACACCCACTACAACCCGTGAAGAGCCCTCTGTTATTGCCGCC |
| VHCHR2_5Phe | CCAATACGGATGACGGGGCTCTTCATGGATTGGGAATTTCTATTATTTCGGGAACACCCACTACAACCCGTGAAGAGCCCTCTGTTATTGCCGCC |
| VHCHR2_5Tyr | CCAATACGGATGACGGGGCTCTTCATGGATTGGGAATTTCTATTATTACGGGAACACCCACTACAACCCGTGAAGAGCCCTCTGTTATTGCCGCC |
| VHCHR2_5Trp | CCAATACGGATGACGGGGCTCTTCATGGATTGGGAATTTCTATTATTGGGGGAACACCCACTACAACCCGTGAAGAGCCCTCTGTTATTGCCGCC |
| VHCHR2_5Thr | CCAATACGGATGACGGGGCTCTTCATGGATTGGGAATTTCTATTATACGGGGAACACCCACTACAACCCGTGAAGAGCCCTCTGTTATTGCCGCC |
| VHCHR2_5Cys | CCAATACGGATGACGGGGCTCTTCATGGATTGGGAATTTCTATTATTGCGGGAACACCCACTACAACCCGTGAAGAGCCCTCTGTTATTGCCGCC |
| VHCHR2_5Met | CCAATACGGATGACGGGGCTCTTCATGGATTGGGAATTTCTATTATATGGGGAACACCCACTACAACCCGTGAAGAGCCCTCTGTTATTGCCGCC |
| VHCHR2_5Asn | CCAATACGGATGACGGGGCTCTTCATGGATTGGGAATTTCTATTATAATGGGAACACCCACTACAACCCGTGAAGAGCCCTCTGTTATTGCCGCC |
| VHCHR2_5Gln | CCAATACGGATGACGGGGCTCTTCATGGATTGGGAATTTCTATTATCAGGGGAACACCCACTACAACCCGTGAAGAGCCCTCTGTTATTGCCGCC |
| VHCHR2_5Asp | CCAATACGGATGACGGGGCTCTTCATGGATTGGGAATTTCTATTATGATGGGAACACCCACTACAACCCGTGAAGAGCCCTCTGTTATTGCCGCC |
| VHCHR2_5Glu | CCAATACGGATGACGGGGCTCTTCATGGATTGGGAATTTCTATTATGAGGGGAACACCCACTACAACCCGTGAAGAGCCCTCTGTTATTGCCGCC |
| VHCHR2_5Lys | CCAATACGGATGACGGGGCTCTTCATGGATTGGGAATTTCTATTATAAAGGGAACACCCACTACAACCCGTGAAGAGCCCTCTGTTATTGCCGCC |
| VHCHR2_5Arg | CCAATACGGATGACGGGGCTCTTCATGGATTGGGAATTTCTATTATCGCGGGAACACCCACTACAACCCGTGAAGAGCCCTCTGTTATTGCCGCC |
| VHCHR2_5His | CCAATACGGATGACGGGGCTCTTCATGGATTGGGAATTTCTATTATCACGGGAACACCCACTACAACCCGTGAAGAGCCCTCTGTTATTGCCGCC |
| VHCHR2_6Ala | CCAATACGGATGACGGGGCTCTTCAATTGGGAATTTCTATTATAGTGCGAACACCCACTACAACCCGTCCTGAAGAGCCCTCTGTTATTGCCGCC |
| VHCHR2_6Val | CCAATACGGATGACGGGGCTCTTCAATTGGGAATTTCTATTATAGTGTAAACACCCACTACAACCCGTCCTGAAGAGCCCTCTGTTATTGCCGCC |
| VHCHR2_6Leu | CCAATACGGATGACGGGGCTCTTCAATTGGGAATTTCTATTATAGTCTGAACACCCACTACAACCCGTCCTGAAGAGCCCTCTGTTATTGCCGCC |
| VHCHR2_6Ile | CCAATACGGATGACGGGGCTCTTCAATTGGGAATTTCTATTATAGTATCAACACCCACTACAACCCGTCCTGAAGAGCCCTCTGTTATTGCCGCC |
| VHCHR2_6Pro | CCAATACGGATGACGGGGCTCTTCAATTGGGAATTTCTATTATAGTCCCAACACCCACTACAACCCGTCCTGAAGAGCCCTCTGTTATTGCCGCC |
| VHCHR2_6Phe | CCAATACGGATGACGGGGCTCTTCAATTGGGAATTTCTATTATAGTTTCAACACCCACTACAACCCGTCCTGAAGAGCCCTCTGTTATTGCCGCC |
| VHCHR2_6Tyr | CCAATACGGATGACGGGGCTCTTCAATTGGGAATTTCTATTATAGTTATAACACCCACTACAACCCGTCCTGAAGAGCCCTCTGTTATTGCCGCC |
| VHCHR2_6Trp | CCAATACGGATGACGGGGCTCTTCAATTGGGAATTTCTATTATAGTTGGAACACCCACTACAACCCGTCCTGAAGAGCCCTCTGTTATTGCCGCC |
| VHCHR2_6Ser | CCAATACGGATGACGGGGCTCTTCAATTGGGAATTTCTATTATAGTAGCAACACCCACTACAACCCGTCCTGAAGAGCCCTCTGTTATTGCCGCC |
| VHCHR2_6Thr | CCAATACGGATGACGGGGCTCTTCAATTGGGAATTTCTATTATAGTACCAACACCCACTACAACCCGTCCTGAAGAGCCCTCTGTTATTGCCGCC |
| VHCHR2_6Cys | CCAATACGGATGACGGGGCTCTTCAATTGGGAATTTCTATTATAGTTGTAACACCCACTACAACCCGTCCTGAAGAGCCCTCTGTTATTGCCGCC |
| VHCHR2_6Met | CCAATACGGATGACGGGGCTCTTCAATTGGGAATTTCTATTATAGTATGAACACCCACTACAACCCGTCCTGAAGAGCCCTCTGTTATTGCCGCC |
| VHCHR2_6Asn | CCAATACGGATGACGGGGCTCTTCAATTGGGAATTTCTATTATAGTAATAACACCCACTACAACCCGTCCTGAAGAGCCCTCTGTTATTGCCGCC |
| VHCHR2_6Gln | CCAATACGGATGACGGGGCTCTTCAATTGGGAATTTCTATTATAGTCAAAACACCCACTACAACCCGTCCTGAAGAGCCCTCTGTTATTGCCGCC |
| VHCHR2_6Asp | CCAATACGGATGACGGGGCTCTTCAATTGGGAATTTCTATTATAGTGATAACACCCACTACAACCCGTCCTGAAGAGCCCTCTGTTATTGCCGCC |
| VHCHR2_6Glu | CCAATACGGATGACGGGGCTCTTCAATTGGGAATTTCTATTATAGTGAAAACACCCACTACAACCCGTCCTGAAGAGCCCTCTGTTATTGCCGCC |
| VHCHR2_6Lys | CCAATACGGATGACGGGGCTCTTCAATTGGGAATTTCTATTATAGTAAAAACACCCACTACAACCCGTCCTGAAGAGCCCTCTGTTATTGCCGCC |
| VHCHR2_6Arg | CCAATACGGATGACGGGGCTCTTCAATTGGGAATTTCTATTATAGTCGCAACACCCACTACAACCCGTCCTGAAGAGCCCTCTGTTATTGCCGCC |
| VHCHR2_6His | CCAATACGGATGACGGGGCTCTTCAATTGGGAATTTCTATTATAGTCATAACACCCACTACAACCCGTCCTGAAGAGCCCTCTGTTATTGCCGCC |
| VHCHR2_7Gly | CCAATACGGATGACGGGGCTCTTCAGGGAATTTCTATTATAGTGGGGGTACCCACTACAACCCGTCCCTCTGAAGAGCCCTCTGTTATTGCCGCC |
| VHCHR2_7Ala | CCAATACGGATGACGGGGCTCTTCAGGGAATTTCTATTATAGTGGGGCCACCCACTACAACCCGTCCCTCTGAAGAGCCCTCTGTTATTGCCGCC |
| VHCHR2_7Val | CCAATACGGATGACGGGGCTCTTCAGGGAATTTCTATTATAGTGGGGTGACCCACTACAACCCGTCCCTCTGAAGAGCCCTCTGTTATTGCCGCC |
| VHCHR2_7Leu | CCAATACGGATGACGGGGCTCTTCAGGGAATTTCTATTATAGTGGGTTAACCCACTACAACCCGTCCCTCTGAAGAGCCCTCTGTTATTGCCGCC |
| VHCHR2_7Ile | CCAATACGGATGACGGGGCTCTTCAGGGAATTTCTATTATAGTGGGATAACCCACTACAACCCGTCCCTCTGAAGAGCCCTCTGTTATTGCCGCC |
| VHCHR2_7Pro | CCAATACGGATGACGGGGCTCTTCAGGGAATTTCTATTATAGTGGGCCAACCCACTACAACCCGTCCCTCTGAAGAGCCCTCTGTTATTGCCGCC |
| VHCHR2_7Phe | CCAATACGGATGACGGGGCTCTTCAGGGAATTTCTATTATAGTGGGTTCACCCACTACAACCCGTCCCTCTGAAGAGCCCTCTGTTATTGCCGCC |
| VHCHR2_7Tyr | CCAATACGGATGACGGGGCTCTTCAGGGAATTTCTATTATAGTGGGTATACCCACTACAACCCGTCCCTCTGAAGAGCCCTCTGTTATTGCCGCC |
| VHCHR2_7Trp | CCAATACGGATGACGGGGCTCTTCAGGGAATTTCTATTATAGTGGGTGGACCCACTACAACCCGTCCCTCTGAAGAGCCCTCTGTTATTGCCGCC |
| VHCHR2_7Ser | CCAATACGGATGACGGGGCTCTTCAGGGAATTTCTATTATAGTGGGTCAACCCACTACAACCCGTCCCTCTGAAGAGCCCTCTGTTATTGCCGCC |
| VHCHR2_7Thr | CCAATACGGATGACGGGGCTCTTCAGGGAATTTCTATTATAGTGGGACAACCCACTACAACCCGTCCCTCTGAAGAGCCCTCTGTTATTGCCGCC |
| VHCHR2_7Cys | CCAATACGGATGACGGGGCTCTTCAGGGAATTTCTATTATAGTGGGTGCACCCACTACAACCCGTCCCTCTGAAGAGCCCTCTGTTATTGCCGCC |
| VHCHR2_7Met | CCAATACGGATGACGGGGCTCTTCAGGGAATTTCTATTATAGTGGGATGACCCACTACAACCCGTCCCTCTGAAGAGCCCTCTGTTATTGCCGCC |
| VHCHR2_7Gln | CCAATACGGATGACGGGGCTCTTCAGGGAATTTCTATTATAGTGGGCAAACCCACTACAACCCGTCCCTCTGAAGAGCCCTCTGTTATTGCCGCC |
| VHCHR2_7Asp | CCAATACGGATGACGGGGCTCTTCAGGGAATTTCTATTATAGTGGGGACACCCACTACAACCCGTCCCTCTGAAGAGCCCTCTGTTATTGCCGCC |
| VHCHR2_7Glu | CCAATACGGATGACGGGGCTCTTCAGGGAATTTCTATTATAGTGGGGAAACCCACTACAACCCGTCCCTCTGAAGAGCCCTCTGTTATTGCCGCC |
| VHCHR2_7Lys | CCAATACGGATGACGGGGCTCTTCAGGGAATTTCTATTATAGTGGGAAGACCCACTACAACCCGTCCCTCTGAAGAGCCCTCTGTTATTGCCGCC |
| VHCHR2_7Arg | CCAATACGGATGACGGGGCTCTTCAGGGAATTTCTATTATAGTGGGCGAACCCACTACAACCCGTCCCTCTGAAGAGCCCTCTGTTATTGCCGCC |
| VHCHR2_7His | CCAATACGGATGACGGGGCTCTTCAGGGAATTTCTATTATAGTGGGCATACCCACTACAACCCGTCCCTCTGAAGAGCCCTCTGTTATTGCCGCC |
| VHCHR2_8Gly | CCAATACGGATGACGGGGCTCTTCAAATTTCTATTATAGTGGGAACGGACACTACAACCCGTCCCTCAAGTGAAGAGCCCTCTGTTATTGCCGCC |
| VHCHR2_8Ala | CCAATACGGATGACGGGGCTCTTCAAATTTCTATTATAGTGGGAACGCCCACTACAACCCGTCCCTCAAGTGAAGAGCCCTCTGTTATTGCCGCC |
| VHCHR2_8Val | CCAATACGGATGACGGGGCTCTTCAAATTTCTATTATAGTGGGAACGTCCACTACAACCCGTCCCTCAAGTGAAGAGCCCTCTGTTATTGCCGCC |
| VHCHR2_8Leu | CCAATACGGATGACGGGGCTCTTCAAATTTCTATTATAGTGGGAACTTGCACTACAACCCGTCCCTCAAGTGAAGAGCCCTCTGTTATTGCCGCC |
| VHCHR2_8Ile | CCAATACGGATGACGGGGCTCTTCAAATTTCTATTATAGTGGGAACATTCACTACAACCCGTCCCTCAAGTGAAGAGCCCTCTGTTATTGCCGCC |
| VHCHR2_8Pro | CCAATACGGATGACGGGGCTCTTCAAATTTCTATTATAGTGGGAACCCACACTACAACCCGTCCCTCAAGTGAAGAGCCCTCTGTTATTGCCGCC |
| VHCHR2_8Phe | CCAATACGGATGACGGGGCTCTTCAAATTTCTATTATAGTGGGAACTTCCACTACAACCCGTCCCTCAAGTGAAGAGCCCTCTGTTATTGCCGCC |
| VHCHR2_8Tyr | CCAATACGGATGACGGGGCTCTTCAAATTTCTATTATAGTGGGAACTATCACTACAACCCGTCCCTCAAGTGAAGAGCCCTCTGTTATTGCCGCC |
| VHCHR2_8Trp | CCAATACGGATGACGGGGCTCTTCAAATTTCTATTATAGTGGGAACTGGCACTACAACCCGTCCCTCAAGTGAAGAGCCCTCTGTTATTGCCGCC |
| VHCHR2_8Ser | CCAATACGGATGACGGGGCTCTTCAAATTTCTATTATAGTGGGAACTCTCACTACAACCCGTCCCTCAAGTGAAGAGCCCTCTGTTATTGCCGCC |
| VHCHR2_8Cys | CCAATACGGATGACGGGGCTCTTCAAATTTCTATTATAGTGGGAACTGCCACTACAACCCGTCCCTCAAGTGAAGAGCCCTCTGTTATTGCCGCC |
| VHCHR2_8Met | CCAATACGGATGACGGGGCTCTTCAAATTTCTATTATAGTGGGAACATGCACTACAACCCGTCCCTCAAGTGAAGAGCCCTCTGTTATTGCCGCC |
| VHCHR2_8Asn | CCAATACGGATGACGGGGCTCTTCAAATTTCTATTATAGTGGGAACAACCACTACAACCCGTCCCTCAAGTGAAGAGCCCTCTGTTATTGCCGCC |
| VHCHR2_8Gln | CCAATACGGATGACGGGGCTCTTCAAATTTCTATTATAGTGGGAACCAACACTACAACCCGTCCCTCAAGTGAAGAGCCCTCTGTTATTGCCGCC |
| VHCHR2_8Asp | CCAATACGGATGACGGGGCTCTTCAAATTTCTATTATAGTGGGAACGACCACTACAACCCGTCCCTCAAGTGAAGAGCCCTCTGTTATTGCCGCC |
| VHCHR2_8Glu | CCAATACGGATGACGGGGCTCTTCAAATTTCTATTATAGTGGGAACGAGCACTACAACCCGTCCCTCAAGTGAAGAGCCCTCTGTTATTGCCGCC |
| VHCHR2_8Lys | CCAATACGGATGACGGGGCTCTTCAAATTTCTATTATAGTGGGAACAAACACTACAACCCGTCCCTCAAGTGAAGAGCCCTCTGTTATTGCCGCC |
| VHCHR2_8Arg | CCAATACGGATGACGGGGCTCTTCAAATTTCTATTATAGTGGGAACCGCCACTACAACCCGTCCCTCAAGTGAAGAGCCCTCTGTTATTGCCGCC |
| VHCHR2_8His | CCAATACGGATGACGGGGCTCTTCAAATTTCTATTATAGTGGGAACCATCACTACAACCCGTCCCTCAAGTGAAGAGCCCTCTGTTATTGCCGCC |
| VHCHR2_9Gly | CCAATACGGATGACGGGGCTCTTCATTCTATTATAGTGGGAACACCGGGTACAACCCGTCCCTCAAGAGTTGAAGAGCCCTCTGTTATTGCCGCC |
| VHCHR2_9Ala | CCAATACGGATGACGGGGCTCTTCATTCTATTATAGTGGGAACACCGCTTACAACCCGTCCCTCAAGAGTTGAAGAGCCCTCTGTTATTGCCGCC |
| VHCHR2_9Val | CCAATACGGATGACGGGGCTCTTCATTCTATTATAGTGGGAACACCGTTTACAACCCGTCCCTCAAGAGTTGAAGAGCCCTCTGTTATTGCCGCC |
| VHCHR2_9Leu | CCAATACGGATGACGGGGCTCTTCATTCTATTATAGTGGGAACACCCTATACAACCCGTCCCTCAAGAGTTGAAGAGCCCTCTGTTATTGCCGCC |
| VHCHR2_9Ile | CCAATACGGATGACGGGGCTCTTCATTCTATTATAGTGGGAACACCATATACAACCCGTCCCTCAAGAGTTGAAGAGCCCTCTGTTATTGCCGCC |
| VHCHR2_9Pro | CCAATACGGATGACGGGGCTCTTCATTCTATTATAGTGGGAACACCCCTTACAACCCGTCCCTCAAGAGTTGAAGAGCCCTCTGTTATTGCCGCC |
| VHCHR2_9Phe | CCAATACGGATGACGGGGCTCTTCATTCTATTATAGTGGGAACACCTTCTACAACCCGTCCCTCAAGAGTTGAAGAGCCCTCTGTTATTGCCGCC |
| VHCHR2_9Tyr | CCAATACGGATGACGGGGCTCTTCATTCTATTATAGTGGGAACACCTACTACAACCCGTCCCTCAAGAGTTGAAGAGCCCTCTGTTATTGCCGCC |
| VHCHR2_9Trp | CCAATACGGATGACGGGGCTCTTCATTCTATTATAGTGGGAACACCTGGTACAACCCGTCCCTCAAGAGTTGAAGAGCCCTCTGTTATTGCCGCC |
| VHCHR2_9Ser | CCAATACGGATGACGGGGCTCTTCATTCTATTATAGTGGGAACACCTCTTACAACCCGTCCCTCAAGAGTTGAAGAGCCCTCTGTTATTGCCGCC |
| VHCHR2_9Thr | CCAATACGGATGACGGGGCTCTTCATTCTATTATAGTGGGAACACCACTTACAACCCGTCCCTCAAGAGTTGAAGAGCCCTCTGTTATTGCCGCC |
| VHCHR2_9Cys | CCAATACGGATGACGGGGCTCTTCATTCTATTATAGTGGGAACACCTGTTACAACCCGTCCCTCAAGAGTTGAAGAGCCCTCTGTTATTGCCGCC |
| VHCHR2_9Met | CCAATACGGATGACGGGGCTCTTCATTCTATTATAGTGGGAACACCATGTACAACCCGTCCCTCAAGAGTTGAAGAGCCCTCTGTTATTGCCGCC |
| VHCHR2_9Asn | CCAATACGGATGACGGGGCTCTTCATTCTATTATAGTGGGAACACCAATTACAACCCGTCCCTCAAGAGTTGAAGAGCCCTCTGTTATTGCCGCC |
| VHCHR2_9Gln | CCAATACGGATGACGGGGCTCTTCATTCTATTATAGTGGGAACACCCAATACAACCCGTCCCTCAAGAGTTGAAGAGCCCTCTGTTATTGCCGCC |
| VHCHR2_9Asp | CCAATACGGATGACGGGGCTCTTCATTCTATTATAGTGGGAACACCGACTACAACCCGTCCCTCAAGAGTTGAAGAGCCCTCTGTTATTGCCGCC |
| VHCHR2_9Glu | CCAATACGGATGACGGGGCTCTTCATTCTATTATAGTGGGAACACCGAATACAACCCGTCCCTCAAGAGTTGAAGAGCCCTCTGTTATTGCCGCC |
| VHCHR2_9Lys | CCAATACGGATGACGGGGCTCTTCATTCTATTATAGTGGGAACACCAAGTACAACCCGTCCCTCAAGAGTTGAAGAGCCCTCTGTTATTGCCGCC |
| VHCHR2_9Arg | CCAATACGGATGACGGGGCTCTTCATTCTATTATAGTGGGAACACCCGTTACAACCCGTCCCTCAAGAGTTGAAGAGCCCTCTGTTATTGCCGCC |
| VHCHR2_10Gly | CCAATACGGATGACGGGGCTCTTCATATTATAGTGGGAACACCCACGGGAACCCGTCCCTCAAGAGTCGATGAAGAGCCCTCTGTTATTGCCGCC |
| VHCHR2_10Ala | CCAATACGGATGACGGGGCTCTTCATATTATAGTGGGAACACCCACGCAAACCCGTCCCTCAAGAGTCGATGAAGAGCCCTCTGTTATTGCCGCC |
| VHCHR2_10Val | CCAATACGGATGACGGGGCTCTTCATATTATAGTGGGAACACCCACGTTAACCCGTCCCTCAAGAGTCGATGAAGAGCCCTCTGTTATTGCCGCC |
| VHCHR2_10Leu | CCAATACGGATGACGGGGCTCTTCATATTATAGTGGGAACACCCACCTTAACCCGTCCCTCAAGAGTCGATGAAGAGCCCTCTGTTATTGCCGCC |
| VHCHR2_10Ile | CCAATACGGATGACGGGGCTCTTCATATTATAGTGGGAACACCCACATTAACCCGTCCCTCAAGAGTCGATGAAGAGCCCTCTGTTATTGCCGCC |
| VHCHR2_10Pro | CCAATACGGATGACGGGGCTCTTCATATTATAGTGGGAACACCCACCCCAACCCGTCCCTCAAGAGTCGATGAAGAGCCCTCTGTTATTGCCGCC |
| VHCHR2_10Phe | CCAATACGGATGACGGGGCTCTTCATATTATAGTGGGAACACCCACTTCAACCCGTCCCTCAAGAGTCGATGAAGAGCCCTCTGTTATTGCCGCC |
| VHCHR2_10Trp | CCAATACGGATGACGGGGCTCTTCATATTATAGTGGGAACACCCACTGGAACCCGTCCCTCAAGAGTCGATGAAGAGCCCTCTGTTATTGCCGCC |
| VHCHR2_10Ser | CCAATACGGATGACGGGGCTCTTCATATTATAGTGGGAACACCCACAGCAACCCGTCCCTCAAGAGTCGATGAAGAGCCCTCTGTTATTGCCGCC |
| VHCHR2_10Thr | CCAATACGGATGACGGGGCTCTTCATATTATAGTGGGAACACCCACACTAACCCGTCCCTCAAGAGTCGATGAAGAGCCCTCTGTTATTGCCGCC |
| VHCHR2_10Cys | CCAATACGGATGACGGGGCTCTTCATATTATAGTGGGAACACCCACTGTAACCCGTCCCTCAAGAGTCGATGAAGAGCCCTCTGTTATTGCCGCC |
| VHCHR2_10Met | CCAATACGGATGACGGGGCTCTTCATATTATAGTGGGAACACCCACATGAACCCGTCCCTCAAGAGTCGATGAAGAGCCCTCTGTTATTGCCGCC |
| VHCHR2_10Asn | CCAATACGGATGACGGGGCTCTTCATATTATAGTGGGAACACCCACAATAACCCGTCCCTCAAGAGTCGATGAAGAGCCCTCTGTTATTGCCGCC |
| VHCHR2_10Gln | CCAATACGGATGACGGGGCTCTTCATATTATAGTGGGAACACCCACCAAAACCCGTCCCTCAAGAGTCGATGAAGAGCCCTCTGTTATTGCCGCC |
| VHCHR2_10Asp | CCAATACGGATGACGGGGCTCTTCATATTATAGTGGGAACACCCACGATAACCCGTCCCTCAAGAGTCGATGAAGAGCCCTCTGTTATTGCCGCC |
| VHCHR2_10Glu | CCAATACGGATGACGGGGCTCTTCATATTATAGTGGGAACACCCACGAAAACCCGTCCCTCAAGAGTCGATGAAGAGCCCTCTGTTATTGCCGCC |
| VHCHR2_10Lys | CCAATACGGATGACGGGGCTCTTCATATTATAGTGGGAACACCCACAAAAACCCGTCCCTCAAGAGTCGATGAAGAGCCCTCTGTTATTGCCGCC |
| VHCHR2_10Arg | CCAATACGGATGACGGGGCTCTTCATATTATAGTGGGAACACCCACCGGAACCCGTCCCTCAAGAGTCGATGAAGAGCCCTCTGTTATTGCCGCC |
| VHCHR2_10His | CCAATACGGATGACGGGGCTCTTCATATTATAGTGGGAACACCCACCATAACCCGTCCCTCAAGAGTCGATGAAGAGCCCTCTGTTATTGCCGCC |
| VHCHR2_11Gly | CCAATACGGATGACGGGGCTCTTCATATAGTGGGAACACCCACTACGGTCCGTCCCTCAAGAGTCGAGTCTGAAGAGCCCTCTGTTATTGCCGCC |
| VHCHR2_11Ala | CCAATACGGATGACGGGGCTCTTCATATAGTGGGAACACCCACTACGCGCCGTCCCTCAAGAGTCGAGTCTGAAGAGCCCTCTGTTATTGCCGCC |
| VHCHR2_11Val | CCAATACGGATGACGGGGCTCTTCATATAGTGGGAACACCCACTACGTACCGTCCCTCAAGAGTCGAGTCTGAAGAGCCCTCTGTTATTGCCGCC |
| VHCHR2_11Leu | CCAATACGGATGACGGGGCTCTTCATATAGTGGGAACACCCACTACCTTCCGTCCCTCAAGAGTCGAGTCTGAAGAGCCCTCTGTTATTGCCGCC |
| VHCHR2_11Ile | CCAATACGGATGACGGGGCTCTTCATATAGTGGGAACACCCACTACATTCCGTCCCTCAAGAGTCGAGTCTGAAGAGCCCTCTGTTATTGCCGCC |
| VHCHR2_11Pro | CCAATACGGATGACGGGGCTCTTCATATAGTGGGAACACCCACTACCCTCCGTCCCTCAAGAGTCGAGTCTGAAGAGCCCTCTGTTATTGCCGCC |
| VHCHR2_11Phe | CCAATACGGATGACGGGGCTCTTCATATAGTGGGAACACCCACTACTTTCCGTCCCTCAAGAGTCGAGTCTGAAGAGCCCTCTGTTATTGCCGCC |
| VHCHR2_11Tyr | CCAATACGGATGACGGGGCTCTTCATATAGTGGGAACACCCACTACTATCCGTCCCTCAAGAGTCGAGTCTGAAGAGCCCTCTGTTATTGCCGCC |
| VHCHR2_11Trp | CCAATACGGATGACGGGGCTCTTCATATAGTGGGAACACCCACTACTGGCCGTCCCTCAAGAGTCGAGTCTGAAGAGCCCTCTGTTATTGCCGCC |
| VHCHR2_11Ser | CCAATACGGATGACGGGGCTCTTCATATAGTGGGAACACCCACTACAGCCCGTCCCTCAAGAGTCGAGTCTGAAGAGCCCTCTGTTATTGCCGCC |
| VHCHR2_11Thr | CCAATACGGATGACGGGGCTCTTCATATAGTGGGAACACCCACTACACGCCGTCCCTCAAGAGTCGAGTCTGAAGAGCCCTCTGTTATTGCCGCC |
| VHCHR2_11Cys | CCAATACGGATGACGGGGCTCTTCATATAGTGGGAACACCCACTACTGCCCGTCCCTCAAGAGTCGAGTCTGAAGAGCCCTCTGTTATTGCCGCC |
| VHCHR2_11Met | CCAATACGGATGACGGGGCTCTTCATATAGTGGGAACACCCACTACATGCCGTCCCTCAAGAGTCGAGTCTGAAGAGCCCTCTGTTATTGCCGCC |
| VHCHR2_11Gln | CCAATACGGATGACGGGGCTCTTCATATAGTGGGAACACCCACTACCAGCCGTCCCTCAAGAGTCGAGTCTGAAGAGCCCTCTGTTATTGCCGCC |
| VHCHR2_11Asp | CCAATACGGATGACGGGGCTCTTCATATAGTGGGAACACCCACTACGACCCGTCCCTCAAGAGTCGAGTCTGAAGAGCCCTCTGTTATTGCCGCC |
| VHCHR2_11Glu | CCAATACGGATGACGGGGCTCTTCATATAGTGGGAACACCCACTACGAGCCGTCCCTCAAGAGTCGAGTCTGAAGAGCCCTCTGTTATTGCCGCC |
| VHCHR2_11Lys | CCAATACGGATGACGGGGCTCTTCATATAGTGGGAACACCCACTACAAGCCGTCCCTCAAGAGTCGAGTCTGAAGAGCCCTCTGTTATTGCCGCC |
| VHCHR2_11Arg | CCAATACGGATGACGGGGCTCTTCATATAGTGGGAACACCCACTACCGTCCGTCCCTCAAGAGTCGAGTCTGAAGAGCCCTCTGTTATTGCCGCC |
| VHCHR2_11His | CCAATACGGATGACGGGGCTCTTCATATAGTGGGAACACCCACTACCATCCGTCCCTCAAGAGTCGAGTCTGAAGAGCCCTCTGTTATTGCCGCC |
| VHCHR2_12Gly | CCAATACGGATGACGGGGCTCTTCAAGTGGGAACACCCACTACAACGGATCCCTCAAGAGTCGAGTCACCTGAAGAGCCCTCTGTTATTGCCGCC |
| VHCHR2_12Ala | CCAATACGGATGACGGGGCTCTTCAAGTGGGAACACCCACTACAACGCATCCCTCAAGAGTCGAGTCACCTGAAGAGCCCTCTGTTATTGCCGCC |
| VHCHR2_12Val | CCAATACGGATGACGGGGCTCTTCAAGTGGGAACACCCACTACAACGTCTCCCTCAAGAGTCGAGTCACCTGAAGAGCCCTCTGTTATTGCCGCC |
| VHCHR2_12Leu | CCAATACGGATGACGGGGCTCTTCAAGTGGGAACACCCACTACAACCTGTCCCTCAAGAGTCGAGTCACCTGAAGAGCCCTCTGTTATTGCCGCC |
| VHCHR2_12Ile | CCAATACGGATGACGGGGCTCTTCAAGTGGGAACACCCACTACAACATCTCCCTCAAGAGTCGAGTCACCTGAAGAGCCCTCTGTTATTGCCGCC |
| VHCHR2_12Phe | CCAATACGGATGACGGGGCTCTTCAAGTGGGAACACCCACTACAACTTTTCCCTCAAGAGTCGAGTCACCTGAAGAGCCCTCTGTTATTGCCGCC |
| VHCHR2_12Tyr | CCAATACGGATGACGGGGCTCTTCAAGTGGGAACACCCACTACAACTACTCCCTCAAGAGTCGAGTCACCTGAAGAGCCCTCTGTTATTGCCGCC |
| VHCHR2_12Trp | CCAATACGGATGACGGGGCTCTTCAAGTGGGAACACCCACTACAACTGGTCCCTCAAGAGTCGAGTCACCTGAAGAGCCCTCTGTTATTGCCGCC |
| VHCHR2_12Ser | CCAATACGGATGACGGGGCTCTTCAAGTGGGAACACCCACTACAACAGTTCCCTCAAGAGTCGAGTCACCTGAAGAGCCCTCTGTTATTGCCGCC |
| VHCHR2_12Thr | CCAATACGGATGACGGGGCTCTTCAAGTGGGAACACCCACTACAACACCTCCCTCAAGAGTCGAGTCACCTGAAGAGCCCTCTGTTATTGCCGCC |
| VHCHR2_12Cys | CCAATACGGATGACGGGGCTCTTCAAGTGGGAACACCCACTACAACTGCTCCCTCAAGAGTCGAGTCACCTGAAGAGCCCTCTGTTATTGCCGCC |
| VHCHR2_12Met | CCAATACGGATGACGGGGCTCTTCAAGTGGGAACACCCACTACAACATGTCCCTCAAGAGTCGAGTCACCTGAAGAGCCCTCTGTTATTGCCGCC |
| VHCHR2_12Asn | CCAATACGGATGACGGGGCTCTTCAAGTGGGAACACCCACTACAACAACTCCCTCAAGAGTCGAGTCACCTGAAGAGCCCTCTGTTATTGCCGCC |
| VHCHR2_12Gln | CCAATACGGATGACGGGGCTCTTCAAGTGGGAACACCCACTACAACCAGTCCCTCAAGAGTCGAGTCACCTGAAGAGCCCTCTGTTATTGCCGCC |
| VHCHR2_12Asp | CCAATACGGATGACGGGGCTCTTCAAGTGGGAACACCCACTACAACGATTCCCTCAAGAGTCGAGTCACCTGAAGAGCCCTCTGTTATTGCCGCC |
| VHCHR2_12Glu | CCAATACGGATGACGGGGCTCTTCAAGTGGGAACACCCACTACAACGAATCCCTCAAGAGTCGAGTCACCTGAAGAGCCCTCTGTTATTGCCGCC |
| VHCHR2_12Lys | CCAATACGGATGACGGGGCTCTTCAAGTGGGAACACCCACTACAACAAATCCCTCAAGAGTCGAGTCACCTGAAGAGCCCTCTGTTATTGCCGCC |
| VHCHR2_12Arg | CCAATACGGATGACGGGGCTCTTCAAGTGGGAACACCCACTACAACCGATCCCTCAAGAGTCGAGTCACCTGAAGAGCCCTCTGTTATTGCCGCC |
| VHCHR2_12His | CCAATACGGATGACGGGGCTCTTCAAGTGGGAACACCCACTACAACCATTCCCTCAAGAGTCGAGTCACCTGAAGAGCCCTCTGTTATTGCCGCC |
| VHCHR2_13Gly | CCAATACGGATGACGGGGCTCTTCAGGGAACACCCACTACAACCCGGGCCTCAAGAGTCGAGTCACCATATGAAGAGCCCTCTGTTATTGCCGCC |
| VHCHR2_13Ala | CCAATACGGATGACGGGGCTCTTCAGGGAACACCCACTACAACCCGGCACTCAAGAGTCGAGTCACCATATGAAGAGCCCTCTGTTATTGCCGCC |
| VHCHR2_13Val | CCAATACGGATGACGGGGCTCTTCAGGGAACACCCACTACAACCCGGTGCTCAAGAGTCGAGTCACCATATGAAGAGCCCTCTGTTATTGCCGCC |
| VHCHR2_13Leu | CCAATACGGATGACGGGGCTCTTCAGGGAACACCCACTACAACCCGCTCCTCAAGAGTCGAGTCACCATATGAAGAGCCCTCTGTTATTGCCGCC |
| VHCHR2_13Ile | CCAATACGGATGACGGGGCTCTTCAGGGAACACCCACTACAACCCGATTCTCAAGAGTCGAGTCACCATATGAAGAGCCCTCTGTTATTGCCGCC |
| VHCHR2_13Pro | CCAATACGGATGACGGGGCTCTTCAGGGAACACCCACTACAACCCGCCTCTCAAGAGTCGAGTCACCATATGAAGAGCCCTCTGTTATTGCCGCC |
| VHCHR2_13Phe | CCAATACGGATGACGGGGCTCTTCAGGGAACACCCACTACAACCCGTTTCTCAAGAGTCGAGTCACCATATGAAGAGCCCTCTGTTATTGCCGCC |
| VHCHR2_13Tyr | CCAATACGGATGACGGGGCTCTTCAGGGAACACCCACTACAACCCGTATCTCAAGAGTCGAGTCACCATATGAAGAGCCCTCTGTTATTGCCGCC |
| VHCHR2_13Trp | CCAATACGGATGACGGGGCTCTTCAGGGAACACCCACTACAACCCGTGGCTCAAGAGTCGAGTCACCATATGAAGAGCCCTCTGTTATTGCCGCC |
| VHCHR2_13Thr | CCAATACGGATGACGGGGCTCTTCAGGGAACACCCACTACAACCCGACTCTCAAGAGTCGAGTCACCATATGAAGAGCCCTCTGTTATTGCCGCC |
| VHCHR2_13Cys | CCAATACGGATGACGGGGCTCTTCAGGGAACACCCACTACAACCCGTGCCTCAAGAGTCGAGTCACCATATGAAGAGCCCTCTGTTATTGCCGCC |
| VHCHR2_13Met | CCAATACGGATGACGGGGCTCTTCAGGGAACACCCACTACAACCCGATGCTCAAGAGTCGAGTCACCATATGAAGAGCCCTCTGTTATTGCCGCC |
| VHCHR2_13Asn | CCAATACGGATGACGGGGCTCTTCAGGGAACACCCACTACAACCCGAATCTCAAGAGTCGAGTCACCATATGAAGAGCCCTCTGTTATTGCCGCC |
| VHCHR2_13Gln | CCAATACGGATGACGGGGCTCTTCAGGGAACACCCACTACAACCCGCAGCTCAAGAGTCGAGTCACCATATGAAGAGCCCTCTGTTATTGCCGCC |
| VHCHR2_13Asp | CCAATACGGATGACGGGGCTCTTCAGGGAACACCCACTACAACCCGGATCTCAAGAGTCGAGTCACCATATGAAGAGCCCTCTGTTATTGCCGCC |
| VHCHR2_13Glu | CCAATACGGATGACGGGGCTCTTCAGGGAACACCCACTACAACCCGGAGCTCAAGAGTCGAGTCACCATATGAAGAGCCCTCTGTTATTGCCGCC |
| VHCHR2_13Lys | CCAATACGGATGACGGGGCTCTTCAGGGAACACCCACTACAACCCGAAACTCAAGAGTCGAGTCACCATATGAAGAGCCCTCTGTTATTGCCGCC |
| VHCHR2_13Arg | CCAATACGGATGACGGGGCTCTTCAGGGAACACCCACTACAACCCGCGACTCAAGAGTCGAGTCACCATATGAAGAGCCCTCTGTTATTGCCGCC |
| VHCHR2_13His | CCAATACGGATGACGGGGCTCTTCAGGGAACACCCACTACAACCCGCATCTCAAGAGTCGAGTCACCATATGAAGAGCCCTCTGTTATTGCCGCC |
| VHCHR2_14Gly | CCAATACGGATGACGGGGCTCTTCAAACACCCACTACAACCCGTCCGGGAAGAGTCGAGTCACCATATCCTGAAGAGCCCTCTGTTATTGCCGCC |
| VHCHR2_14Ala | CCAATACGGATGACGGGGCTCTTCAAACACCCACTACAACCCGTCCGCCAAGAGTCGAGTCACCATATCCTGAAGAGCCCTCTGTTATTGCCGCC |
| VHCHR2_14Val | CCAATACGGATGACGGGGCTCTTCAAACACCCACTACAACCCGTCCGTAAAGAGTCGAGTCACCATATCCTGAAGAGCCCTCTGTTATTGCCGCC |
| VHCHR2_14Ile | CCAATACGGATGACGGGGCTCTTCAAACACCCACTACAACCCGTCCATCAAGAGTCGAGTCACCATATCCTGAAGAGCCCTCTGTTATTGCCGCC |
| VHCHR2_14Pro | CCAATACGGATGACGGGGCTCTTCAAACACCCACTACAACCCGTCCCCAAAGAGTCGAGTCACCATATCCTGAAGAGCCCTCTGTTATTGCCGCC |
| VHCHR2_14Phe | CCAATACGGATGACGGGGCTCTTCAAACACCCACTACAACCCGTCCTTTAAGAGTCGAGTCACCATATCCTGAAGAGCCCTCTGTTATTGCCGCC |
| VHCHR2_14Tyr | CCAATACGGATGACGGGGCTCTTCAAACACCCACTACAACCCGTCCTATAAGAGTCGAGTCACCATATCCTGAAGAGCCCTCTGTTATTGCCGCC |
| VHCHR2_14Trp | CCAATACGGATGACGGGGCTCTTCAAACACCCACTACAACCCGTCCTGGAAGAGTCGAGTCACCATATCCTGAAGAGCCCTCTGTTATTGCCGCC |
| VHCHR2_14Ser | CCAATACGGATGACGGGGCTCTTCAAACACCCACTACAACCCGTCCTCGAAGAGTCGAGTCACCATATCCTGAAGAGCCCTCTGTTATTGCCGCC |
| VHCHR2_14Thr | CCAATACGGATGACGGGGCTCTTCAAACACCCACTACAACCCGTCCACTAAGAGTCGAGTCACCATATCCTGAAGAGCCCTCTGTTATTGCCGCC |
| VHCHR2_14Cys | CCAATACGGATGACGGGGCTCTTCAAACACCCACTACAACCCGTCCTGTAAGAGTCGAGTCACCATATCCTGAAGAGCCCTCTGTTATTGCCGCC |
| VHCHR2_14Met | CCAATACGGATGACGGGGCTCTTCAAACACCCACTACAACCCGTCCATGAAGAGTCGAGTCACCATATCCTGAAGAGCCCTCTGTTATTGCCGCC |
| VHCHR2_14Asn | CCAATACGGATGACGGGGCTCTTCAAACACCCACTACAACCCGTCCAATAAGAGTCGAGTCACCATATCCTGAAGAGCCCTCTGTTATTGCCGCC |
| VHCHR2_14Gln | CCAATACGGATGACGGGGCTCTTCAAACACCCACTACAACCCGTCCCAAAAGAGTCGAGTCACCATATCCTGAAGAGCCCTCTGTTATTGCCGCC |
| VHCHR2_14Asp | CCAATACGGATGACGGGGCTCTTCAAACACCCACTACAACCCGTCCGATAAGAGTCGAGTCACCATATCCTGAAGAGCCCTCTGTTATTGCCGCC |
| VHCHR2_14Glu | CCAATACGGATGACGGGGCTCTTCAAACACCCACTACAACCCGTCCGAAAAGAGTCGAGTCACCATATCCTGAAGAGCCCTCTGTTATTGCCGCC |
| VHCHR2_14Lys | CCAATACGGATGACGGGGCTCTTCAAACACCCACTACAACCCGTCCAAGAAGAGTCGAGTCACCATATCCTGAAGAGCCCTCTGTTATTGCCGCC |
| VHCHR2_14Arg | CCAATACGGATGACGGGGCTCTTCAAACACCCACTACAACCCGTCCCGAAAGAGTCGAGTCACCATATCCTGAAGAGCCCTCTGTTATTGCCGCC |
| VHCHR2_14His | CCAATACGGATGACGGGGCTCTTCAAACACCCACTACAACCCGTCCCATAAGAGTCGAGTCACCATATCCTGAAGAGCCCTCTGTTATTGCCGCC |
| VHCHR2_15Gly | CCAATACGGATGACGGGGCTCTTCAACCCACTACAACCCGTCCCTCGGCAGTCGAGTCACCATATCCGTATGAAGAGCCCTCTGTTATTGCCGCC |
| VHCHR2_15Ala | CCAATACGGATGACGGGGCTCTTCAACCCACTACAACCCGTCCCTCGCCAGTCGAGTCACCATATCCGTATGAAGAGCCCTCTGTTATTGCCGCC |
| VHCHR2_15Val | CCAATACGGATGACGGGGCTCTTCAACCCACTACAACCCGTCCCTCGTAAGTCGAGTCACCATATCCGTATGAAGAGCCCTCTGTTATTGCCGCC |
| VHCHR2_15Leu | CCAATACGGATGACGGGGCTCTTCAACCCACTACAACCCGTCCCTCTTAAGTCGAGTCACCATATCCGTATGAAGAGCCCTCTGTTATTGCCGCC |
| VHCHR2_15Ile | CCAATACGGATGACGGGGCTCTTCAACCCACTACAACCCGTCCCTCATCAGTCGAGTCACCATATCCGTATGAAGAGCCCTCTGTTATTGCCGCC |
| VHCHR2_15Pro | CCAATACGGATGACGGGGCTCTTCAACCCACTACAACCCGTCCCTCCCAAGTCGAGTCACCATATCCGTATGAAGAGCCCTCTGTTATTGCCGCC |
| VHCHR2_15Phe | CCAATACGGATGACGGGGCTCTTCAACCCACTACAACCCGTCCCTCTTTAGTCGAGTCACCATATCCGTATGAAGAGCCCTCTGTTATTGCCGCC |
| VHCHR2_15Tyr | CCAATACGGATGACGGGGCTCTTCAACCCACTACAACCCGTCCCTCTACAGTCGAGTCACCATATCCGTATGAAGAGCCCTCTGTTATTGCCGCC |
| VHCHR2_15Trp | CCAATACGGATGACGGGGCTCTTCAACCCACTACAACCCGTCCCTCTGGAGTCGAGTCACCATATCCGTATGAAGAGCCCTCTGTTATTGCCGCC |
| VHCHR2_15Ser | CCAATACGGATGACGGGGCTCTTCAACCCACTACAACCCGTCCCTCAGTAGTCGAGTCACCATATCCGTATGAAGAGCCCTCTGTTATTGCCGCC |
| VHCHR2_15Thr | CCAATACGGATGACGGGGCTCTTCAACCCACTACAACCCGTCCCTCACGAGTCGAGTCACCATATCCGTATGAAGAGCCCTCTGTTATTGCCGCC |
| VHCHR2_15Cys | CCAATACGGATGACGGGGCTCTTCAACCCACTACAACCCGTCCCTCTGCAGTCGAGTCACCATATCCGTATGAAGAGCCCTCTGTTATTGCCGCC |
| VHCHR2_15Met | CCAATACGGATGACGGGGCTCTTCAACCCACTACAACCCGTCCCTCATGAGTCGAGTCACCATATCCGTATGAAGAGCCCTCTGTTATTGCCGCC |
| VHCHR2_15Asn | CCAATACGGATGACGGGGCTCTTCAACCCACTACAACCCGTCCCTCAACAGTCGAGTCACCATATCCGTATGAAGAGCCCTCTGTTATTGCCGCC |
| VHCHR2_15Gln | CCAATACGGATGACGGGGCTCTTCAACCCACTACAACCCGTCCCTCCAAAGTCGAGTCACCATATCCGTATGAAGAGCCCTCTGTTATTGCCGCC |
| VHCHR2_15Asp | CCAATACGGATGACGGGGCTCTTCAACCCACTACAACCCGTCCCTCGATAGTCGAGTCACCATATCCGTATGAAGAGCCCTCTGTTATTGCCGCC |
| VHCHR2_15Glu | CCAATACGGATGACGGGGCTCTTCAACCCACTACAACCCGTCCCTCGAGAGTCGAGTCACCATATCCGTATGAAGAGCCCTCTGTTATTGCCGCC |
| VHCHR2_15Arg | CCAATACGGATGACGGGGCTCTTCAACCCACTACAACCCGTCCCTCCGTAGTCGAGTCACCATATCCGTATGAAGAGCCCTCTGTTATTGCCGCC |
| VHCHR2_15His | CCAATACGGATGACGGGGCTCTTCAACCCACTACAACCCGTCCCTCCACAGTCGAGTCACCATATCCGTATGAAGAGCCCTCTGTTATTGCCGCC |
| VHCHR2_16Gly | CCAATACGGATGACGGGGCTCTTCACACTACAACCCGTCCCTCAAGGGTCGAGTCACCATATCCGTAGGCTGAAGAGCCCTCTGTTATTGCCGCC |
| VHCHR2_16Ala | CCAATACGGATGACGGGGCTCTTCACACTACAACCCGTCCCTCAAGGCCCGAGTCACCATATCCGTAGGCTGAAGAGCCCTCTGTTATTGCCGCC |
| VHCHR2_16Val | CCAATACGGATGACGGGGCTCTTCACACTACAACCCGTCCCTCAAGGTACGAGTCACCATATCCGTAGGCTGAAGAGCCCTCTGTTATTGCCGCC |
| VHCHR2_16Leu | CCAATACGGATGACGGGGCTCTTCACACTACAACCCGTCCCTCAAGTTGCGAGTCACCATATCCGTAGGCTGAAGAGCCCTCTGTTATTGCCGCC |
| VHCHR2_16Ile | CCAATACGGATGACGGGGCTCTTCACACTACAACCCGTCCCTCAAGATTCGAGTCACCATATCCGTAGGCTGAAGAGCCCTCTGTTATTGCCGCC |
| VHCHR2_16Pro | CCAATACGGATGACGGGGCTCTTCACACTACAACCCGTCCCTCAAGCCGCGAGTCACCATATCCGTAGGCTGAAGAGCCCTCTGTTATTGCCGCC |
| VHCHR2_16Phe | CCAATACGGATGACGGGGCTCTTCACACTACAACCCGTCCCTCAAGTTCCGAGTCACCATATCCGTAGGCTGAAGAGCCCTCTGTTATTGCCGCC |
| VHCHR2_16Tyr | CCAATACGGATGACGGGGCTCTTCACACTACAACCCGTCCCTCAAGTATCGAGTCACCATATCCGTAGGCTGAAGAGCCCTCTGTTATTGCCGCC |
| VHCHR2_16Trp | CCAATACGGATGACGGGGCTCTTCACACTACAACCCGTCCCTCAAGTGGCGAGTCACCATATCCGTAGGCTGAAGAGCCCTCTGTTATTGCCGCC |
| VHCHR2_16Thr | CCAATACGGATGACGGGGCTCTTCACACTACAACCCGTCCCTCAAGACCCGAGTCACCATATCCGTAGGCTGAAGAGCCCTCTGTTATTGCCGCC |
| VHCHR2_16Cys | CCAATACGGATGACGGGGCTCTTCACACTACAACCCGTCCCTCAAGTGTCGAGTCACCATATCCGTAGGCTGAAGAGCCCTCTGTTATTGCCGCC |
| VHCHR2_16Met | CCAATACGGATGACGGGGCTCTTCACACTACAACCCGTCCCTCAAGATGCGAGTCACCATATCCGTAGGCTGAAGAGCCCTCTGTTATTGCCGCC |
| VHCHR2_16Asn | CCAATACGGATGACGGGGCTCTTCACACTACAACCCGTCCCTCAAGAACCGAGTCACCATATCCGTAGGCTGAAGAGCCCTCTGTTATTGCCGCC |
| VHCHR2_16Gln | CCAATACGGATGACGGGGCTCTTCACACTACAACCCGTCCCTCAAGCAACGAGTCACCATATCCGTAGGCTGAAGAGCCCTCTGTTATTGCCGCC |
| VHCHR2_16Asp | CCAATACGGATGACGGGGCTCTTCACACTACAACCCGTCCCTCAAGGACCGAGTCACCATATCCGTAGGCTGAAGAGCCCTCTGTTATTGCCGCC |
| VHCHR2_16Glu | CCAATACGGATGACGGGGCTCTTCACACTACAACCCGTCCCTCAAGGAGCGAGTCACCATATCCGTAGGCTGAAGAGCCCTCTGTTATTGCCGCC |
| VHCHR2_16Lys | CCAATACGGATGACGGGGCTCTTCACACTACAACCCGTCCCTCAAGAAGCGAGTCACCATATCCGTAGGCTGAAGAGCCCTCTGTTATTGCCGCC |
| VHCHR2_16Arg | CCAATACGGATGACGGGGCTCTTCACACTACAACCCGTCCCTCAAGCGCCGAGTCACCATATCCGTAGGCTGAAGAGCCCTCTGTTATTGCCGCC |
| VHCHR2_16His | CCAATACGGATGACGGGGCTCTTCACACTACAACCCGTCCCTCAAGCATCGAGTCACCATATCCGTAGGCTGAAGAGCCCTCTGTTATTGCCGCC |
| VHCHR3_1Gly | CCAATACGGATGACGGGGCTCTTCAGACACGGCTGTATATTACTGTGGTAGACAGTCGACCATAGGGGGCTGAAGAGCCCTCTGTTATTGCCGCC |
| VHCHR3_1Val | CCAATACGGATGACGGGGCTCTTCAGACACGGCTGTATATTACTGTGTGAGACAGTCGACCATAGGGGGCTGAAGAGCCCTCTGTTATTGCCGCC |
| VHCHR3_1Leu | CCAATACGGATGACGGGGCTCTTCAGACACGGCTGTATATTACTGTCTCAGACAGTCGACCATAGGGGGCTGAAGAGCCCTCTGTTATTGCCGCC |
| VHCHR3_1Ile | CCAATACGGATGACGGGGCTCTTCAGACACGGCTGTATATTACTGTATAAGACAGTCGACCATAGGGGGCTGAAGAGCCCTCTGTTATTGCCGCC |
| VHCHR3_1Pro | CCAATACGGATGACGGGGCTCTTCAGACACGGCTGTATATTACTGTCCGAGACAGTCGACCATAGGGGGCTGAAGAGCCCTCTGTTATTGCCGCC |
| VHCHR3_1Phe | CCAATACGGATGACGGGGCTCTTCAGACACGGCTGTATATTACTGTTTTAGACAGTCGACCATAGGGGGCTGAAGAGCCCTCTGTTATTGCCGCC |
| VHCHR3_1Tyr | CCAATACGGATGACGGGGCTCTTCAGACACGGCTGTATATTACTGTTACAGACAGTCGACCATAGGGGGCTGAAGAGCCCTCTGTTATTGCCGCC |
| VHCHR3_1Trp | CCAATACGGATGACGGGGCTCTTCAGACACGGCTGTATATTACTGTTGGAGACAGTCGACCATAGGGGGCTGAAGAGCCCTCTGTTATTGCCGCC |
| VHCHR3_1Ser | CCAATACGGATGACGGGGCTCTTCAGACACGGCTGTATATTACTGTAGCAGACAGTCGACCATAGGGGGCTGAAGAGCCCTCTGTTATTGCCGCC |
| VHCHR3_1Thr | CCAATACGGATGACGGGGCTCTTCAGACACGGCTGTATATTACTGTACAAGACAGTCGACCATAGGGGGCTGAAGAGCCCTCTGTTATTGCCGCC |
| VHCHR3_1Cys | CCAATACGGATGACGGGGCTCTTCAGACACGGCTGTATATTACTGTTGTAGACAGTCGACCATAGGGGGCTGAAGAGCCCTCTGTTATTGCCGCC |
| VHCHR3_1Met | CCAATACGGATGACGGGGCTCTTCAGACACGGCTGTATATTACTGTATGAGACAGTCGACCATAGGGGGCTGAAGAGCCCTCTGTTATTGCCGCC |
| VHCHR3_1Asn | CCAATACGGATGACGGGGCTCTTCAGACACGGCTGTATATTACTGTAACAGACAGTCGACCATAGGGGGCTGAAGAGCCCTCTGTTATTGCCGCC |
| VHCHR3_1Gln | CCAATACGGATGACGGGGCTCTTCAGACACGGCTGTATATTACTGTCAGAGACAGTCGACCATAGGGGGCTGAAGAGCCCTCTGTTATTGCCGCC |
| VHCHR3_1Asp | CCAATACGGATGACGGGGCTCTTCAGACACGGCTGTATATTACTGTGACAGACAGTCGACCATAGGGGGCTGAAGAGCCCTCTGTTATTGCCGCC |
| VHCHR3_1Glu | CCAATACGGATGACGGGGCTCTTCAGACACGGCTGTATATTACTGTGAGAGACAGTCGACCATAGGGGGCTGAAGAGCCCTCTGTTATTGCCGCC |
| VHCHR3_1Lys | CCAATACGGATGACGGGGCTCTTCAGACACGGCTGTATATTACTGTAAAAGACAGTCGACCATAGGGGGCTGAAGAGCCCTCTGTTATTGCCGCC |
| VHCHR3_1Arg | CCAATACGGATGACGGGGCTCTTCAGACACGGCTGTATATTACTGTAGGAGACAGTCGACCATAGGGGGCTGAAGAGCCCTCTGTTATTGCCGCC |
| VHCHR3_1His | CCAATACGGATGACGGGGCTCTTCAGACACGGCTGTATATTACTGTCATAGACAGTCGACCATAGGGGGCTGAAGAGCCCTCTGTTATTGCCGCC |
| VHCHR3_2Gly | CCAATACGGATGACGGGGCTCTTCAACGGCTGTATATTACTGTGCGGGGCAGTCGACCATAGGGGGCTTCTGAAGAGCCCTCTGTTATTGCCGCC |
| VHCHR3_2Ala | CCAATACGGATGACGGGGCTCTTCAACGGCTGTATATTACTGTGCGGCTCAGTCGACCATAGGGGGCTTCTGAAGAGCCCTCTGTTATTGCCGCC |
| VHCHR3_2Val | CCAATACGGATGACGGGGCTCTTCAACGGCTGTATATTACTGTGCGGTACAGTCGACCATAGGGGGCTTCTGAAGAGCCCTCTGTTATTGCCGCC |
| VHCHR3_2Leu | CCAATACGGATGACGGGGCTCTTCAACGGCTGTATATTACTGTGCGTTACAGTCGACCATAGGGGGCTTCTGAAGAGCCCTCTGTTATTGCCGCC |
| VHCHR3_2Ile | CCAATACGGATGACGGGGCTCTTCAACGGCTGTATATTACTGTGCGATTCAGTCGACCATAGGGGGCTTCTGAAGAGCCCTCTGTTATTGCCGCC |
| VHCHR3_2Pro | CCAATACGGATGACGGGGCTCTTCAACGGCTGTATATTACTGTGCGCCACAGTCGACCATAGGGGGCTTCTGAAGAGCCCTCTGTTATTGCCGCC |
| VHCHR3_2Phe | CCAATACGGATGACGGGGCTCTTCAACGGCTGTATATTACTGTGCGTTCCAGTCGACCATAGGGGGCTTCTGAAGAGCCCTCTGTTATTGCCGCC |
| VHCHR3_2Tyr | CCAATACGGATGACGGGGCTCTTCAACGGCTGTATATTACTGTGCGTATCAGTCGACCATAGGGGGCTTCTGAAGAGCCCTCTGTTATTGCCGCC |
| VHCHR3_2Trp | CCAATACGGATGACGGGGCTCTTCAACGGCTGTATATTACTGTGCGTGGCAGTCGACCATAGGGGGCTTCTGAAGAGCCCTCTGTTATTGCCGCC |
| VHCHR3_2Ser | CCAATACGGATGACGGGGCTCTTCAACGGCTGTATATTACTGTGCGTCACAGTCGACCATAGGGGGCTTCTGAAGAGCCCTCTGTTATTGCCGCC |
| VHCHR3_2Thr | CCAATACGGATGACGGGGCTCTTCAACGGCTGTATATTACTGTGCGACGCAGTCGACCATAGGGGGCTTCTGAAGAGCCCTCTGTTATTGCCGCC |
| VHCHR3_2Cys | CCAATACGGATGACGGGGCTCTTCAACGGCTGTATATTACTGTGCGTGTCAGTCGACCATAGGGGGCTTCTGAAGAGCCCTCTGTTATTGCCGCC |
| VHCHR3_2Met | CCAATACGGATGACGGGGCTCTTCAACGGCTGTATATTACTGTGCGATGCAGTCGACCATAGGGGGCTTCTGAAGAGCCCTCTGTTATTGCCGCC |
| VHCHR3_2Asn | CCAATACGGATGACGGGGCTCTTCAACGGCTGTATATTACTGTGCGAATCAGTCGACCATAGGGGGCTTCTGAAGAGCCCTCTGTTATTGCCGCC |
| VHCHR3_2Gln | CCAATACGGATGACGGGGCTCTTCAACGGCTGTATATTACTGTGCGCAGCAGTCGACCATAGGGGGCTTCTGAAGAGCCCTCTGTTATTGCCGCC |
| VHCHR3_2Asp | CCAATACGGATGACGGGGCTCTTCAACGGCTGTATATTACTGTGCGGATCAGTCGACCATAGGGGGCTTCTGAAGAGCCCTCTGTTATTGCCGCC |
| VHCHR3_2Glu | CCAATACGGATGACGGGGCTCTTCAACGGCTGTATATTACTGTGCGGAGCAGTCGACCATAGGGGGCTTCTGAAGAGCCCTCTGTTATTGCCGCC |
| VHCHR3_2Lys | CCAATACGGATGACGGGGCTCTTCAACGGCTGTATATTACTGTGCGAAACAGTCGACCATAGGGGGCTTCTGAAGAGCCCTCTGTTATTGCCGCC |
| VHCHR3_2His | CCAATACGGATGACGGGGCTCTTCAACGGCTGTATATTACTGTGCGCATCAGTCGACCATAGGGGGCTTCTGAAGAGCCCTCTGTTATTGCCGCC |
| VHCHR3_3Gly | CCAATACGGATGACGGGGCTCTTCAGCTGTATATTACTGTGCGAGAGGCTCGACCATAGGGGGCTTCTTTTGAAGAGCCCTCTGTTATTGCCGCC |
| VHCHR3_3Ala | CCAATACGGATGACGGGGCTCTTCAGCTGTATATTACTGTGCGAGAGCTTCGACCATAGGGGGCTTCTTTTGAAGAGCCCTCTGTTATTGCCGCC |
| VHCHR3_3Val | CCAATACGGATGACGGGGCTCTTCAGCTGTATATTACTGTGCGAGAGTGTCGACCATAGGGGGCTTCTTTTGAAGAGCCCTCTGTTATTGCCGCC |
| VHCHR3_3Leu | CCAATACGGATGACGGGGCTCTTCAGCTGTATATTACTGTGCGAGACTGTCGACCATAGGGGGCTTCTTTTGAAGAGCCCTCTGTTATTGCCGCC |
| VHCHR3_3Ile | CCAATACGGATGACGGGGCTCTTCAGCTGTATATTACTGTGCGAGAATCTCGACCATAGGGGGCTTCTTTTGAAGAGCCCTCTGTTATTGCCGCC |
| VHCHR3_3Pro | CCAATACGGATGACGGGGCTCTTCAGCTGTATATTACTGTGCGAGACCTTCGACCATAGGGGGCTTCTTTTGAAGAGCCCTCTGTTATTGCCGCC |
| VHCHR3_3Phe | CCAATACGGATGACGGGGCTCTTCAGCTGTATATTACTGTGCGAGATTCTCGACCATAGGGGGCTTCTTTTGAAGAGCCCTCTGTTATTGCCGCC |
| VHCHR3_3Tyr | CCAATACGGATGACGGGGCTCTTCAGCTGTATATTACTGTGCGAGATATTCGACCATAGGGGGCTTCTTTTGAAGAGCCCTCTGTTATTGCCGCC |
| VHCHR3_3Trp | CCAATACGGATGACGGGGCTCTTCAGCTGTATATTACTGTGCGAGATGGTCGACCATAGGGGGCTTCTTTTGAAGAGCCCTCTGTTATTGCCGCC |
| VHCHR3_3Ser | CCAATACGGATGACGGGGCTCTTCAGCTGTATATTACTGTGCGAGATCCTCGACCATAGGGGGCTTCTTTTGAAGAGCCCTCTGTTATTGCCGCC |
| VHCHR3_3Thr | CCAATACGGATGACGGGGCTCTTCAGCTGTATATTACTGTGCGAGAACATCGACCATAGGGGGCTTCTTTTGAAGAGCCCTCTGTTATTGCCGCC |
| VHCHR3_3Cys | CCAATACGGATGACGGGGCTCTTCAGCTGTATATTACTGTGCGAGATGTTCGACCATAGGGGGCTTCTTTTGAAGAGCCCTCTGTTATTGCCGCC |
| VHCHR3_3Met | CCAATACGGATGACGGGGCTCTTCAGCTGTATATTACTGTGCGAGAATGTCGACCATAGGGGGCTTCTTTTGAAGAGCCCTCTGTTATTGCCGCC |
| VHCHR3_3Asn | CCAATACGGATGACGGGGCTCTTCAGCTGTATATTACTGTGCGAGAAATTCGACCATAGGGGGCTTCTTTTGAAGAGCCCTCTGTTATTGCCGCC |
| VHCHR3_3Asp | CCAATACGGATGACGGGGCTCTTCAGCTGTATATTACTGTGCGAGAGATTCGACCATAGGGGGCTTCTTTTGAAGAGCCCTCTGTTATTGCCGCC |
| VHCHR3_3Glu | CCAATACGGATGACGGGGCTCTTCAGCTGTATATTACTGTGCGAGAGAATCGACCATAGGGGGCTTCTTTTGAAGAGCCCTCTGTTATTGCCGCC |
| VHCHR3_3Lys | CCAATACGGATGACGGGGCTCTTCAGCTGTATATTACTGTGCGAGAAAATCGACCATAGGGGGCTTCTTTTGAAGAGCCCTCTGTTATTGCCGCC |
| VHCHR3_3Arg | CCAATACGGATGACGGGGCTCTTCAGCTGTATATTACTGTGCGAGACGCTCGACCATAGGGGGCTTCTTTTGAAGAGCCCTCTGTTATTGCCGCC |
| VHCHR3_3His | CCAATACGGATGACGGGGCTCTTCAGCTGTATATTACTGTGCGAGACATTCGACCATAGGGGGCTTCTTTTGAAGAGCCCTCTGTTATTGCCGCC |
| VHCHR3_4Gly | CCAATACGGATGACGGGGCTCTTCAGTATATTACTGTGCGAGACAGGGGACCATAGGGGGCTTCTTTGACTGAAGAGCCCTCTGTTATTGCCGCC |
| VHCHR3_4Ala | CCAATACGGATGACGGGGCTCTTCAGTATATTACTGTGCGAGACAGGCCACCATAGGGGGCTTCTTTGACTGAAGAGCCCTCTGTTATTGCCGCC |
| VHCHR3_4Val | CCAATACGGATGACGGGGCTCTTCAGTATATTACTGTGCGAGACAGGTTACCATAGGGGGCTTCTTTGACTGAAGAGCCCTCTGTTATTGCCGCC |
| VHCHR3_4Leu | CCAATACGGATGACGGGGCTCTTCAGTATATTACTGTGCGAGACAGTTGACCATAGGGGGCTTCTTTGACTGAAGAGCCCTCTGTTATTGCCGCC |
| VHCHR3_4Ile | CCAATACGGATGACGGGGCTCTTCAGTATATTACTGTGCGAGACAGATCACCATAGGGGGCTTCTTTGACTGAAGAGCCCTCTGTTATTGCCGCC |
| VHCHR3_4Pro | CCAATACGGATGACGGGGCTCTTCAGTATATTACTGTGCGAGACAGCCCACCATAGGGGGCTTCTTTGACTGAAGAGCCCTCTGTTATTGCCGCC |
| VHCHR3_4Phe | CCAATACGGATGACGGGGCTCTTCAGTATATTACTGTGCGAGACAGTTTACCATAGGGGGCTTCTTTGACTGAAGAGCCCTCTGTTATTGCCGCC |
| VHCHR3_4Tyr | CCAATACGGATGACGGGGCTCTTCAGTATATTACTGTGCGAGACAGTATACCATAGGGGGCTTCTTTGACTGAAGAGCCCTCTGTTATTGCCGCC |
| VHCHR3_4Trp | CCAATACGGATGACGGGGCTCTTCAGTATATTACTGTGCGAGACAGTGGACCATAGGGGGCTTCTTTGACTGAAGAGCCCTCTGTTATTGCCGCC |
| VHCHR3_4Thr | CCAATACGGATGACGGGGCTCTTCAGTATATTACTGTGCGAGACAGACAACCATAGGGGGCTTCTTTGACTGAAGAGCCCTCTGTTATTGCCGCC |
| VHCHR3_4Cys | CCAATACGGATGACGGGGCTCTTCAGTATATTACTGTGCGAGACAGTGTACCATAGGGGGCTTCTTTGACTGAAGAGCCCTCTGTTATTGCCGCC |
| VHCHR3_4Met | CCAATACGGATGACGGGGCTCTTCAGTATATTACTGTGCGAGACAGATGACCATAGGGGGCTTCTTTGACTGAAGAGCCCTCTGTTATTGCCGCC |
| VHCHR3_4Asn | CCAATACGGATGACGGGGCTCTTCAGTATATTACTGTGCGAGACAGAACACCATAGGGGGCTTCTTTGACTGAAGAGCCCTCTGTTATTGCCGCC |
| VHCHR3_4Gln | CCAATACGGATGACGGGGCTCTTCAGTATATTACTGTGCGAGACAGCAGACCATAGGGGGCTTCTTTGACTGAAGAGCCCTCTGTTATTGCCGCC |
| VHCHR3_4Asp | CCAATACGGATGACGGGGCTCTTCAGTATATTACTGTGCGAGACAGGATACCATAGGGGGCTTCTTTGACTGAAGAGCCCTCTGTTATTGCCGCC |
| VHCHR3_4Glu | CCAATACGGATGACGGGGCTCTTCAGTATATTACTGTGCGAGACAGGAGACCATAGGGGGCTTCTTTGACTGAAGAGCCCTCTGTTATTGCCGCC |
| VHCHR3_4Lys | CCAATACGGATGACGGGGCTCTTCAGTATATTACTGTGCGAGACAGAAGACCATAGGGGGCTTCTTTGACTGAAGAGCCCTCTGTTATTGCCGCC |
| VHCHR3_4Arg | CCAATACGGATGACGGGGCTCTTCAGTATATTACTGTGCGAGACAGCGGACCATAGGGGGCTTCTTTGACTGAAGAGCCCTCTGTTATTGCCGCC |
| VHCHR3_4His | CCAATACGGATGACGGGGCTCTTCAGTATATTACTGTGCGAGACAGCACACCATAGGGGGCTTCTTTGACTGAAGAGCCCTCTGTTATTGCCGCC |
| VHCHR3_5Gly | CCAATACGGATGACGGGGCTCTTCATATTACTGTGCGAGACAGTCGGGGATAGGGGGCTTCTTTGACTACTGAAGAGCCCTCTGTTATTGCCGCC |
| VHCHR3_5Ala | CCAATACGGATGACGGGGCTCTTCATATTACTGTGCGAGACAGTCGGCTATAGGGGGCTTCTTTGACTACTGAAGAGCCCTCTGTTATTGCCGCC |
| VHCHR3_5Val | CCAATACGGATGACGGGGCTCTTCATATTACTGTGCGAGACAGTCGGTGATAGGGGGCTTCTTTGACTACTGAAGAGCCCTCTGTTATTGCCGCC |
| VHCHR3_5Leu | CCAATACGGATGACGGGGCTCTTCATATTACTGTGCGAGACAGTCGTTGATAGGGGGCTTCTTTGACTACTGAAGAGCCCTCTGTTATTGCCGCC |
| VHCHR3_5Ile | CCAATACGGATGACGGGGCTCTTCATATTACTGTGCGAGACAGTCGATCATAGGGGGCTTCTTTGACTACTGAAGAGCCCTCTGTTATTGCCGCC |
| VHCHR3_5Pro | CCAATACGGATGACGGGGCTCTTCATATTACTGTGCGAGACAGTCGCCCATAGGGGGCTTCTTTGACTACTGAAGAGCCCTCTGTTATTGCCGCC |
| VHCHR3_5Phe | CCAATACGGATGACGGGGCTCTTCATATTACTGTGCGAGACAGTCGTTTATAGGGGGCTTCTTTGACTACTGAAGAGCCCTCTGTTATTGCCGCC |
| VHCHR3_5Tyr | CCAATACGGATGACGGGGCTCTTCATATTACTGTGCGAGACAGTCGTATATAGGGGGCTTCTTTGACTACTGAAGAGCCCTCTGTTATTGCCGCC |
| VHCHR3_5Trp | CCAATACGGATGACGGGGCTCTTCATATTACTGTGCGAGACAGTCGTGGATAGGGGGCTTCTTTGACTACTGAAGAGCCCTCTGTTATTGCCGCC |
| VHCHR3_5Ser | CCAATACGGATGACGGGGCTCTTCATATTACTGTGCGAGACAGTCGTCTATAGGGGGCTTCTTTGACTACTGAAGAGCCCTCTGTTATTGCCGCC |
| VHCHR3_5Cys | CCAATACGGATGACGGGGCTCTTCATATTACTGTGCGAGACAGTCGTGCATAGGGGGCTTCTTTGACTACTGAAGAGCCCTCTGTTATTGCCGCC |
| VHCHR3_5Met | CCAATACGGATGACGGGGCTCTTCATATTACTGTGCGAGACAGTCGATGATAGGGGGCTTCTTTGACTACTGAAGAGCCCTCTGTTATTGCCGCC |
| VHCHR3_5Asn | CCAATACGGATGACGGGGCTCTTCATATTACTGTGCGAGACAGTCGAATATAGGGGGCTTCTTTGACTACTGAAGAGCCCTCTGTTATTGCCGCC |
| VHCHR3_5Gln | CCAATACGGATGACGGGGCTCTTCATATTACTGTGCGAGACAGTCGCAAATAGGGGGCTTCTTTGACTACTGAAGAGCCCTCTGTTATTGCCGCC |
| VHCHR3_5Asp | CCAATACGGATGACGGGGCTCTTCATATTACTGTGCGAGACAGTCGGATATAGGGGGCTTCTTTGACTACTGAAGAGCCCTCTGTTATTGCCGCC |
| VHCHR3_5Glu | CCAATACGGATGACGGGGCTCTTCATATTACTGTGCGAGACAGTCGGAAATAGGGGGCTTCTTTGACTACTGAAGAGCCCTCTGTTATTGCCGCC |
| VHCHR3_5Lys | CCAATACGGATGACGGGGCTCTTCATATTACTGTGCGAGACAGTCGAAGATAGGGGGCTTCTTTGACTACTGAAGAGCCCTCTGTTATTGCCGCC |
| VHCHR3_5Arg | CCAATACGGATGACGGGGCTCTTCATATTACTGTGCGAGACAGTCGCGCATAGGGGGCTTCTTTGACTACTGAAGAGCCCTCTGTTATTGCCGCC |
| VHCHR3_5His | CCAATACGGATGACGGGGCTCTTCATATTACTGTGCGAGACAGTCGCATATAGGGGGCTTCTTTGACTACTGAAGAGCCCTCTGTTATTGCCGCC |
| VHCHR3_6Gly | CCAATACGGATGACGGGGCTCTTCATACTGTGCGAGACAGTCGACCGGCGGGGGCTTCTTTGACTACTGGTGAAGAGCCCTCTGTTATTGCCGCC |
| VHCHR3_6Ala | CCAATACGGATGACGGGGCTCTTCATACTGTGCGAGACAGTCGACCGCGGGGGGCTTCTTTGACTACTGGTGAAGAGCCCTCTGTTATTGCCGCC |
| VHCHR3_6Val | CCAATACGGATGACGGGGCTCTTCATACTGTGCGAGACAGTCGACCGTTGGGGGCTTCTTTGACTACTGGTGAAGAGCCCTCTGTTATTGCCGCC |
| VHCHR3_6Leu | CCAATACGGATGACGGGGCTCTTCATACTGTGCGAGACAGTCGACCTTAGGGGGCTTCTTTGACTACTGGTGAAGAGCCCTCTGTTATTGCCGCC |
| VHCHR3_6Pro | CCAATACGGATGACGGGGCTCTTCATACTGTGCGAGACAGTCGACCCCCGGGGGCTTCTTTGACTACTGGTGAAGAGCCCTCTGTTATTGCCGCC |
| VHCHR3_6Phe | CCAATACGGATGACGGGGCTCTTCATACTGTGCGAGACAGTCGACCTTTGGGGGCTTCTTTGACTACTGGTGAAGAGCCCTCTGTTATTGCCGCC |
| VHCHR3_6Tyr | CCAATACGGATGACGGGGCTCTTCATACTGTGCGAGACAGTCGACCTATGGGGGCTTCTTTGACTACTGGTGAAGAGCCCTCTGTTATTGCCGCC |
| VHCHR3_6Trp | CCAATACGGATGACGGGGCTCTTCATACTGTGCGAGACAGTCGACCTGGGGGGGCTTCTTTGACTACTGGTGAAGAGCCCTCTGTTATTGCCGCC |
| VHCHR3_6Ser | CCAATACGGATGACGGGGCTCTTCATACTGTGCGAGACAGTCGACCAGCGGGGGCTTCTTTGACTACTGGTGAAGAGCCCTCTGTTATTGCCGCC |
| VHCHR3_6Thr | CCAATACGGATGACGGGGCTCTTCATACTGTGCGAGACAGTCGACCACGGGGGGCTTCTTTGACTACTGGTGAAGAGCCCTCTGTTATTGCCGCC |
| VHCHR3_6Cys | CCAATACGGATGACGGGGCTCTTCATACTGTGCGAGACAGTCGACCTGCGGGGGCTTCTTTGACTACTGGTGAAGAGCCCTCTGTTATTGCCGCC |
| VHCHR3_6Met | CCAATACGGATGACGGGGCTCTTCATACTGTGCGAGACAGTCGACCATGGGGGGCTTCTTTGACTACTGGTGAAGAGCCCTCTGTTATTGCCGCC |
| VHCHR3_6Asn | CCAATACGGATGACGGGGCTCTTCATACTGTGCGAGACAGTCGACCAACGGGGGCTTCTTTGACTACTGGTGAAGAGCCCTCTGTTATTGCCGCC |
| VHCHR3_6Gln | CCAATACGGATGACGGGGCTCTTCATACTGTGCGAGACAGTCGACCCAAGGGGGCTTCTTTGACTACTGGTGAAGAGCCCTCTGTTATTGCCGCC |
| VHCHR3_6Asp | CCAATACGGATGACGGGGCTCTTCATACTGTGCGAGACAGTCGACCGATGGGGGCTTCTTTGACTACTGGTGAAGAGCCCTCTGTTATTGCCGCC |
| VHCHR3_6Glu | CCAATACGGATGACGGGGCTCTTCATACTGTGCGAGACAGTCGACCGAGGGGGGCTTCTTTGACTACTGGTGAAGAGCCCTCTGTTATTGCCGCC |
| VHCHR3_6Lys | CCAATACGGATGACGGGGCTCTTCATACTGTGCGAGACAGTCGACCAAAGGGGGCTTCTTTGACTACTGGTGAAGAGCCCTCTGTTATTGCCGCC |
| VHCHR3_6Arg | CCAATACGGATGACGGGGCTCTTCATACTGTGCGAGACAGTCGACCCGAGGGGGCTTCTTTGACTACTGGTGAAGAGCCCTCTGTTATTGCCGCC |
| VHCHR3_6His | CCAATACGGATGACGGGGCTCTTCATACTGTGCGAGACAGTCGACCCACGGGGGCTTCTTTGACTACTGGTGAAGAGCCCTCTGTTATTGCCGCC |
| VHCHR3_7Ala | CCAATACGGATGACGGGGCTCTTCATGTGCGAGACAGTCGACCATAGCTGGCTTCTTTGACTACTGGGGCTGAAGAGCCCTCTGTTATTGCCGCC |
| VHCHR3_7Val | CCAATACGGATGACGGGGCTCTTCATGTGCGAGACAGTCGACCATAGTTGGCTTCTTTGACTACTGGGGCTGAAGAGCCCTCTGTTATTGCCGCC |
| VHCHR3_7Leu | CCAATACGGATGACGGGGCTCTTCATGTGCGAGACAGTCGACCATACTTGGCTTCTTTGACTACTGGGGCTGAAGAGCCCTCTGTTATTGCCGCC |
| VHCHR3_7Ile | CCAATACGGATGACGGGGCTCTTCATGTGCGAGACAGTCGACCATAATCGGCTTCTTTGACTACTGGGGCTGAAGAGCCCTCTGTTATTGCCGCC |
| VHCHR3_7Pro | CCAATACGGATGACGGGGCTCTTCATGTGCGAGACAGTCGACCATACCAGGCTTCTTTGACTACTGGGGCTGAAGAGCCCTCTGTTATTGCCGCC |
| VHCHR3_7Phe | CCAATACGGATGACGGGGCTCTTCATGTGCGAGACAGTCGACCATATTTGGCTTCTTTGACTACTGGGGCTGAAGAGCCCTCTGTTATTGCCGCC |
| VHCHR3_7Tyr | CCAATACGGATGACGGGGCTCTTCATGTGCGAGACAGTCGACCATATATGGCTTCTTTGACTACTGGGGCTGAAGAGCCCTCTGTTATTGCCGCC |
| VHCHR3_7Trp | CCAATACGGATGACGGGGCTCTTCATGTGCGAGACAGTCGACCATATGGGGCTTCTTTGACTACTGGGGCTGAAGAGCCCTCTGTTATTGCCGCC |
| VHCHR3_7Ser | CCAATACGGATGACGGGGCTCTTCATGTGCGAGACAGTCGACCATATCAGGCTTCTTTGACTACTGGGGCTGAAGAGCCCTCTGTTATTGCCGCC |
| VHCHR3_7Thr | CCAATACGGATGACGGGGCTCTTCATGTGCGAGACAGTCGACCATAACGGGCTTCTTTGACTACTGGGGCTGAAGAGCCCTCTGTTATTGCCGCC |
| VHCHR3_7Cys | CCAATACGGATGACGGGGCTCTTCATGTGCGAGACAGTCGACCATATGTGGCTTCTTTGACTACTGGGGCTGAAGAGCCCTCTGTTATTGCCGCC |
| VHCHR3_7Met | CCAATACGGATGACGGGGCTCTTCATGTGCGAGACAGTCGACCATAATGGGCTTCTTTGACTACTGGGGCTGAAGAGCCCTCTGTTATTGCCGCC |
| VHCHR3_7Asn | CCAATACGGATGACGGGGCTCTTCATGTGCGAGACAGTCGACCATAAACGGCTTCTTTGACTACTGGGGCTGAAGAGCCCTCTGTTATTGCCGCC |
| VHCHR3_7Gln | CCAATACGGATGACGGGGCTCTTCATGTGCGAGACAGTCGACCATACAGGGCTTCTTTGACTACTGGGGCTGAAGAGCCCTCTGTTATTGCCGCC |
| VHCHR3_7Asp | CCAATACGGATGACGGGGCTCTTCATGTGCGAGACAGTCGACCATAGACGGCTTCTTTGACTACTGGGGCTGAAGAGCCCTCTGTTATTGCCGCC |
| VHCHR3_7Glu | CCAATACGGATGACGGGGCTCTTCATGTGCGAGACAGTCGACCATAGAGGGCTTCTTTGACTACTGGGGCTGAAGAGCCCTCTGTTATTGCCGCC |
| VHCHR3_7Lys | CCAATACGGATGACGGGGCTCTTCATGTGCGAGACAGTCGACCATAAAAGGCTTCTTTGACTACTGGGGCTGAAGAGCCCTCTGTTATTGCCGCC |
| VHCHR3_7Arg | CCAATACGGATGACGGGGCTCTTCATGTGCGAGACAGTCGACCATACGCGGCTTCTTTGACTACTGGGGCTGAAGAGCCCTCTGTTATTGCCGCC |
| VHCHR3_7His | CCAATACGGATGACGGGGCTCTTCATGTGCGAGACAGTCGACCATACATGGCTTCTTTGACTACTGGGGCTGAAGAGCCCTCTGTTATTGCCGCC |
| VHCHR3_8Ala | CCAATACGGATGACGGGGCTCTTCAGCGAGACAGTCGACCATAGGGGCATTCTTTGACTACTGGGGCCAGTGAAGAGCCCTCTGTTATTGCCGCC |
| VHCHR3_8Val | CCAATACGGATGACGGGGCTCTTCAGCGAGACAGTCGACCATAGGGGTATTCTTTGACTACTGGGGCCAGTGAAGAGCCCTCTGTTATTGCCGCC |
| VHCHR3_8Leu | CCAATACGGATGACGGGGCTCTTCAGCGAGACAGTCGACCATAGGGCTGTTCTTTGACTACTGGGGCCAGTGAAGAGCCCTCTGTTATTGCCGCC |
| VHCHR3_8Ile | CCAATACGGATGACGGGGCTCTTCAGCGAGACAGTCGACCATAGGGATCTTCTTTGACTACTGGGGCCAGTGAAGAGCCCTCTGTTATTGCCGCC |
| VHCHR3_8Pro | CCAATACGGATGACGGGGCTCTTCAGCGAGACAGTCGACCATAGGGCCGTTCTTTGACTACTGGGGCCAGTGAAGAGCCCTCTGTTATTGCCGCC |
| VHCHR3_8Phe | CCAATACGGATGACGGGGCTCTTCAGCGAGACAGTCGACCATAGGGTTCTTCTTTGACTACTGGGGCCAGTGAAGAGCCCTCTGTTATTGCCGCC |
| VHCHR3_8Tyr | CCAATACGGATGACGGGGCTCTTCAGCGAGACAGTCGACCATAGGGTACTTCTTTGACTACTGGGGCCAGTGAAGAGCCCTCTGTTATTGCCGCC |
| VHCHR3_8Trp | CCAATACGGATGACGGGGCTCTTCAGCGAGACAGTCGACCATAGGGTGGTTCTTTGACTACTGGGGCCAGTGAAGAGCCCTCTGTTATTGCCGCC |
| VHCHR3_8Ser | CCAATACGGATGACGGGGCTCTTCAGCGAGACAGTCGACCATAGGGAGTTTCTTTGACTACTGGGGCCAGTGAAGAGCCCTCTGTTATTGCCGCC |
| VHCHR3_8Thr | CCAATACGGATGACGGGGCTCTTCAGCGAGACAGTCGACCATAGGGACATTCTTTGACTACTGGGGCCAGTGAAGAGCCCTCTGTTATTGCCGCC |
| VHCHR3_8Cys | CCAATACGGATGACGGGGCTCTTCAGCGAGACAGTCGACCATAGGGTGTTTCTTTGACTACTGGGGCCAGTGAAGAGCCCTCTGTTATTGCCGCC |
| VHCHR3_8Met | CCAATACGGATGACGGGGCTCTTCAGCGAGACAGTCGACCATAGGGATGTTCTTTGACTACTGGGGCCAGTGAAGAGCCCTCTGTTATTGCCGCC |
| VHCHR3_8Asn | CCAATACGGATGACGGGGCTCTTCAGCGAGACAGTCGACCATAGGGAACTTCTTTGACTACTGGGGCCAGTGAAGAGCCCTCTGTTATTGCCGCC |
| VHCHR3_8Gln | CCAATACGGATGACGGGGCTCTTCAGCGAGACAGTCGACCATAGGGCAGTTCTTTGACTACTGGGGCCAGTGAAGAGCCCTCTGTTATTGCCGCC |
| VHCHR3_8Asp | CCAATACGGATGACGGGGCTCTTCAGCGAGACAGTCGACCATAGGGGACTTCTTTGACTACTGGGGCCAGTGAAGAGCCCTCTGTTATTGCCGCC |
| VHCHR3_8Glu | CCAATACGGATGACGGGGCTCTTCAGCGAGACAGTCGACCATAGGGGAGTTCTTTGACTACTGGGGCCAGTGAAGAGCCCTCTGTTATTGCCGCC |
| VHCHR3_8Lys | CCAATACGGATGACGGGGCTCTTCAGCGAGACAGTCGACCATAGGGAAGTTCTTTGACTACTGGGGCCAGTGAAGAGCCCTCTGTTATTGCCGCC |
| VHCHR3_8Arg | CCAATACGGATGACGGGGCTCTTCAGCGAGACAGTCGACCATAGGGAGGTTCTTTGACTACTGGGGCCAGTGAAGAGCCCTCTGTTATTGCCGCC |
| VHCHR3_8His | CCAATACGGATGACGGGGCTCTTCAGCGAGACAGTCGACCATAGGGCATTTCTTTGACTACTGGGGCCAGTGAAGAGCCCTCTGTTATTGCCGCC |
| VHCHR3_9Gly | CCAATACGGATGACGGGGCTCTTCAAGACAGTCGACCATAGGGGGCGGGTTTGACTACTGGGGCCAGGGATGAAGAGCCCTCTGTTATTGCCGCC |
| VHCHR3_9Ala | CCAATACGGATGACGGGGCTCTTCAAGACAGTCGACCATAGGGGGCGCTTTTGACTACTGGGGCCAGGGATGAAGAGCCCTCTGTTATTGCCGCC |
| VHCHR3_9Val | CCAATACGGATGACGGGGCTCTTCAAGACAGTCGACCATAGGGGGCGTATTTGACTACTGGGGCCAGGGATGAAGAGCCCTCTGTTATTGCCGCC |
| VHCHR3_9Leu | CCAATACGGATGACGGGGCTCTTCAAGACAGTCGACCATAGGGGGCCTCTTTGACTACTGGGGCCAGGGATGAAGAGCCCTCTGTTATTGCCGCC |
| VHCHR3_9Ile | CCAATACGGATGACGGGGCTCTTCAAGACAGTCGACCATAGGGGGCATCTTTGACTACTGGGGCCAGGGATGAAGAGCCCTCTGTTATTGCCGCC |
| VHCHR3_9Pro | CCAATACGGATGACGGGGCTCTTCAAGACAGTCGACCATAGGGGGCCCGTTTGACTACTGGGGCCAGGGATGAAGAGCCCTCTGTTATTGCCGCC |
| VHCHR3_9Tyr | CCAATACGGATGACGGGGCTCTTCAAGACAGTCGACCATAGGGGGCTACTTTGACTACTGGGGCCAGGGATGAAGAGCCCTCTGTTATTGCCGCC |
| VHCHR3_9Trp | CCAATACGGATGACGGGGCTCTTCAAGACAGTCGACCATAGGGGGCTGGTTTGACTACTGGGGCCAGGGATGAAGAGCCCTCTGTTATTGCCGCC |
| VHCHR3_9Ser | CCAATACGGATGACGGGGCTCTTCAAGACAGTCGACCATAGGGGGCTCGTTTGACTACTGGGGCCAGGGATGAAGAGCCCTCTGTTATTGCCGCC |
| VHCHR3_9Thr | CCAATACGGATGACGGGGCTCTTCAAGACAGTCGACCATAGGGGGCACGTTTGACTACTGGGGCCAGGGATGAAGAGCCCTCTGTTATTGCCGCC |
| VHCHR3_9Cys | CCAATACGGATGACGGGGCTCTTCAAGACAGTCGACCATAGGGGGCTGTTTTGACTACTGGGGCCAGGGATGAAGAGCCCTCTGTTATTGCCGCC |
| VHCHR3_9Met | CCAATACGGATGACGGGGCTCTTCAAGACAGTCGACCATAGGGGGCATGTTTGACTACTGGGGCCAGGGATGAAGAGCCCTCTGTTATTGCCGCC |
| VHCHR3_9Asn | CCAATACGGATGACGGGGCTCTTCAAGACAGTCGACCATAGGGGGCAATTTTGACTACTGGGGCCAGGGATGAAGAGCCCTCTGTTATTGCCGCC |
| VHCHR3_9Gln | CCAATACGGATGACGGGGCTCTTCAAGACAGTCGACCATAGGGGGCCAATTTGACTACTGGGGCCAGGGATGAAGAGCCCTCTGTTATTGCCGCC |
| VHCHR3_9Asp | CCAATACGGATGACGGGGCTCTTCAAGACAGTCGACCATAGGGGGCGATTTTGACTACTGGGGCCAGGGATGAAGAGCCCTCTGTTATTGCCGCC |
| VHCHR3_9Glu | CCAATACGGATGACGGGGCTCTTCAAGACAGTCGACCATAGGGGGCGAATTTGACTACTGGGGCCAGGGATGAAGAGCCCTCTGTTATTGCCGCC |
| VHCHR3_9Lys | CCAATACGGATGACGGGGCTCTTCAAGACAGTCGACCATAGGGGGCAAATTTGACTACTGGGGCCAGGGATGAAGAGCCCTCTGTTATTGCCGCC |
| VHCHR3_9Arg | CCAATACGGATGACGGGGCTCTTCAAGACAGTCGACCATAGGGGGCCGGTTTGACTACTGGGGCCAGGGATGAAGAGCCCTCTGTTATTGCCGCC |
| VHCHR3_9His | CCAATACGGATGACGGGGCTCTTCAAGACAGTCGACCATAGGGGGCCATTTTGACTACTGGGGCCAGGGATGAAGAGCCCTCTGTTATTGCCGCC |
| VHCHR3_10Gly | CCAATACGGATGACGGGGCTCTTCACAGTCGACCATAGGGGGCTTCGGCGACTACTGGGGCCAGGGAACCTGAAGAGCCCTCTGTTATTGCCGCC |
| VHCHR3_10Ala | CCAATACGGATGACGGGGCTCTTCACAGTCGACCATAGGGGGCTTCGCTGACTACTGGGGCCAGGGAACCTGAAGAGCCCTCTGTTATTGCCGCC |
| VHCHR3_10Val | CCAATACGGATGACGGGGCTCTTCACAGTCGACCATAGGGGGCTTCGTCGACTACTGGGGCCAGGGAACCTGAAGAGCCCTCTGTTATTGCCGCC |
| VHCHR3_10Leu | CCAATACGGATGACGGGGCTCTTCACAGTCGACCATAGGGGGCTTCCTGGACTACTGGGGCCAGGGAACCTGAAGAGCCCTCTGTTATTGCCGCC |
| VHCHR3_10Ile | CCAATACGGATGACGGGGCTCTTCACAGTCGACCATAGGGGGCTTCATCGACTACTGGGGCCAGGGAACCTGAAGAGCCCTCTGTTATTGCCGCC |
| VHCHR3_10Pro | CCAATACGGATGACGGGGCTCTTCACAGTCGACCATAGGGGGCTTCCCAGACTACTGGGGCCAGGGAACCTGAAGAGCCCTCTGTTATTGCCGCC |
| VHCHR3_10Tyr | CCAATACGGATGACGGGGCTCTTCACAGTCGACCATAGGGGGCTTCTACGACTACTGGGGCCAGGGAACCTGAAGAGCCCTCTGTTATTGCCGCC |
| VHCHR3_10Trp | CCAATACGGATGACGGGGCTCTTCACAGTCGACCATAGGGGGCTTCTGGGACTACTGGGGCCAGGGAACCTGAAGAGCCCTCTGTTATTGCCGCC |
| VHCHR3_10Ser | CCAATACGGATGACGGGGCTCTTCACAGTCGACCATAGGGGGCTTCAGCGACTACTGGGGCCAGGGAACCTGAAGAGCCCTCTGTTATTGCCGCC |
| VHCHR3_10Thr | CCAATACGGATGACGGGGCTCTTCACAGTCGACCATAGGGGGCTTCACGGACTACTGGGGCCAGGGAACCTGAAGAGCCCTCTGTTATTGCCGCC |
| VHCHR3_10Cys | CCAATACGGATGACGGGGCTCTTCACAGTCGACCATAGGGGGCTTCTGCGACTACTGGGGCCAGGGAACCTGAAGAGCCCTCTGTTATTGCCGCC |
| VHCHR3_10Met | CCAATACGGATGACGGGGCTCTTCACAGTCGACCATAGGGGGCTTCATGGACTACTGGGGCCAGGGAACCTGAAGAGCCCTCTGTTATTGCCGCC |
| VHCHR3_10Asn | CCAATACGGATGACGGGGCTCTTCACAGTCGACCATAGGGGGCTTCAACGACTACTGGGGCCAGGGAACCTGAAGAGCCCTCTGTTATTGCCGCC |
| VHCHR3_10Gln | CCAATACGGATGACGGGGCTCTTCACAGTCGACCATAGGGGGCTTCCAAGACTACTGGGGCCAGGGAACCTGAAGAGCCCTCTGTTATTGCCGCC |
| VHCHR3_10Asp | CCAATACGGATGACGGGGCTCTTCACAGTCGACCATAGGGGGCTTCGATGACTACTGGGGCCAGGGAACCTGAAGAGCCCTCTGTTATTGCCGCC |
| VHCHR3_10Glu | CCAATACGGATGACGGGGCTCTTCACAGTCGACCATAGGGGGCTTCGAGGACTACTGGGGCCAGGGAACCTGAAGAGCCCTCTGTTATTGCCGCC |
| VHCHR3_10Lys | CCAATACGGATGACGGGGCTCTTCACAGTCGACCATAGGGGGCTTCAAGGACTACTGGGGCCAGGGAACCTGAAGAGCCCTCTGTTATTGCCGCC |
| VHCHR3_10Arg | CCAATACGGATGACGGGGCTCTTCACAGTCGACCATAGGGGGCTTCAGGGACTACTGGGGCCAGGGAACCTGAAGAGCCCTCTGTTATTGCCGCC |
| VHCHR3_10His | CCAATACGGATGACGGGGCTCTTCACAGTCGACCATAGGGGGCTTCCATGACTACTGGGGCCAGGGAACCTGAAGAGCCCTCTGTTATTGCCGCC |
| VHCHR3_11Gly | CCAATACGGATGACGGGGCTCTTCATCGACCATAGGGGGCTTCTTTGGTTACTGGGGCCAGGGAACCCTGTGAAGAGCCCTCTGTTATTGCCGCC |
| VHCHR3_11Ala | CCAATACGGATGACGGGGCTCTTCATCGACCATAGGGGGCTTCTTTGCATACTGGGGCCAGGGAACCCTGTGAAGAGCCCTCTGTTATTGCCGCC |
| VHCHR3_11Val | CCAATACGGATGACGGGGCTCTTCATCGACCATAGGGGGCTTCTTTGTATACTGGGGCCAGGGAACCCTGTGAAGAGCCCTCTGTTATTGCCGCC |
| VHCHR3_11Leu | CCAATACGGATGACGGGGCTCTTCATCGACCATAGGGGGCTTCTTTCTCTACTGGGGCCAGGGAACCCTGTGAAGAGCCCTCTGTTATTGCCGCC |
| VHCHR3_11Ile | CCAATACGGATGACGGGGCTCTTCATCGACCATAGGGGGCTTCTTTATATACTGGGGCCAGGGAACCCTGTGAAGAGCCCTCTGTTATTGCCGCC |
| VHCHR3_11Pro | CCAATACGGATGACGGGGCTCTTCATCGACCATAGGGGGCTTCTTTCCCTACTGGGGCCAGGGAACCCTGTGAAGAGCCCTCTGTTATTGCCGCC |
| VHCHR3_11Phe | CCAATACGGATGACGGGGCTCTTCATCGACCATAGGGGGCTTCTTTTTTTACTGGGGCCAGGGAACCCTGTGAAGAGCCCTCTGTTATTGCCGCC |
| VHCHR3_11Tyr | CCAATACGGATGACGGGGCTCTTCATCGACCATAGGGGGCTTCTTTTATTACTGGGGCCAGGGAACCCTGTGAAGAGCCCTCTGTTATTGCCGCC |
| VHCHR3_11Trp | CCAATACGGATGACGGGGCTCTTCATCGACCATAGGGGGCTTCTTTTGGTACTGGGGCCAGGGAACCCTGTGAAGAGCCCTCTGTTATTGCCGCC |
| VHCHR3_11Ser | CCAATACGGATGACGGGGCTCTTCATCGACCATAGGGGGCTTCTTTAGTTACTGGGGCCAGGGAACCCTGTGAAGAGCCCTCTGTTATTGCCGCC |
| VHCHR3_11Thr | CCAATACGGATGACGGGGCTCTTCATCGACCATAGGGGGCTTCTTTACGTACTGGGGCCAGGGAACCCTGTGAAGAGCCCTCTGTTATTGCCGCC |
| VHCHR3_11Cys | CCAATACGGATGACGGGGCTCTTCATCGACCATAGGGGGCTTCTTTTGCTACTGGGGCCAGGGAACCCTGTGAAGAGCCCTCTGTTATTGCCGCC |
| VHCHR3_11Met | CCAATACGGATGACGGGGCTCTTCATCGACCATAGGGGGCTTCTTTATGTACTGGGGCCAGGGAACCCTGTGAAGAGCCCTCTGTTATTGCCGCC |
| VHCHR3_11Asn | CCAATACGGATGACGGGGCTCTTCATCGACCATAGGGGGCTTCTTTAATTACTGGGGCCAGGGAACCCTGTGAAGAGCCCTCTGTTATTGCCGCC |
| VHCHR3_11Gln | CCAATACGGATGACGGGGCTCTTCATCGACCATAGGGGGCTTCTTTCAATACTGGGGCCAGGGAACCCTGTGAAGAGCCCTCTGTTATTGCCGCC |
| VHCHR3_11Glu | CCAATACGGATGACGGGGCTCTTCATCGACCATAGGGGGCTTCTTTGAGTACTGGGGCCAGGGAACCCTGTGAAGAGCCCTCTGTTATTGCCGCC |
| VHCHR3_11Lys | CCAATACGGATGACGGGGCTCTTCATCGACCATAGGGGGCTTCTTTAAATACTGGGGCCAGGGAACCCTGTGAAGAGCCCTCTGTTATTGCCGCC |
| VHCHR3_11Arg | CCAATACGGATGACGGGGCTCTTCATCGACCATAGGGGGCTTCTTTCGCTACTGGGGCCAGGGAACCCTGTGAAGAGCCCTCTGTTATTGCCGCC |
| VHCHR3_11His | CCAATACGGATGACGGGGCTCTTCATCGACCATAGGGGGCTTCTTTCATTACTGGGGCCAGGGAACCCTGTGAAGAGCCCTCTGTTATTGCCGCC |
| VHCHR3_12Gly | CCAATACGGATGACGGGGCTCTTCAACCATAGGGGGCTTCTTTGACGGGTGGGGCCAGGGAACCCTGGTCTGAAGAGCCCTCTGTTATTGCCGCC |
| VHCHR3_12Ala | CCAATACGGATGACGGGGCTCTTCAACCATAGGGGGCTTCTTTGACGCTTGGGGCCAGGGAACCCTGGTCTGAAGAGCCCTCTGTTATTGCCGCC |
| VHCHR3_12Val | CCAATACGGATGACGGGGCTCTTCAACCATAGGGGGCTTCTTTGACGTGTGGGGCCAGGGAACCCTGGTCTGAAGAGCCCTCTGTTATTGCCGCC |
| VHCHR3_12Leu | CCAATACGGATGACGGGGCTCTTCAACCATAGGGGGCTTCTTTGACCTATGGGGCCAGGGAACCCTGGTCTGAAGAGCCCTCTGTTATTGCCGCC |
| VHCHR3_12Ile | CCAATACGGATGACGGGGCTCTTCAACCATAGGGGGCTTCTTTGACATTTGGGGCCAGGGAACCCTGGTCTGAAGAGCCCTCTGTTATTGCCGCC |
| VHCHR3_12Pro | CCAATACGGATGACGGGGCTCTTCAACCATAGGGGGCTTCTTTGACCCTTGGGGCCAGGGAACCCTGGTCTGAAGAGCCCTCTGTTATTGCCGCC |
| VHCHR3_12Phe | CCAATACGGATGACGGGGCTCTTCAACCATAGGGGGCTTCTTTGACTTCTGGGGCCAGGGAACCCTGGTCTGAAGAGCCCTCTGTTATTGCCGCC |
| VHCHR3_12Trp | CCAATACGGATGACGGGGCTCTTCAACCATAGGGGGCTTCTTTGACTGGTGGGGCCAGGGAACCCTGGTCTGAAGAGCCCTCTGTTATTGCCGCC |
| VHCHR3_12Ser | CCAATACGGATGACGGGGCTCTTCAACCATAGGGGGCTTCTTTGACAGTTGGGGCCAGGGAACCCTGGTCTGAAGAGCCCTCTGTTATTGCCGCC |
| VHCHR3_12Thr | CCAATACGGATGACGGGGCTCTTCAACCATAGGGGGCTTCTTTGACACTTGGGGCCAGGGAACCCTGGTCTGAAGAGCCCTCTGTTATTGCCGCC |
| VHCHR3_12Cys | CCAATACGGATGACGGGGCTCTTCAACCATAGGGGGCTTCTTTGACTGCTGGGGCCAGGGAACCCTGGTCTGAAGAGCCCTCTGTTATTGCCGCC |
| VHCHR3_12Met | CCAATACGGATGACGGGGCTCTTCAACCATAGGGGGCTTCTTTGACATGTGGGGCCAGGGAACCCTGGTCTGAAGAGCCCTCTGTTATTGCCGCC |
| VHCHR3_12Asn | CCAATACGGATGACGGGGCTCTTCAACCATAGGGGGCTTCTTTGACAACTGGGGCCAGGGAACCCTGGTCTGAAGAGCCCTCTGTTATTGCCGCC |
| VHCHR3_12Gln | CCAATACGGATGACGGGGCTCTTCAACCATAGGGGGCTTCTTTGACCAGTGGGGCCAGGGAACCCTGGTCTGAAGAGCCCTCTGTTATTGCCGCC |
| VHCHR3_12Asp | CCAATACGGATGACGGGGCTCTTCAACCATAGGGGGCTTCTTTGACGACTGGGGCCAGGGAACCCTGGTCTGAAGAGCCCTCTGTTATTGCCGCC |
| VHCHR3_12Glu | CCAATACGGATGACGGGGCTCTTCAACCATAGGGGGCTTCTTTGACGAATGGGGCCAGGGAACCCTGGTCTGAAGAGCCCTCTGTTATTGCCGCC |
| VHCHR3_12Lys | CCAATACGGATGACGGGGCTCTTCAACCATAGGGGGCTTCTTTGACAAATGGGGCCAGGGAACCCTGGTCTGAAGAGCCCTCTGTTATTGCCGCC |
| VHCHR3_12Arg | CCAATACGGATGACGGGGCTCTTCAACCATAGGGGGCTTCTTTGACCGTTGGGGCCAGGGAACCCTGGTCTGAAGAGCCCTCTGTTATTGCCGCC |
| VHCHR3_12His | CCAATACGGATGACGGGGCTCTTCAACCATAGGGGGCTTCTTTGACCACTGGGGCCAGGGAACCCTGGTCTGAAGAGCCCTCTGTTATTGCCGCC |
